# Prioritization of Mur family drug targets against *A*. *baumannii* and identification of their homologous proteins through molecular phylogeny, primary sequence, and structural analysis

**DOI:** 10.1186/s43141-020-00048-4

**Published:** 2020-07-28

**Authors:** Gizachew Muluneh Amera, Rameez Jabeer Khan, Rajat Kumar Jha, Amita Pathak, Jayaraman Muthukumaran, Amit Kumar Singh

**Affiliations:** 1 Department of Biotechnology, School of Engineering and Technology, Sharda University, Greater Noida, UP 201310 India; 2 Department of Chemistry, Indian Institute of Technology, Hauz Khas, New Delhi, 110016 India

**Keywords:** *Acinetobacter baumannii*, Molecular phylogeny, Primary sequence, Drug targets Secondary structure, 3D structure

## Abstract

**Background:**

The World Health Organization (WHO) report stated that *Acinetobacter baumannii* had been classified as one of the most important pathogenic bacteria causing nosocomial infection in hospital patients due to multi-drug resistance (MDR). It is vital to find out new bacterial drug targets and annotated their structure and function for the exploration of new anti-bacterial agents. The present study utilized a systematic route to prioritize the potential drug targets that belong to Mur family of *Acinetobacter baumannii* and identify their homologous proteins using a computational approach such as sequence similarity search, multiple sequence alignment, phylogenetic analysis, protein sequence, and protein structure analysis.

**Results:**

From the results of protein sequence analysis of eight Mur family proteins, they divided into three main enzymatic classes namely transferases (MurG, MurA and MraY), ligases (MurC, MurD, MurE, and MurF), and oxidoreductase (MurB). Based on the results of intra-comparative protein sequence analysis and enzymatic classification, we have chosen MurB, MurE, and MurG as the prioritized drug targets from *A*. *baumannii* and subjected them for further detailed studies of inter-species comparison. This inter-species comparison help us to explore the sequential and structural properties of homologous proteins in other species and hence, opens a gateway for new target identification and using common inhibitor for different bacterial species caused by various diseases. The pairwise sequence alignment results between *A. baumannii*’*s* MurB with *A. calcoaceticus*’*s* MurB, *A*. *baumannii*’*s* MurE with *A. seifertii*’*s* MurE, and *A*. *baumannii*’*s* MurG with *A. pittii*’*s* MurG showed that every group of the proteins are highly similar with each other and they showed sequence identity of 95.7% and sequence similarity of 97.2%.

**Conclusion:**

Together with the results of secondary and three-dimensional structure predictions explained that three selected proteins (MurB, MurE, and MurG) from *A*. *baumannii* and their related proteins (AcMurB, AsMurE, and ApMurG) belong to mixed *αβ* class and they are very similar.

## Background

*Acinetobacter* is a genus name which encompasses species that are strictly aerophilic, and biochemically catalase-positive, indole-negative, oxidase-negative, gram-negative, and citrate-positive [1] and its utmost essential representative is *A. baumannii* [2]. It has virulence factors that are responsible for different infections triggered by this organism. Cell surface hydrophobicity, outer membrane proteins (OMPs), toxic slime polysaccharides, and verotoxins are the possible virulence factors. From the virulence factors, cell surface hydrophobicity is a crucial element for bacterial sticking together as well as avoiding being phagocytosed [1, 3]. Extracellular enzymes named as esterases, certain amino-peptidases, acid phosphatases, and toxins which produce in the cytoplasm and other secreted substance are factors that play a substantial role in the pathogenesis, and it causes harm to host tissues mainly in the breathing system [1].

*A*. *baumannii* belongs to hospital-acquired pathogenic bacteria; therefore, the infections have been spread quickly in the hospital. The highest density of this infection occurred in the intensive care units (ICUs) in the hospitals [4]. On the other hand, the drug resistance ability of *A*. *baumannii* is also a global issue. The reports indicated that carbapenem-resistant strains are mostly responsible for epidemics. Additionally, these strains show intermediate resistance to tigecycline but vulnerability to colistin. Overall, this pathogenic bacteria have multiple mechanism for drug resistance ability to most of the drugs and they have the capacity to acquire the indicated drug resistance mechanisms ability within a short period of time [5]. According to the WHO, *A*. *baumannii* has been classified as one of the most important pathogenic bacteria causing hospital-acquired nosocomial infection due to MDR. Therefore, it is crucial to discover and prioritize the new and potential bacterial drug targets and subject them into structural and functional characterization, and facilitate to explore the new anti-microbial agents [6].

The bacterial cell is surrounded by a rigid structure called the cell wall. Cell wall in bacteria is made up of a polymeric network of peptidoglycan, which protects it from different environmental factors such as the excess amount of water in its surrounding that causes turgidity [7]. In the peptidoglycan pathway, the first step of the reaction is catalyzed using UDP-*N*-acetylglucosamine 1-carboxyvinyltransferase (MurA); it involves the attack at the electrophilic carbon two positions of phosphoenolpyruvate (PEP) leading to the cleavage of the carbon oxygen bond [8]. Similarly, the formation of UDP-*N*-acetylmuramic acid (UDPMurNAc) catalyzes using UDP-*N*-acetylenolpyruvoylglucosaminereductase (MurB) in the peptidoglycan biosynthetic pathway [8, 9]. Subsequently, the addition of a short polypeptide chain to the UDPMurNAc involves four key Mur ligase enzymes, namely MurC, MurD, MurE, and MurF. These four Mur ligases are responsible for the successive additions of l-alanine, d-glutamate, meso-diaminopimelate or l-lysine, and d-alanyl-d-alanine to UDP-*N*-acetylmuramic acid. All four Mur ligases are topologically similar to one another, even though they display low sequence identity [7, 10, 11]. The MraY is a membrane transferase enzyme that catalyzes the transfer of the phospho-*N*-acetylmuramoyl-pentapeptide motif onto the undecaprenyl phosphate carrier lipid [12]. The final steps of the peptidoglycan pathway, the transfer of a GlcNAc subunit on undecaprenyl-pyrophosphoryl-MurNAc-pentapeptide (lipid intermediate I) to form undecaprenyl-pyrophosphoryl-MurNAc-(pentapeptide) GlcNAc (lipid intermediate II) is catalyzed by MurG protein [13]. All of the enzymes are responsible for the synthesis of peptidoglycan in a sequential manner in bacteria, and this pathway is unique for them and it is not found in human [14]. So that, the prioritization of the enzymes involved in this pathway as a potential drug target is crucial to counterpart the severity of *A*. *baumannii* in hospital patients.

Nowadays, an identification, prioritization, and validation of suitable bacterial drug targets are crucial steps to identify or design new drug molecules against them. In connection with this, for the evaluation of gene function, essentiality, and suitability for drug development, the next generation sequencing have adequate and significant importance [15]. A few studies reported toward the identification, prioritization, and validation of drug target proteins from gram-negative pathogenic bacteria such as *Klebsiella pneumonia* [16], *Salmonella enterica* subsp. *enterica serovar* Poona [17], and *Pseudomonas aeruginosa* [18] by using different approaches. For computer-aided drug target identification, the Potential Drug Target Database (PDTD) is a very important resource and it is available at http://www.dddc.ac.cn/pdtd/. The database covers diverse information of more than 830 known or potential drug targets, including the information of protein and active sites structures in both PDB and MOL2 structural formats, related diseases, biological functions, as well as associated signaling pathways [19]. In this resource, TarFisDock (http://www.dddc.ac.cn/tarfisdock/) is used as a web server to identify the potential drug targets from the user given small molecule structure based on reverse docking approach. Apart from reverse docking approach, there are several approaches used to identify the drug targets, namely an integrative, multi-omics [16], computational subtractive genomics, molecular docking, virtual screening, [17], structural bioinformatics [15], and protein-protein interaction network [18].

The present study employed a systematic route to prioritize the pre-identified potential drug targets of Mur family from *A*. *baumannii* and identify their homologous proteins from other species using molecular phylogeny, primary sequence, secondary structure, and three-dimensional structural analysis. In this study, to our knowledge, first time, we used a combined molecular phylogeny with structural studies toward *A*. *baumannii* to prioritize the drug targets belonging to Mur family and identify other homologous drug target proteins in other species belonging to *Acinetobacter genus* using *A*. *baumannii* as reference organism. This methodology can also be used to prioritize and identify the drug targets in other bacteria caused by various diseases. This opens a new route to identify the effective antibacterial molecules which may act on multi-targeted proteins from various bacteria.

## Methods

### Primary sequence analysis of Mur family proteins

The amino acid sequence of Mur proteins involved in peptidoglycan biosynthetic pathway from *A*. *baumannii* were retrieved from UniProt database (https://www.uniprot.org/) which is a comprehensive collection of amino acid sequence and its associated information concerning sequence, structure, and function. The following set of Mur proteins were used in this study namely MurA (Accession No: B0V7N7), MurB (Accession No: B0V744), MurC (Accession No: B0V9F6), MurD (Accession No: B0VDD5), MurE (Accession No: A0A0R4J6I7), MurF (Accession No: A0A0R4J6Z4), MraY (Accession No: B0V8P2), and MurG (Accession No: B0V9F5).

### Physiochemical properties

To predict the physiochemical properties, each protein sequence of Mur member was subjected into protein sequence analysis using Expert Protein Analysis System (ExPASy) Proteomics web server (www.expasy.org/tools) [20]. The physicochemical properties such as amino acid composition, molecular weight (MW), iso-electric point (pI), instability index (II), aliphatic index (AI), and extinction coefficient (EC) were computed using ProtParam web server [20].

### Prediction of functional domains, subcellular localization and antigenic sites

The functional domain, subcellular localization, and antigenic sites of Mur family protein were predicted. To identify the functional domains present in the Mur family protein, we used the Simple Modular Architecture Research Tool (SMART) [21]. The subcellular localization of the protein was also predicted using PSort-B (subcellular localization prediction for bacteria) web server (http://psort.hgc.jp/) [22]. Finally, the possible antigenic sites of Mur proteins were predicted using the European Molecular Biology Open **S**oftware **S**uite (EMBOSS)-Antigenic program [23].

### Prediction of possible phosphorylation, glycosylation, and acetylation sites

In addition to the above-indicated parameters, the possible post-translation modifications (phosphorylation, glycosylation, and acetylation) sites of Mur family proteins were predicted by using MP Site (microbial phosphorylation site predictor) for phosphorylation [24], GLYCOPP v 1.0 for prediction of glycosylation sites in prokaryotes [25], and GPS-PAIL 2.0 software for acetylation [26].

### Intra-comparative sequence alignment, phylogenetic tree constructions, and analysis

The multiple protein sequence alignment (MSA) of these eight Mur family protein sequences from *A*. *baumannii* was performed by Clustal Omega server (https://www.ebi.ac.uk/Tools/msa/clustalo/), followed by the phylogenetic tree which was constructed using four different methods, namely unweighted pair group method with arithmetic mean (UPGMA), maximum parsimony (MP), minimum evolution (ME), and neighbor joining (NJ). The reason for choosing four different methods in phylogenetic analysis is to get more reliable details in terms of evolutionary distances, group identification, group classification, and similarity statistics among eight Mur family proteins. The statistical significance of the phylogenetic tree topology was evaluated by bootstrap analysis with 1000 iterative tree constructions using the software package named as Molecular Evolutionary Genetics Analysis Package (MEGA) Version X [27]. The following sequential and evolutionary details were obtained from aforementioned analysis namely (i) number of conserved sites (C), (ii) number of variable sites (V), (iii) number of parsimony informative sites (PI), (iv) number of singleton sites (S), (v) evolutionary distances, and (vi) overall mean average. Consensus tree construction using four different methods provided a reliable and reasonable evolutionary relationship among the input dataset of *A*. *baumannii* for further analysis.

### Inter-comparative sequence alignment, phylogenetic tree constructions, and analysis

Of the various enzymes involved in peptidoglycan pathway, we chose three Mur enzymes (MurB, MurE, and MurG) for detailed investigations based on enzymatic as well as functional classification. The proteins with significant amino acid sequence similarity to MurB, MurE, and MurG from *A*. *baumannii* were collected using position-specific iterated BLAST (PSI-BLAST or advanced BLAST or iterative BLAST) [28] search against Non-Redundant Protein Sequence Database (NRDB) at the National Centre for Biotechnology Information (NCBI) site (https://blast.ncbi.nlm.nih.gov/Blast.cgi). This iteration was running until it did not find any homologous sequences of the given query. After the results of PSI-BLAST, we prepared three input datasets (MurB with related sequences, MurE with related sequences, and MurG with related sequences) for further detailed analysis with the exclusion of the redundant, hypothetical, putative, predicted, and uncharacterized protein sequences. The multiple protein sequence alignment (MSA) was performed for three individual groups (MurB with related sequences, MurE with related sequences, and MurG with related sequences). Once MSA alignment was completed, the phylogenetic tree was constructed for the aforementioned three groups using five different methods namely UPGMA, MP, ME, NJ, and maximum likelihood (ML).

### Consensus secondary structure prediction

The secondary structural elements (Helix, Sheet, and Coil) of MurB, MurE, and MurG from *A*. *baumannii* as well as very closely related proteins were predicted using Consensus Secondary Structure Prediction (CSSP) [29]. Moreover, the secondary structural pattern of the MurB, MurE, and MurG, as well as their closely related protein sequences, was also investigated.

### Three-dimensional structure prediction

The three-dimensional structure of MurB, MurE, and MurG from *A*. *baumannii* was reported previously [7, 30]. Here, we used these same models from *A*. *baumannii* for comparative structural studies with their related protein models. In this study, we predicted the three-dimensional structure of MurB from *A. calcoaceticus*, MurE from *A. seifertii*, and MurG from *A. pittii* using Swiss Model web server [31] based on following three structural templates (PDB IDs: 4JAY, 4C12, and 1F0K, respectively) using homology modeling or comparative protein modeling approach. Three template structures share significant sequence identity, sequence similarity, and query coverage with the target sequences. Once three models are generated from Swiss model [31], they will be subsequently subjected into YASARA [32] web server for refinement of the protein structures followed by validation using Verify3D [33], Procheck [34], and ERRAT [35] which are available at Structure Analysis and Verification Server (SAVES) [36] web server. The optimized models were further used for structural superposition studies with previously reported models from *A*. *baumannii* using PyMOL [37] and FATCAT programs [38], respectively.

## Result

### Primary sequence analysis of Mur proteins

#### Physiochemical properties

From the protein sequence analysis results, the molecular weight ranged from 39.4 to 54.9 kDa and the theoretical pI of six Mur proteins (Accession IDs: B0V7N7, B0V744, B0V9F6, B0VDD5, A0A0R4J6I7, and A0A0R4J6Z4) is less than 6.18 but the remaining two Mur proteins (B0V8P2 and B0V9F5) are higher than 9.02. Based on the results of II values, the Mur family proteins have been thermodynamically stable. The II cut-off values indicated that if the protein is unstable, the II is greater than 40 and if the protein is stable, the II is less than 40. The ProtParam tool also computed the extinction coefficient for protein at a wavelength of 280 nm which is preferred because proteins absorb strongly here. Still, other substances are commonly found in other solutions. In the present results, the EC of the indicated protein at 280 nm measured in water ranged from 17,545 to 70,360 M^–1^ cm^–1^ concerning the concentration of Cys. The computed half-life of all of the Mur proteins is greater than 10 h. The relative volume of a protein occupied by aliphatic side chains (Ala, Val, Ile, and Leu) is regarded as the AI of a protein. This index is a decisive factor in enhancing the thermal stability of proteins. The theoretical AI of the indicated protein ranged from 90.78 to 126.32, which stated that the lower thermal stability of MurE is indicative of a more flexible structure when compared to others. Still, high AI of MraY infers that it may be stable for a wide range of temperature. The GRAVY value of the protein was ranged from − 0.225 to 0.781. The shallow GRAVY index of MurE indicated that this protein could have a better interaction with water (Table 1).

**Table 1:** The biological function of Mur proteins retrieved from UniProt database (https://www.uniprot.org/)

Accession No.	Pathways	Molecular function	Biological process
B0V7N7	Peptidoglycan biosynthesis	Transferase	Cellular division mainly cell wall biogenesis
B0V744	Peptidoglycan biosynthesis	Oxidoreductase	Cellular division, mainly cell wall organization
B0V9F6	Peptidoglycan biosynthesis	Ligase	Cellular division, mainly cell wall biogenesis
B0VDD5	Peptidoglycan biosynthesis	Ligase	Cellular division, mainly cell wall biogenesis
A0A0R4J6I7	Peptidoglycan biosynthesis	Ligase	Cellular division, mainly cell wall biogenesis
A0A0R4J6Z4	Peptidoglycan biosynthesis	Ligase	Cellular division, mainly cell wall biogenesis
B0V8P2	Peptidoglycan biosynthesis	Transferase	Cellular division, Mainly cell wall biogenesis
B0V9F5	Peptidoglycan biosynthesis	Glycosyltransferas, Transferase	Cellular division, mainly cell wall biogenesis and carbohydrate metabolic process

### Prediction of functional domains, subcellular localization, and antigenic site

According to the results of motif search using SMART, Mur family proteins have a minimum of 1 and maximum of 3 functional domains. Additionally, based on subcellular localization results, all of the protein resides in the bacterial cytoplasm except MraY and MurG, which are located in the cell inner membrane. MurA and MraY have only one functional domain. On the other hand, MurB and MurG have two functional domains, but the remaining Mur proteins have three functional domains. In the case of MurB, domain I (FAD-binding domain) starts from 30 to 164 amino acid residues. On the other hand, domain II (*C*-terminal domain) starts from 218 to 342 amino acid residues. Similarly, MurG has two domains, namely *N-*terminal (domain I) which begins from 12 to 149 amino acid residues and *C-*(domain II) terminal domain, which starts 194 to 365 amino acid residues. MurA has one domain which starts from 6 to 407 amino acid residues. Likewise, MraY domain begins from 45 to 372 amino acid sequence. In the peptidoglycan biosynthesis, the addition of a short polypeptide chain to the UDPMurNAc involves four key Mur ligase enzymes, namely MurC, MurD, MurE, and MurF. These four Mur ligases are responsible for the successive additions of l-alanine, d-glutamate, meso-diaminopimelate or l-lysine, and d-alanyl-d-alanine to UDP-N-acetylmuramic acid. All four Mur ligases are topologically similar to one another, even though they display low sequence identity. They are composed of three domains such as *N*-terminal Rossmann-fold domain responsible for binding the UDPMurNAc substrate, a central domain (similar to ATP-binding domains of several ATPasesand GTPases), and a *C*-terminal domain (similar to dihydrofolatereductase fold) that appears to be associated with binding the incoming amino acid.

Based on results obtained from EMBOSS-Antigenic, the Mur enzymes have several putative antigenic sites. The reason for predicting the antigenic sites is whether the predicted binding site residues (interactions with ligand molecules) belong to antigenic site or not. Since, antigenic sites are crucial for pathogenicity and virulence of the microorganism. In this study, we found 16, 22, 13, 14, 21, 19, 25, and 20 putative antigenic sites for MurB, MurE, MraY, MurG, MurD, MurF, MurC, and MurA, respectively and most of the antigenic sites are found in the binding pocket of concerned protein.

Together, the results obtained from aforementioned protein sequence analysis of eight Mur family proteins were divided into three main categories, namely transferases (MurG, MurA, and MraY), ligases (MurC, MurD, MurE, and MurG), and oxidoreductase enzymes (MurB) (Table 2). The biological process of these enzymes is cell division, regulation of cell shape, cell cycle, and the cell wall organization. They are using the same pathway known as peptidoglycan biosynthesis, which is a critical role for the formation of cell wall in prokaryotic organisms. The four (MurC, MurD, MurE, and MurF) Mur ligases are responsible for the successive additions of l-alanine, d-glutamate, meso-diaminopimelate or l-lysine, and d-alanyl-d-alanine to UDP-N-acetylmuramic acid in the peptidoglycan pathways (Table 2).

**Table 2:** The primary protein sequence analysis of eight Mur family proteins from *A*. *baumannii*

Gene Name	Accession No.	Protein sequence analysis											
		AA	Mw (KDa)	pI	GRAVY	II	AI	D	EC	HL (h)	L	+ R	− R
MurA	B0V7N7	418	44.6	5.12	0.117	26.61	104.55	1	17545	>10	Cyto	(Arg + Lys) 39	(Asp + Glu) 55
MurB	B0V744	353	39.7	6.18	− 0.161	31.19	99.94	2	39880	>10	Cyto	(Arg + Lys) 30	(Asp + Glu) 35
MurC	B0V9F6	482	52.8	5.64	− 0.172	31.16	98.32	3	18005	>10	Cyto	**(**Arg + Lys) 49	(Asp + Glu 62
MurD	B0VDD5	455	48.6	6.14	0.042	35.90	105.47	3	19285	>10	Cyto	(Arg + Lys) 43	(Asp + Glu) 48
MurE	A0A0R4J6I7	499	54.9	5.48	− 0.225	32.23	90.78	3	47120	>10	Cyto	(Arg + Lys) 41	(Asp + Glu) 58
MurF	A0A0R4J6Z4	466	50.8	5.87	− 0.130	33.75	93.45	3	25565	>10	Cyto	(Arg + Lys) 37	(Asp + Glu) 49
MraY	B0V8P2	372	40.9	9.54	0.781	25.10	126.32	1	70360	>10	CIM	(Arg + Lys) 26	(Asp + Glu) 17
MurG	B0V9F5	365	39.4	9.02	0.051	39.12	97.34	2	20190	>10	Cyto	(Arg + Lys) 34	(Asp + Glu) 28

*AA* amino acid, *Mw* molecular weight, *pI* isoelectric point, *GRAVY* grand average of hydropathicity, *II* instability index, *AI* aliphatic index, *D* domain, *EC* extinction coefficients, *L* location, + *R* positively charged residues, − *R* negatively charged residues

### Prediction of possible phosphorylation, glycosylation, and acetylation sites

The possible acetylation sites of MurA, MurB, MurC, MurD, MurE, MurF, MraY, and MurG have 4 (Lys3, Lys11, Lys413, and Lys415), 1 (Lys3), 6 (Lys11, Lys12, Lys15, Lys99, Lys133, and Lys482), 3 (Lys7, Lys205, and Lys364), 1 (Lys114), 2 (Lys125 and Lys465), 1 (Lys342), and 5 (Lys8, Lys10, Lys205, Lys345, and Lys364), respectively. In addition to this, these enzymes have 4 (3 Ser-dependent and 1 Thr-dependent residue), 3 (1 Ser-dependent and 2 Thr-dependent residues), 8 (5 Ser-dependent and 3 Thr-dependent residues), 1 (1 Ser-dependent and 0 Thr-dependent residues), 4 (3 Ser-dependent and 1 Thr-dependent residue), 12 (5 Ser-dependent and 7 Thr-dependent residues), 1 (0 Ser-dependent and 1 Thr-dependent residue), and 4 (3 Ser-dependent and 1 Thr-dependent residue) phosphorylation sites, respectively and finally, the number of residues involved in glycosylation of MurA, MurB, MurC, MurD, MurE, MurF, MraY, and MurG are 17 (1 *N*-linked and 16 *O*-linked) residues, 12 (6 *N*-linked and 6 *O*-linked) residues, 20 (5 *N*-linked and 15 *O*-linked) residues, 21 (4 *N*-linked and 17 *O*-linked) residues, 31 (6 *N*-linked and 25 *O*-linked) residues, 28 (2 *N*-linked and 26 *O*-linked) residues, 21 (3 *N*-linked and 18 *O*-linked) residues, and 15 (4 *N*-linked and 11 *O*-linked) residues, respectively, with the significant prediction score (Supplementary section-Table 3) which indicated that Mur proteins might also undergo these modifications; however, further experimental studies are necessary to validate this hypothesis.

**Table 3:** Amino acid composition (in %) of eight Mur family proteins from *A*. *baumannii*

Amino acids	Accession number							
	B0V7N7	B0V744	B0V9F6	B0VDD5	A0A0R4J6I7	A0A0R4J6Z4	B0V8P2	B0V9F5
Ala	11.2	7.1	8.7	10.3	11.4	11.2	9.4	13.4
Arg	4.5	2.3	6.0	3.7	4.8	4.1	3.2	3.8
Asn	3.1	7.1	5.0	3.5	4.6	3.4	3.2	4.1
Asp	6.0	4.8	5.8	5.5	6.4	4.5	2.4	3.6
Cys	0.5	0.3	0.6	1.3	0.8	0.4	0.3	1.4
Gln	1.9	8.2	4.4	5.9	7.2	7.9	3.0	8.2
Glu	7.2	5.1	7.1	5.1	5.2	6.0	2.2	4.1
Gly	9.3	6.2	8.5	9.5	6.4	8.2	8.6	6.8
His	2.4	3.4	3.1	2.4	3.6	4.1	1.9	1.9
Ile	6.2	6.8	7.3	5.5	6.4	6.0	8.1	5.2
Leu	10.8	10.5	9.5	12.5	9.0	10.9	14.5	9.6
Lys	4.8	6.2	4.1	5.7	3.4	3.9	3.8	5.5
Met	3.3	1.4	2.3	2.2	1.6	2.8	4.8	3.3
Phe	2.4	5.1	3.1	2.4	3.8	4.1	5.1	3.3
Pro	3.6	3.7	4.1	4.0	3.6	3.6	3.2	5.8
Ser	3.8	4.5	4.4	5.3	5.6	5.2	5.6	2.7
Thr	7.4	4.0	5.2	4.4	5.8	6.2	4.6	6.0
Trp	0.2	1.1	0.0	0.2	1.0	0.6	2.4	0.5
Tyr	1.9	3.4	2.5	2.0	2.6	1.3	3.8	1.6
Val	9.3	8.8	8.3	8.6	6.6	5.6	9.9	9.0

### Intra-comparative protein sequence analysis of Mur family proteins

In the present study, the classification of Mur family proteins was carried out using comparative protein sequence analysis approach, according to sequence level similarity, identity, and pairwise distances; this provides information about the evolutionary relationship among Mur proteins from *A*. *baumannii*. This study includes multiple sequence alignment followed by phylogenetic analysis.

### Multiple sequence alignment and phylogenetic tree construction

The results of multiple sequence alignment of Mur proteins from *A*. *baumannii* indicated that there is a significant variation among the input dataset (Fig. S16). The results of our comparative protein sequence alignment showed that there are four essential sites present in the Mur enzymes, namely conserved, variable, singleton, and parsimony-informative and their statistics are 4/520, 481/520, 275/520, and 195/520, respectively. The overall mean average of input dataset of eight Mur proteins is 2.29. The results of MSA indicated that there is a divergence relationship among the Mur proteins but all Mur ligases are topologically similar to one another, even though they display low sequence identity, similarity and pairwise distance. We observed an overall mean average of our input dataset of eight Mur proteins showing around 2.29, which indicated that dataset has diverged when we are comparing all at once. In contrast, they are nearly similar when we were comparing one with others in the input dataset of eight Mur proteins.

The pairwise distance analysis indicated that the minimal distances were noticed in between MurD and MurC; on the other hand, the maximal distances were observed between MurB and MurG (Tables 4 and 5). According to the results of pairwise distance, two broad groups were found, and it indicates that MurC and MurD are highly similar with the score of 1.66 compared to other; in contrast, MurG and MurB are distant with the values of 2.86. In general, MurB is less similar to other proteins in the groups, and hence it is considered as out-group from this analysis. The MurC, MurD, MurE, and MurF belongs to the same group (group I), explained that they might have involved similar functional role concerning the synthesis of peptidoglycan. Additionally, MurG, MurA, and MraY are nearly similar to each other. Overall results conclude that selecting of MurE, MurB, and MurG are reasonable for further detailed studies for inter-species comparison.

**Table 4:** Hydrophilic and hydrophobic residues content of eight Mur family proteins from *A*. *baumannii*

Accession No.	Hydrophobic residues (%)	Hydrophilic residues (%)	Net hydrophobic residues content
B0V7N7	45.8	18.4	High
B0V744	44.5	22.7	High
B0V9F6	42.3	22.3	High
B0VDD5	45	19.3	High
A0A0R4J6I7	43.2	20	High
A0A0R4J6Z4	42.9	19.1	High
B0V8P2	58.3	15.3	Very High
B0V9F5	47.3	21.1	High

**Table 5:** Pairwise distance statistics of eight Mur proteins from *A*. *baumannii*

S/No.	MurA	MurB	MurC	MurD	MurE	MurF	MraY	MurG
1	MurA	2.62	2.21	2.47	2.40	2.32	2.29	2.41
2	MurB		2.53	2.19	2.20	2.23	2.64	2.86
3	MurC			1.66	1.84	1.70	2.47	2.21
4	MurD				1.79	1.95	2.35	2.42
5	MurE					1.94	2.66	2.34
6	MurF						2.41	2.43
7	MraY							2.44
8	MurG							

Moreover, the inter-species comparison will help us to explore the sequential and structural properties of homologous proteins in other species and hence opens a gateway for novel antibacterial therapeutics using the common inhibitor for drug targets in different species caused for various diseases. This result also support the previous primary sequence analysis results concerning the classification of enzyme according to the function of the proteins.

**Fig. 1:**
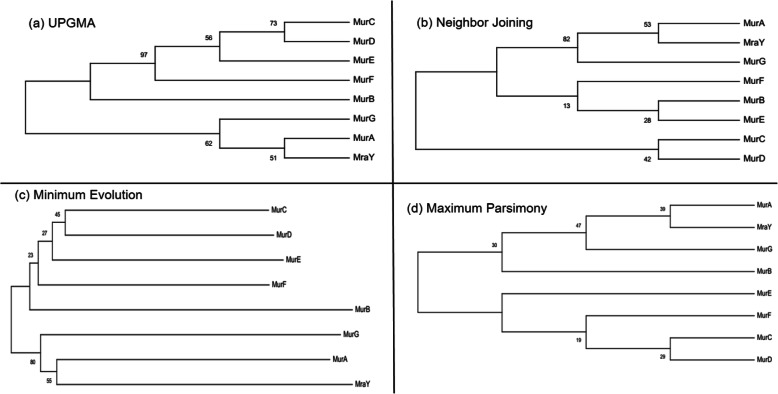
Phylogenetic tree construction of eight Mur proteins from *A*. *baumannii*. Tree was constructed by **a** UPGMA, **b** neighbor-joining, **c** minimum evolution, and **d** maximum parsimony methods. Topology was also evaluated by bootstrap analysis via MEGA X. The numerical values in the tree represent bootstrap results

The phylogenetic analysis was performed for eight Mur proteins from *A. baumannii* using NJ, ME, UPGMA, and MP methods. All ambiguous positions were removed for each sequence pair (pairwise deletion option). There were a total of 520 sequence sites that were observed in the final dataset. Evolutionary analyses were conducted using MEGA X [27], indicating that there are two major groups (group I contains MurC, MurD, MurE, MurF, and MurB and the group II comprises of MurG, MurA, and MraY). MurB is very far from the first group as well as the second group. Group I was divided into two subgroups which are representing the following members, subgroup 1 (SG1) and subgroup 2 (SG2). SG1 represents the following members such as MurE, MurD, MurF, and MurC; on the other hand, SG2 includes MurB. Group II also contains SG1 and SG2. The SG1 represents MurG and SG2 include MurA and MraY. From the results obtained from phylogenetic analysis (Fig. 1), it has been observed that MurC, MurD, MurF, and MurE are very similar with each other and fall in two same groups but MurB is considered as out-group. On the other hand, MurG, MurA, and MraY are similar to each other so that selecting one protein for further study from each group and subgroup may be representing the others concerning common structural and functional relationship.

### Inter-comparative protein sequence alignment, phylogenetic tree construction, and analysis

Based on the results of primary sequence analysis and phylogenetic tree construction of Mur members, we have selected MurE from ligase group, MurG from transferase group, and finally MurB from oxidoreductase group for further analysis.

### MurB with closely related sequences

**Fig. 2:**
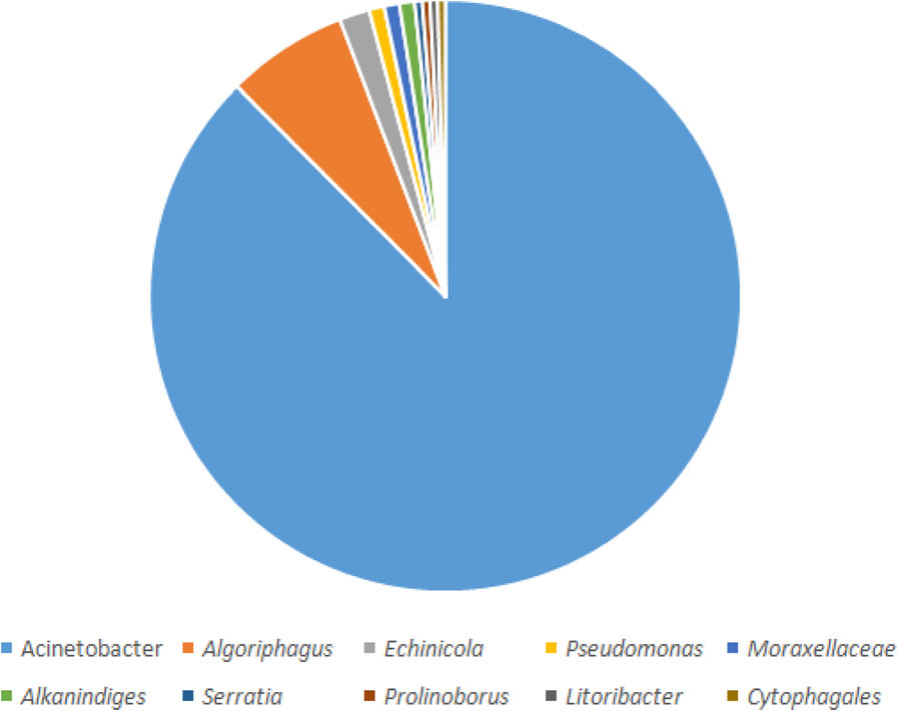
Similarity pie diagram of MurB from *A*. *baumannii* and its related sequences from various genus obtained from PSI-BLAST search against NRDB

**Fig. 3:**
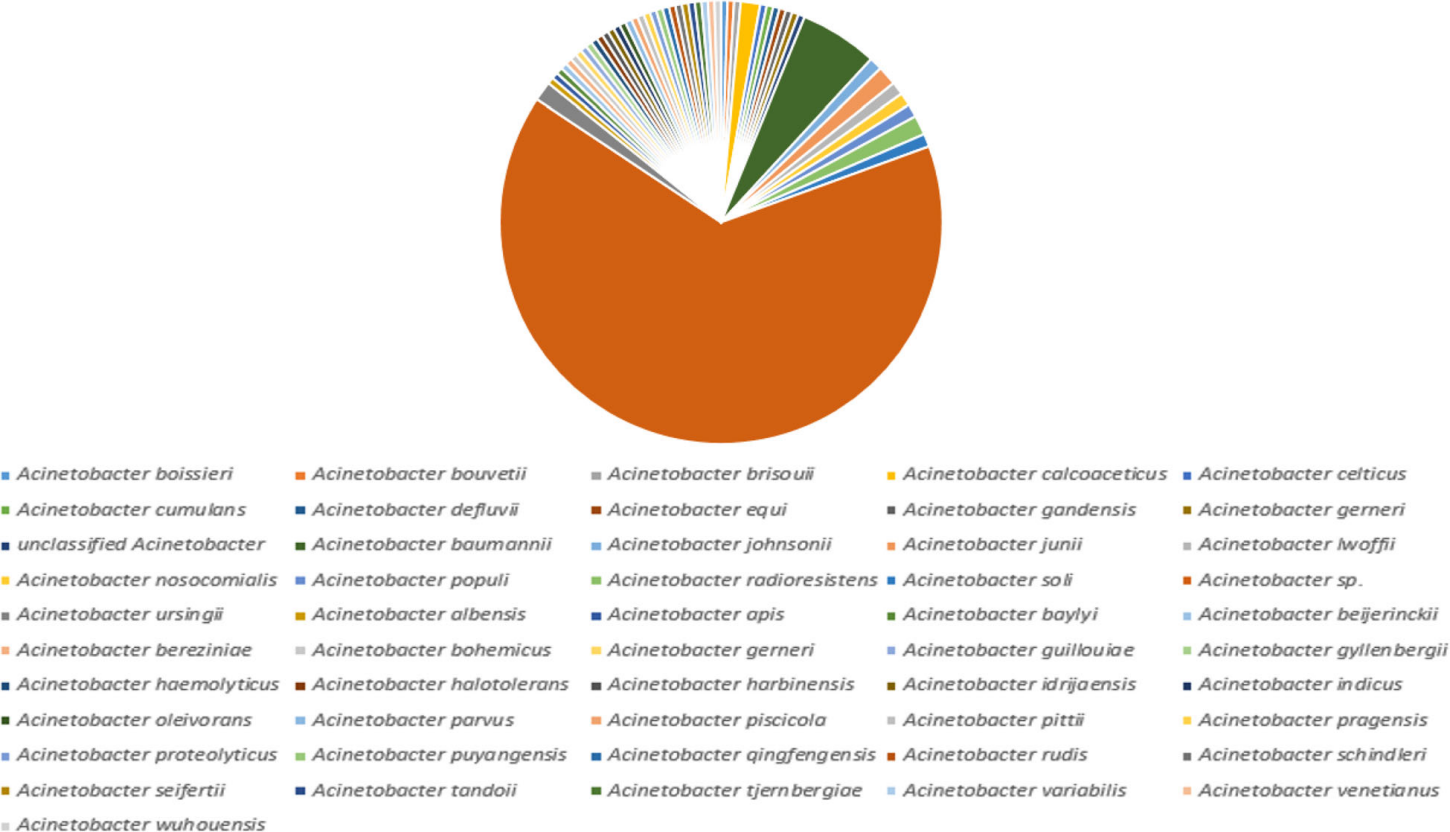
Similarity pie diagram of MurB from *A*. *baumannii* and its related sequences from various *Acinetobacter* species obtained from iterative BLAST search

The MurB protein sequence was retrieved from the UniProt database (https://www.uniprot.org/) and submitted into PSI-BLAST (https://blast.ncbi.nlm.nih.gov/Blast.cgi) against NRDB until no newly protein sequences are displayed during the cycle of PSI-BLAST. After completing the seven cycles of search of MurB against NRDB using PSI-BLAST, we identified 241 homologous sequences. Out of 241 protein sequences, 211belongs to the genus of *Acinetobacter*, 16 belongs to the genus of *Algoriphagus*, 4 belongs to the genus of *Echinicola*, 2 belongs to the genus of three species (*Pseudomonas*, *Moraxellaceae*and *Alkanindiges*), and finally, 1 belong to the genus of four species (*Serratia*, *Prolinoborus*, *Litoribacter*, and *Cytophagales*) (Fig. 2). The amino acid sequences of most of the genus perform a similar function as MurB which are responsible for the peptidoglycan biosynthetic pathways. Since our interest is to understand the evolutionary relationship of *Acinetobacter* species, particularly *baumannii*, further screening are directed toward 211 genus alone, which are further classified based on species alone (Fig. 3). Finally, we have obtained 51 different species from the genus of *Acinetobacter* which are closely related to the *A*. *baumannii*. The statistical information of various species related to MurB from *A*. *baumannii* was presented in Figs. 2 and 3.

### Multiple sequence alignment and phylogenetic tree construction

**Fig. 4:**
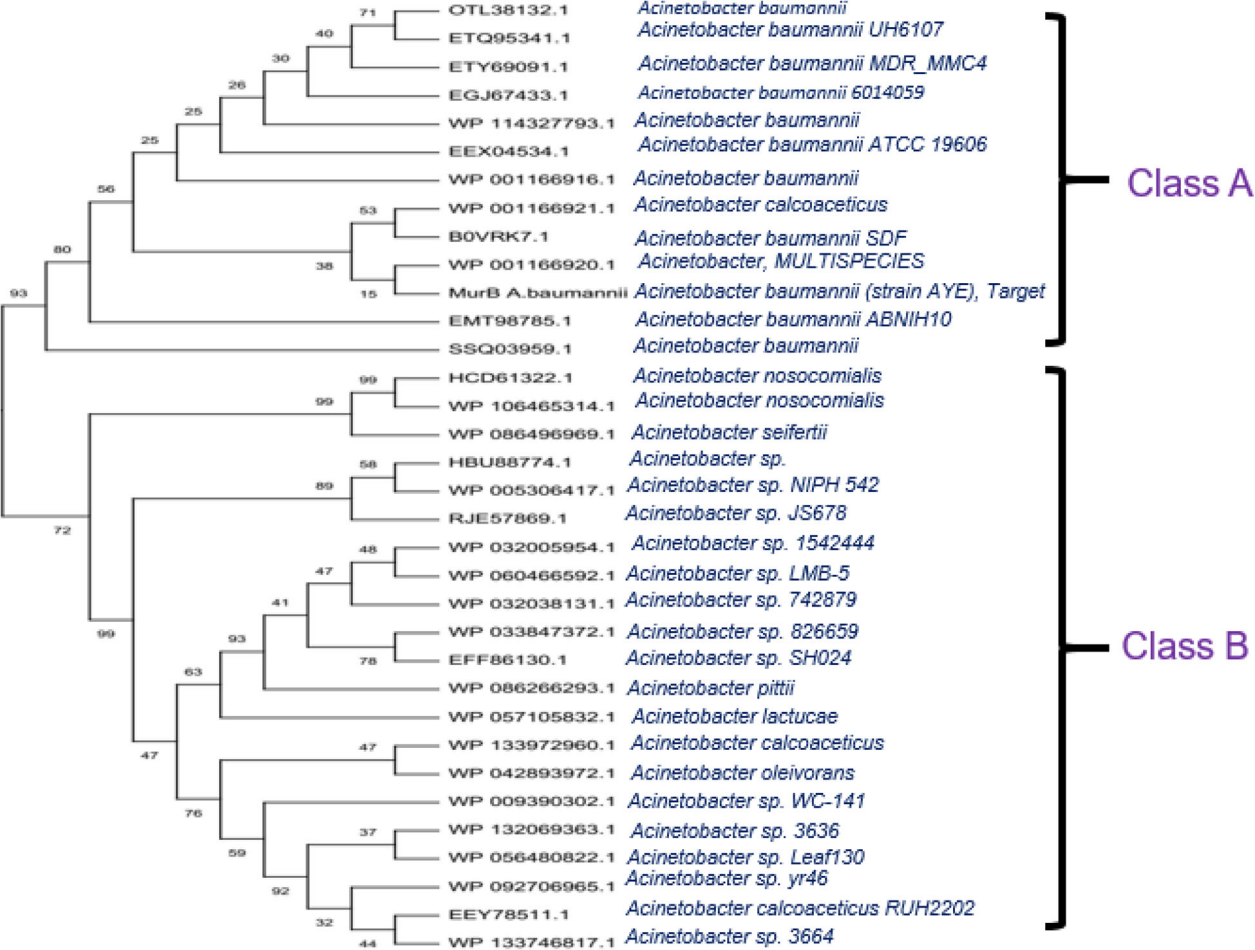
Phylogenetic tree construction of MurB from *A*. *baumannii* with related sequences. The tree was prepared by neighbor-joining method. For the clarity, part of the tree was presented here

The results of multiple sequence alignment of MurB protein from *A*. *baumannii* with its related sequences indicated that there is a sequence variation among them. The results of our comparative protein sequence alignment showed that there are four significant sites are present in the input data set, namely conserved, variable, singleton, parsimony-informative, and their statistics were 68/399, 303/399, 17/399, and 286/399 respectively. The overall mean average of this dataset was found to be 0.45. Overall results of MSA followed by phylogenetic analysis explained that there is a significant relationship among the input dataset of MurB and its related entries. The phylogenetic analysis was performed using five different methods. For the clarity, we discussed the results obtained from the NJ method (Fig. 4) in the main-text and remaining was presented in supplementary section (Figs. S1-S5). Evolutionary analyses are conducted in MEGA X [27] which indicated that two major groups are observed in this phylogenetic tree (Figs. S1-S5). But according to our study, we are focusing on the groups and protein sequences nearest to our target, i.e., MurB from *A*. *baumannii* is presented here. This group is also further divided into two main subclasses, namely class A and class B. Since the MurB protein sequence from *A*. *baumannii* was located in class A, we considered this class for detailed investigation. In this class, we observed the protein sequences mostly from *A*. *baumannii* and interestingly one MurB from *A*. *calcoaceticus*.

**Fig. 5:**
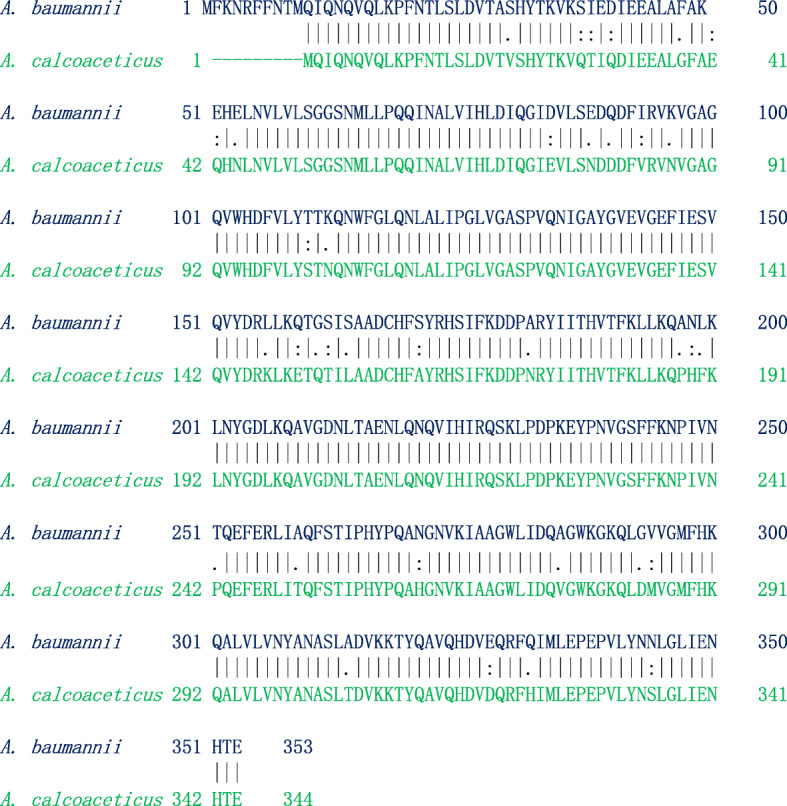
Pairwise sequence alignment of MurB protein from *A*. *baumannii* and MurB from *A*. *calcoaceticus*

From the results of phylogenetic analysis, we observed one very closely related sequence of MurB from *A*. *calcoaceticus*, which is found in the same clade where MurB of *A*. *baumannii* existed. To understand the structural and functional relationship between two proteins, we attempted detailed sequence and structural analysis, and these results were presented here (Fig. 5). The pairwise sequence alignment results between MurB protein from *A*. *baumannii* and *A*. *calcoaceticus* showed that both the proteins are highly similar with the sequence identity of 97.5% and sequence similarity of 97.2%, respectively. The physicochemical parameters of *A*. *baumannii*’*s* MurB (AA = 353, Mw (kDa) = 39.7, pI = 6.18, II = 31.19, AI = 99.94, GRAVY = − 0.161), and *A*. *calcoaceticus*’*s* MurB (AA = 344, Mw (kDa) = 38.6, pI = 5.94, II = 32.99, AI = 102.56, GRAVY = − 0.149) are nearly similar. Moreover, the subcellular localization of both proteins is cytosol. Similarly, both the proteins sharing a common domain with the position of 21–155 amino acid residues in domain I and 209–333 amino acids is domain II. Based on the results obtained from sequence analysis, we conclude that both the proteins from two different species are highly similar. Hence, one common molecule might bind to the active site of MurB from two different species. Together with *A*. *baumannii*, *A*. *calcoaceticus* is commonly known as *A*. *calcoaceticus*–*A*. *baumannii complex*. It can be pathogenic, and route causes opportunistic infection in patients with multiple underlying diseases. Our preliminary analysis opens the gateway to treat the infections caused by two different bacteria; the former one is a hospital-acquired nosocomial pathogen and later on, an opportunistic pathogen.

### MurE with closely related sequences

**Fig. 6:**
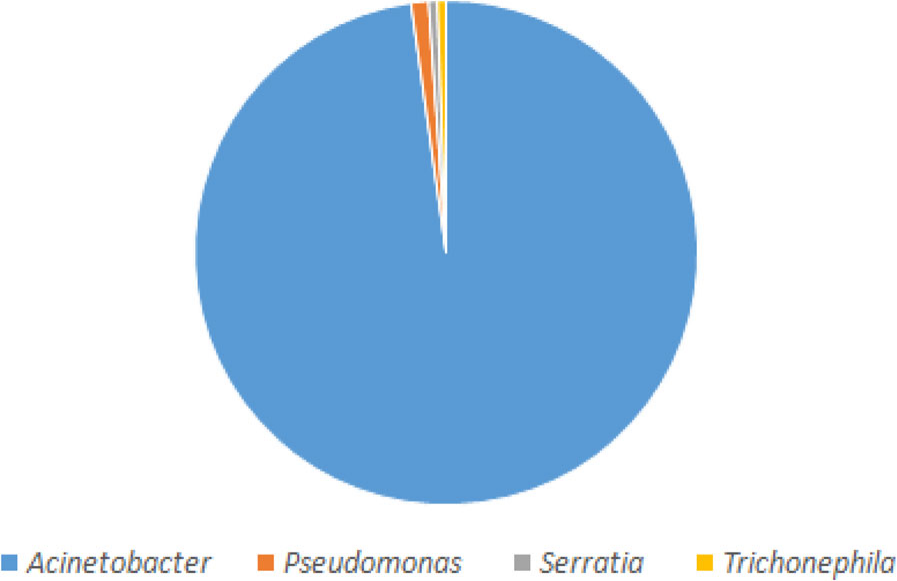
Similarity pie diagram of MurE from *A*. *baumannii* and its related sequences from various genus obtained from PSI-BLAST search against NRDB

**Fig. 7:**
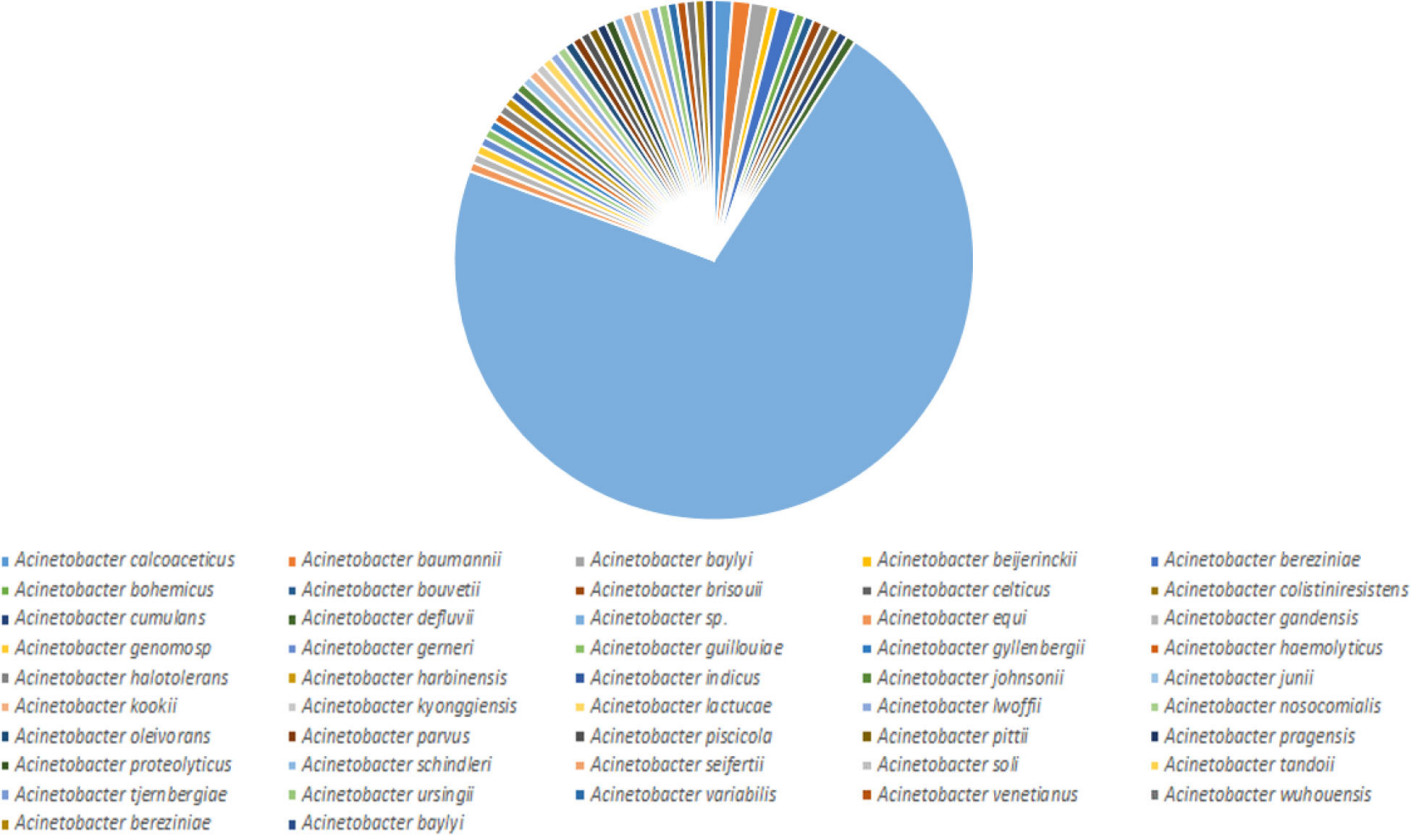
Similarity pie diagram of MurE from *A*. *baumannii* and its related sequences from various *Acinetobacter* species obtained from iterative BLAST search

The protein sequence of MurE was obtained from the UniProt database (https://www.uniprot.org/) and subsequently subjected to PSI-BLAST (https://blast.ncbi.nlm.nih.gov/Blast.cgi?PAGE=Proteins) search against NRDB until no newly protein sequences are displayed during the iterations of PSI-BLAST. After finishing the five cycles of PSI-BLAST search of MurE, we identified 179 protein sequences from various species. Out of 179 related sequences of MurE, the majority of entries belong to the genus of *Acinetobacter* (175 in total). However, few entries belong to the genus of *Pseudomonas* (2 in total), *Serratia* (1 in total) and *Trichonephila* (1 in total). Interestingly, we observed one ESKAPE pathogen from this phylogenetic analysis named as *Pseudomonas*, which is also showing MDR property as *Acinetobacter*. Statistical division of MurE from *A*. *baumannii* and its related sequence neighbors was presented in Fig. 6. Out of 175 related protein sequences of *Acinetobacter*, we observed diverse types of *Acinetobacter* species which is around 47 entries (Fig. 7).

### Multiple sequence alignment and phylogenetic tree construction

**Fig. 8:**
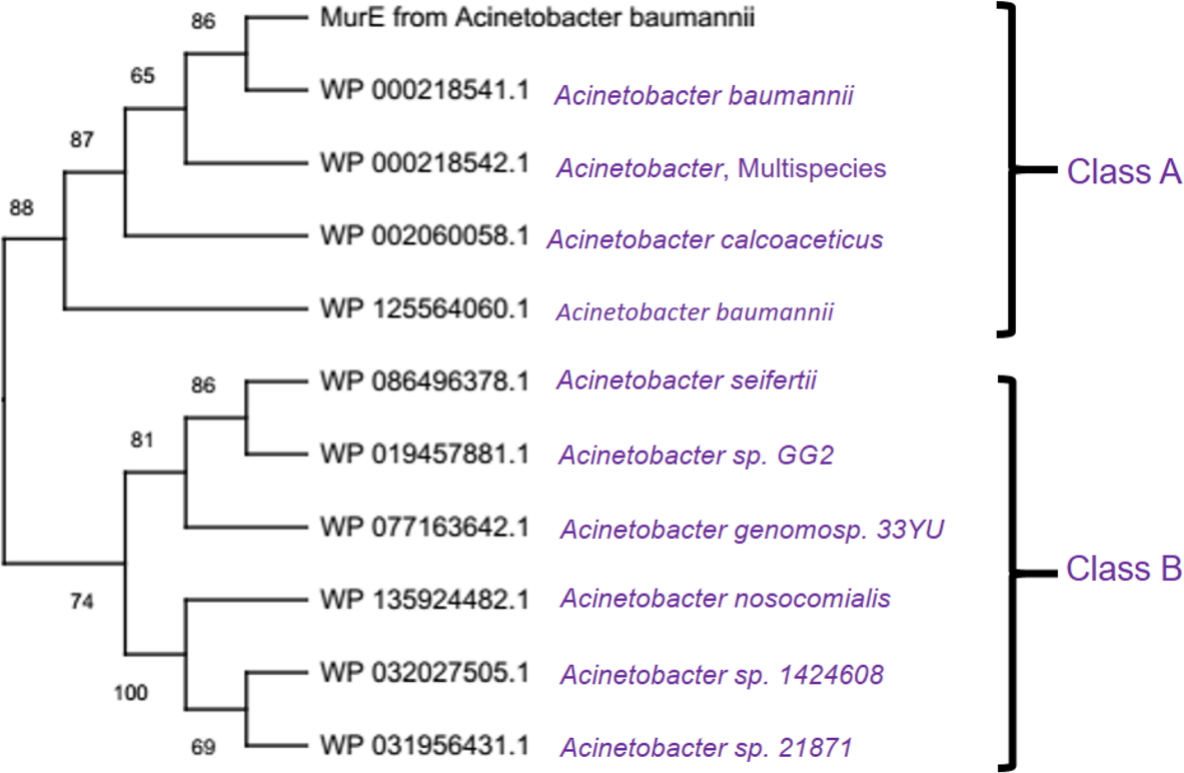
Phylogenetic tree construction of MurE from *A*. *baumannii* with related protein sequences using NJ method. For the clarity, part of the tree was presented here

The results of multiple sequence alignment of MurE proteins from *A*. *baumannii* with its related sequences indicated that there is a sequence variation among them. The statistics of conserved, variable, singleton, and parsimony-informative sites are 168/1085, 347/1085, 51/1085, and 296/1085, respectively. On the other hand, the overall mean average of this input dataset was found to be 0.27. Overall results of MSA indicated that there is a good relationship among the input dataset of MurE and its related entries. The phylogenetic analysis was performing using MEGA X [27], which indicated that there are two major groups in this cladogram (Figs. S6–S10). But according to our study, we are focusing on the groups and protein sequences nearest to our target, i.e., MurE from *A*. *baumannii* is presented here. This group is also divided into two main subclasses, namely class A and class B. Since the MurE protein sequence from *A*. *baumannii* was located in class A, we considered this class for detailed investigation. In this class, we observed the protein sequences mostly from *A*. *baumannii* and interestingly one from *A*. *calcoaceticus*. We already found the relationship between *A*. *baumannii* and *A*. *calcoaceticus* through phylogenetic analysis of MurB with its related sequences (discussed in the previous section). This organism is again observed in this phylogenetic tree (Fig. 8) as well, which is located in class A. To find the drug target in another *Acinetobacter* species, we observed *A. seifertii* in the neighboring clade (class B). Therefore, we used MurE from *A*. *seifertii* for a detailed investigation followed by comparison with MurE from *A*. *baumannii*.

**Fig. 9:**
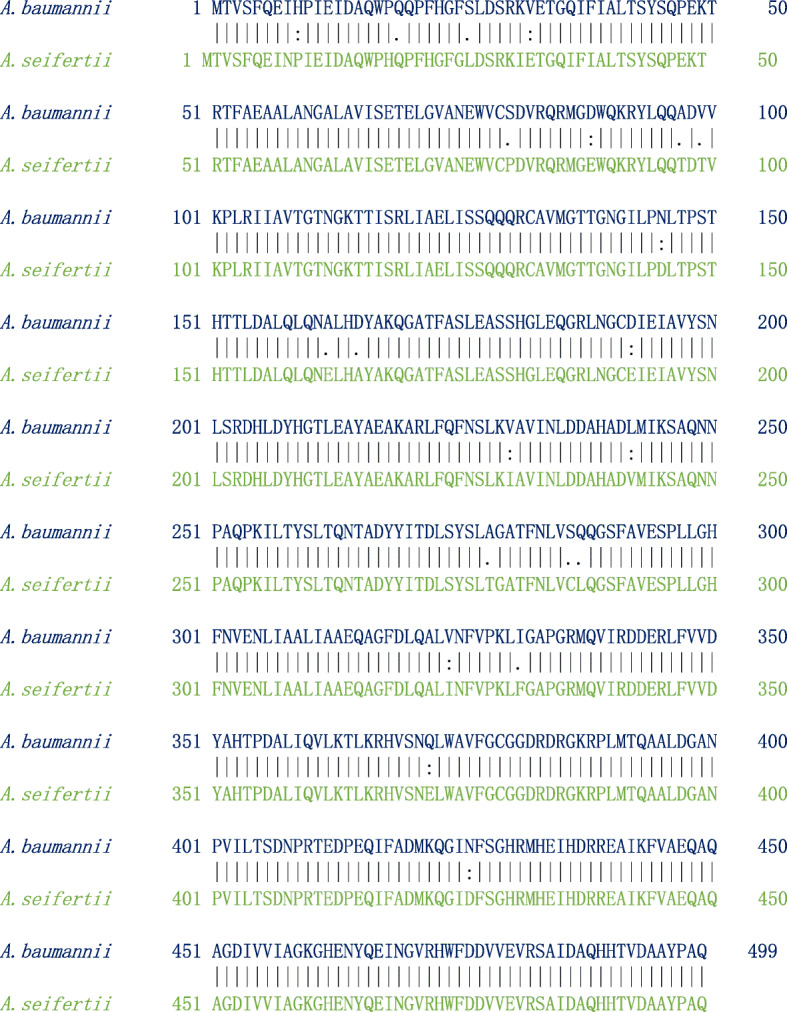
Pairwise sequence alignment of MurE protein from *A*. *baumannii* and MurE protein *A*. *seifertii*

The pairwise sequence alignment results between MurE protein from *A*. *baumannii* and *A*. *seifertii* showed that both the proteins are highly similar (sequence identify: 95.8% and sequence similarity: 97.8%) (Fig. 9). The physicochemical parameters of *A*. *baumannii*’*s* MurE (AA = 499, Mw (kDa) = 54.98, pI = 5.48, II = 32.23, AI = 90.78, GRAVY = − 0.225) and *A*. *seifertii*’*s* MurE (AA = 499, Mw (kDa) = 55.12, pI = 3.31, II = 31.99, AI = 90.20, GRAVY = − 0.226) are nearly similar. Moreover, the subcellular localization of two protein was found to be cytosol and similarly both the protein sharing common domain. Based on the results obtained from sequence analysis, we conclude that both the proteins from two different species are highly similar. Hence, one common molecule might act on the active site of MurE from two different species and thus lead to be a gateway for potent antibacterial therapeutics.

### MurG with closely related sequences

**Fig. 10:**
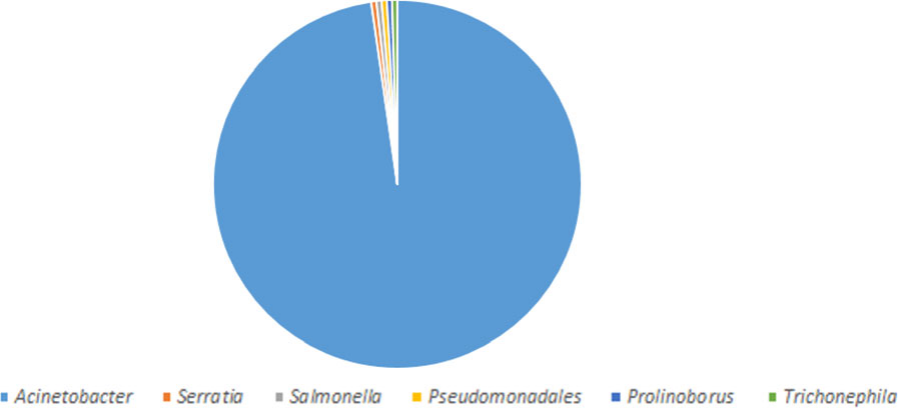
Similarity pie diagram of MurG from *A*. *baumannii* and its related sequences from various genus obtained from PSI-BLAST search against NRDB

**Fig. 11:**
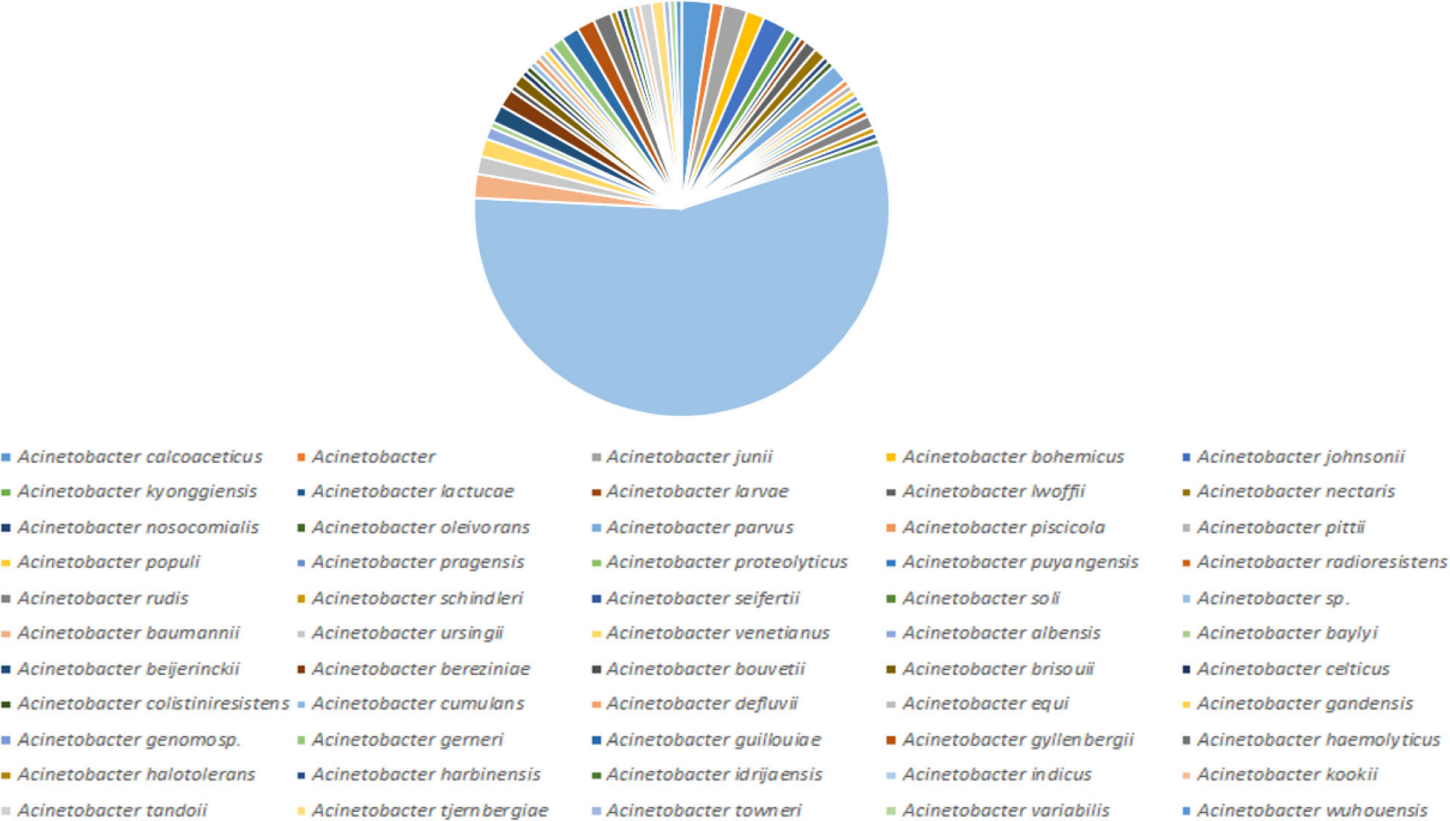
Similarity pie diagram of MurG from *A*. *baumannii* and its related sequences from various *Acinetobacter* species obtained from iterative BLAST search

The MurG protein sequence was retrieved from the UniProt database (https://www.uniprot.org/) and submitted into PSI-BLAST (https://blast.ncbi.nlm.nih.gov/Blast.cgi) against NRDB until no newly protein sequences are displayed during the iteration cycle of PSI-BLAST. After finishing the six cycles of MurG search against NRDB database, we observed 220 homologous protein sequences. Out of 220 amino acid sequences, 215 belong to the genus of *Acinetobacter*, and the remaining genus is *Serratia*, *Salmonella*, *Pseudomonadales*, *Prolinoborus*, and *Trichonephila* (Fig. 10). Out of 215 genera of *Acinetobacter*, finally, we have obtained 55 different species which are strictly related to the *A*. *baumannii*. The statistical information of various species related to MurG from *A*. *baumannii* was presented in Figs. 10 and 11.

### Multiple sequence alignment and phylogenetic tree construction

**Fig. 12:**
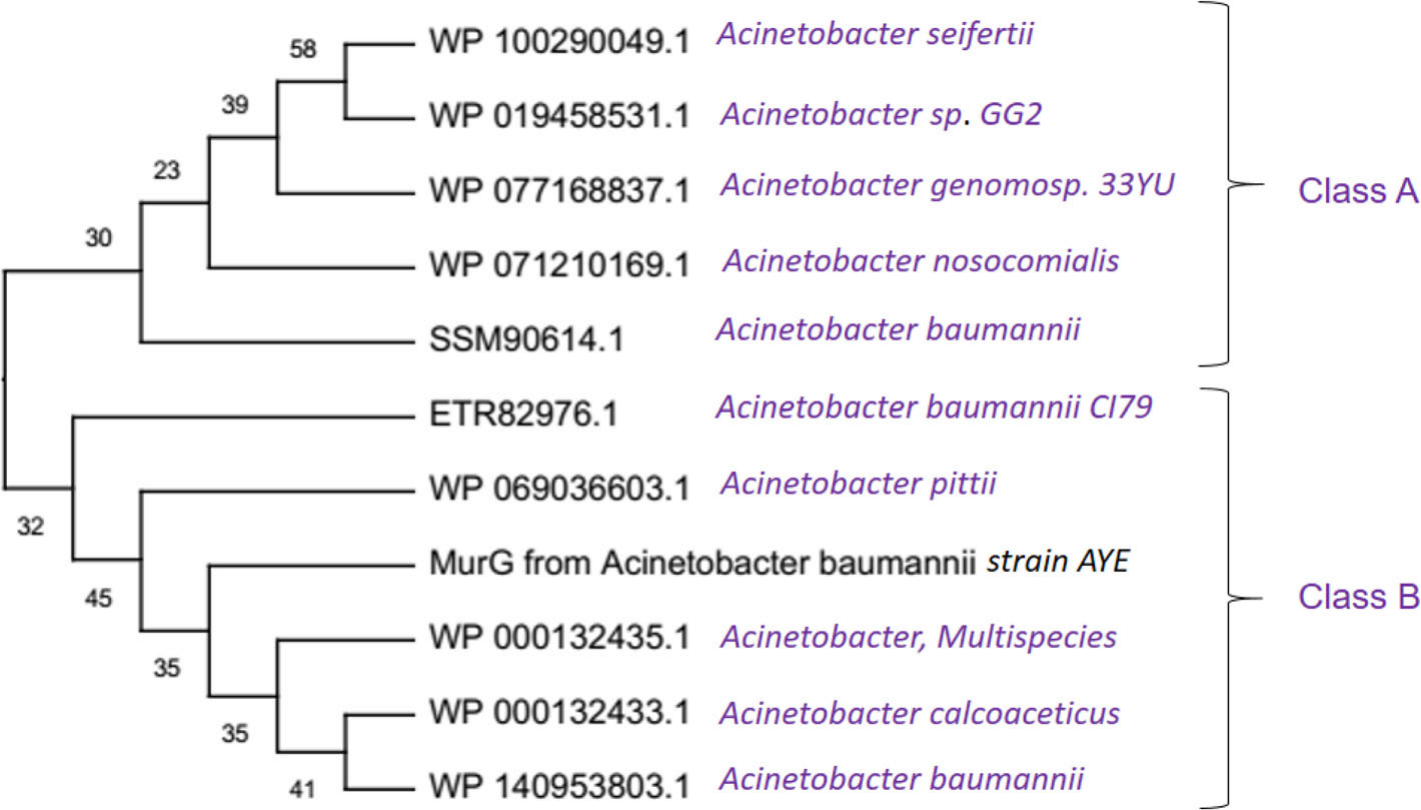
Phylogenetic tree construction of MurG from *A*. *baumannii* with related protein sequences using NJ method. For the clarity, part of the tree was presented here

The results of comparative protein sequence alignment of MurG protein from *A. baumannii* with its homologous sequences indicated that there is a sequence variation among them. From the results of multiple protein sequence alignment, we found that 402 sites in the final dataset of MurG and its related sequences and the statistics of conserved, variable, singleton, and parsimony-informative sites are 141, 230, 23 and 207, respectively. The overall mean distance of this dataset was found to be 0.21. Results of MSA indicated that there is a good relationship among the input dataset of MurG and its related entries. Evolutionary analyses were carried out using MEGA X [27] indicating that there are two major groups in this cladogram (Figs. S11–S15). But according to our study, we are focusing on the groups and protein sequences nearest to our target, i.e., MurG from *A. baumannii* is presented here. This group also classified into two main divisions, namely class A and class B. Since the MurG protein sequence from *A. baumannii* was located in class B, we considered this division for detailed investigation. In this division, we observed that the protein sequence that is nearest to *A. baumannii* is *A. pittii* (Fig. 12).

**Fig. 13:**
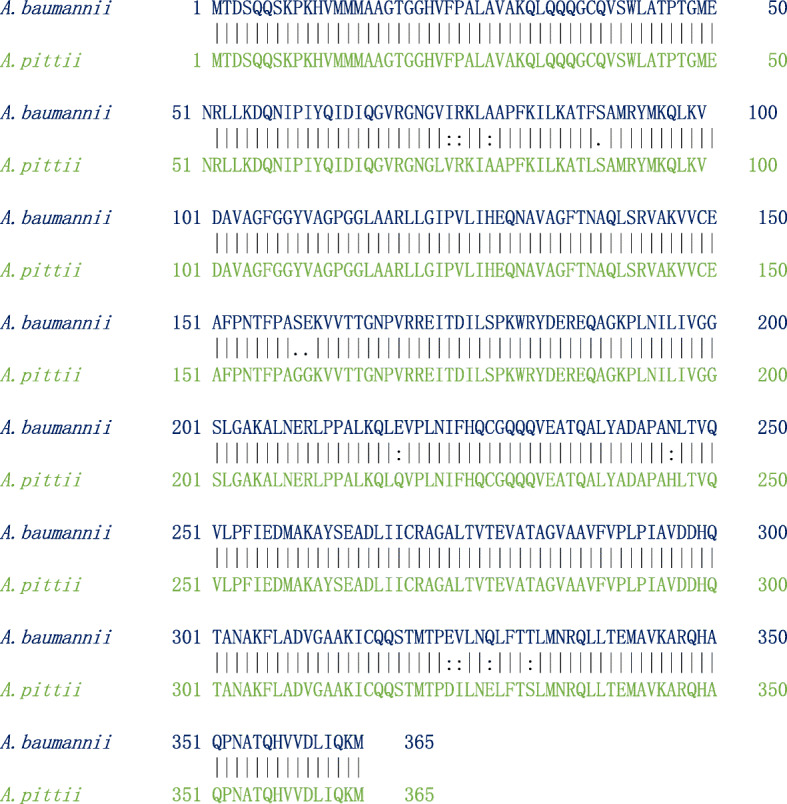
Pairwise sequence alignment of MurG protein from *A*. *baumannii* and *A*. *pittii*

The pairwise sequence alignment results between MurG protein from *A*. *baumannii* and *A*. *pittii* showed that both the proteins are highly similar with the identity of 96.7% and similarity of 99.2%, respectively (Fig. 13). The physicochemical parameters of *A*. *baumannii*’*s* MurG (AA = 365, Mw (kDa) = 39.35, pI = 9.02, II = 39.12, AI = 97.34, GRAVY = 0.051) and *A*. *pittii*’*s* MurG (AA = 365, Mw (kDa) = 39.22, pI = 9.13, II = 38.87, AI = 98.68, GRAVY = 0.065) are nearly similar. Moreover, the subcellular localization of MurG from *A*. *baumannii* is the cytoplasm; in contrast, MurG protein from *A*. *pittii* is found to be cell membrane. Both these proteins shared a common domain. Based on the results obtained from sequence analysis, we conclude that both the proteins from two different species are highly similar (Fig. 13).

### Consensus sequence-based secondary structure predication

**Fig. 14:**
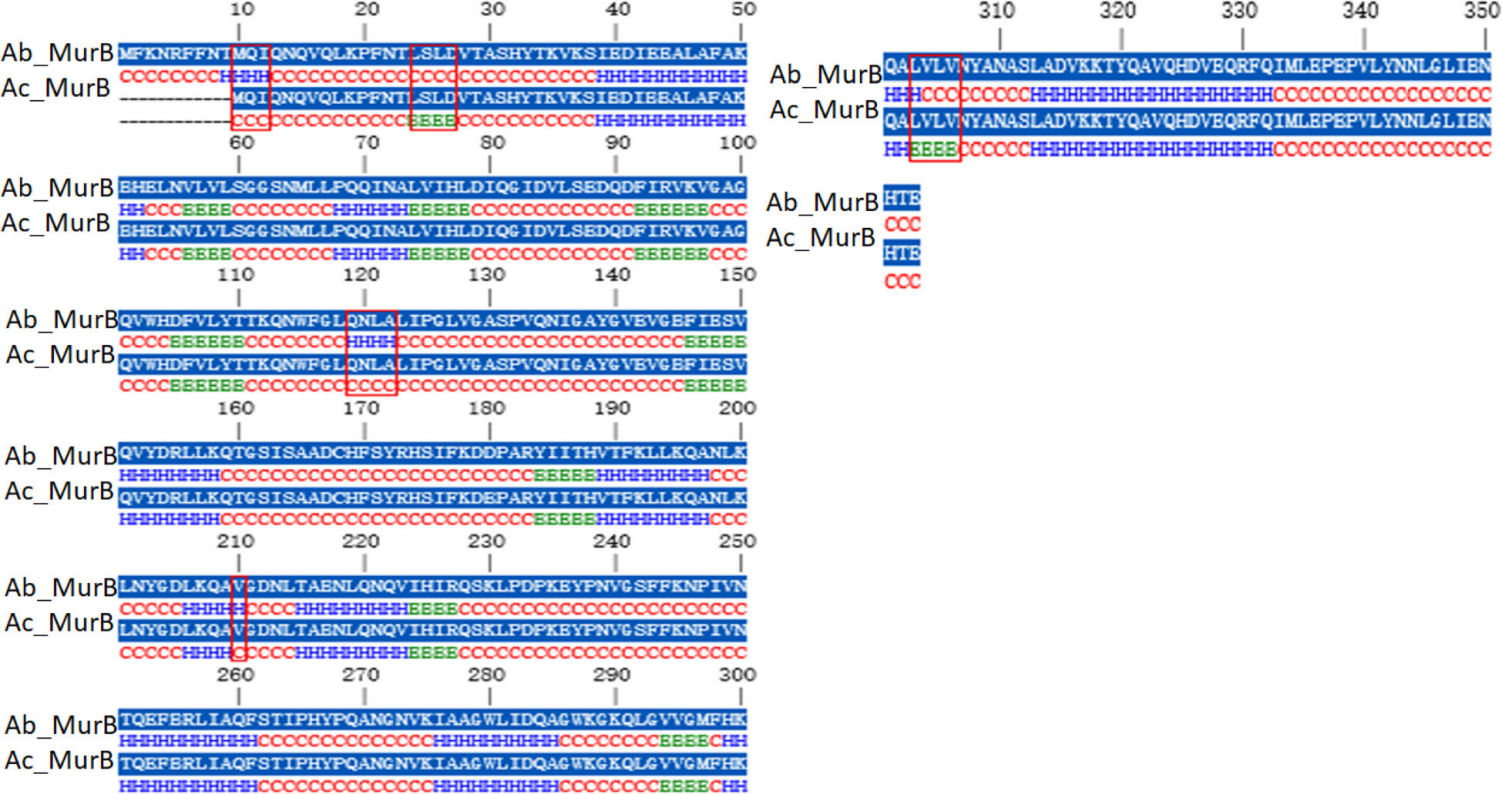
Conserved secondary structure prediction and comparison of MurB from *A*. *baumannii* and *A*. *calcoaceticus.* Structurally variable regions in between them were highlighted using red boarded rectangular box

**Fig. 15:**
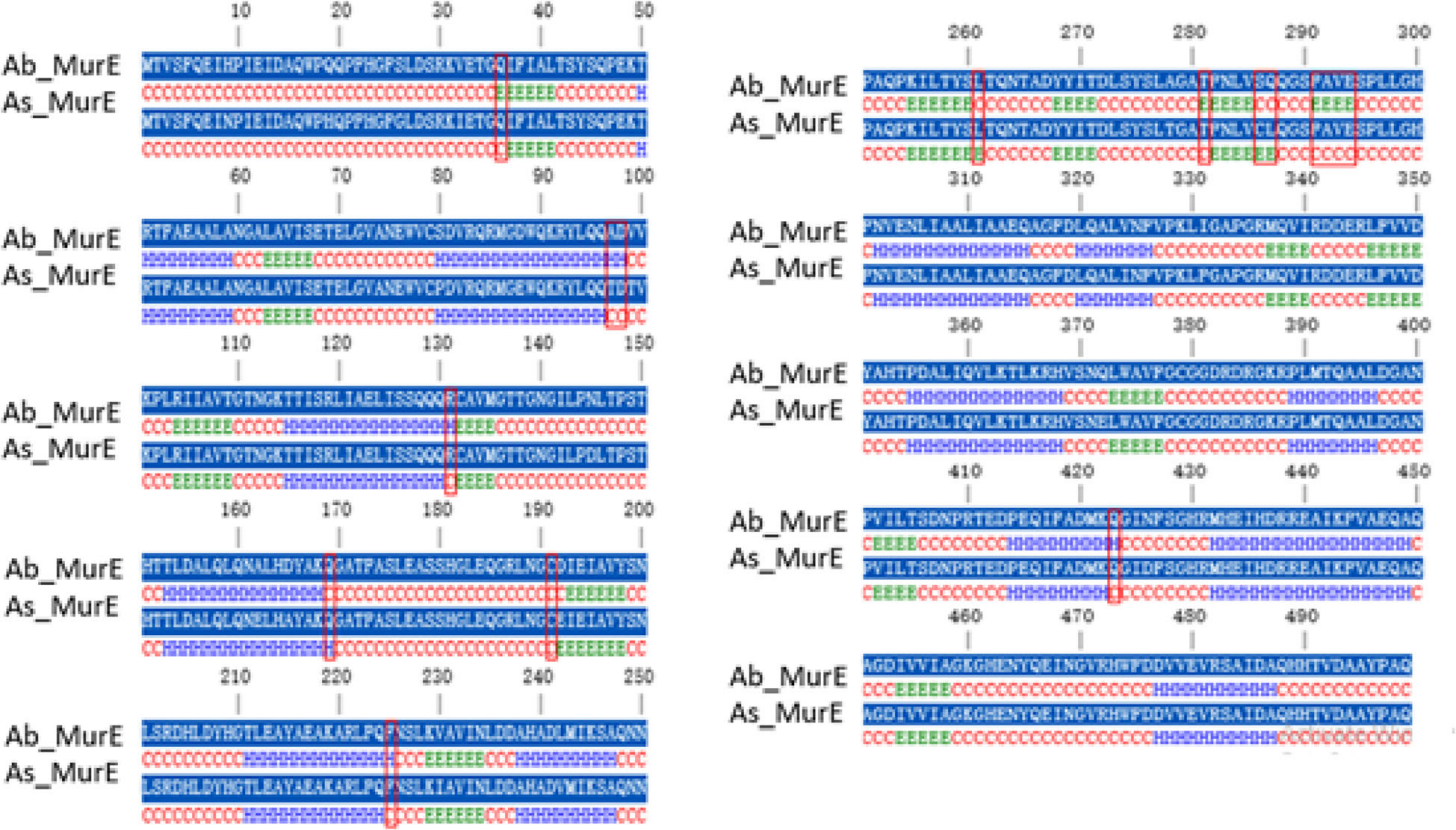
Conserved secondary structure prediction and comparison of MurE from *A*. *baumannii* and *A*. *seifertii.* Structurally variable regions in between them were highlighted using red boarded rectangular box

**Fig. 16:**
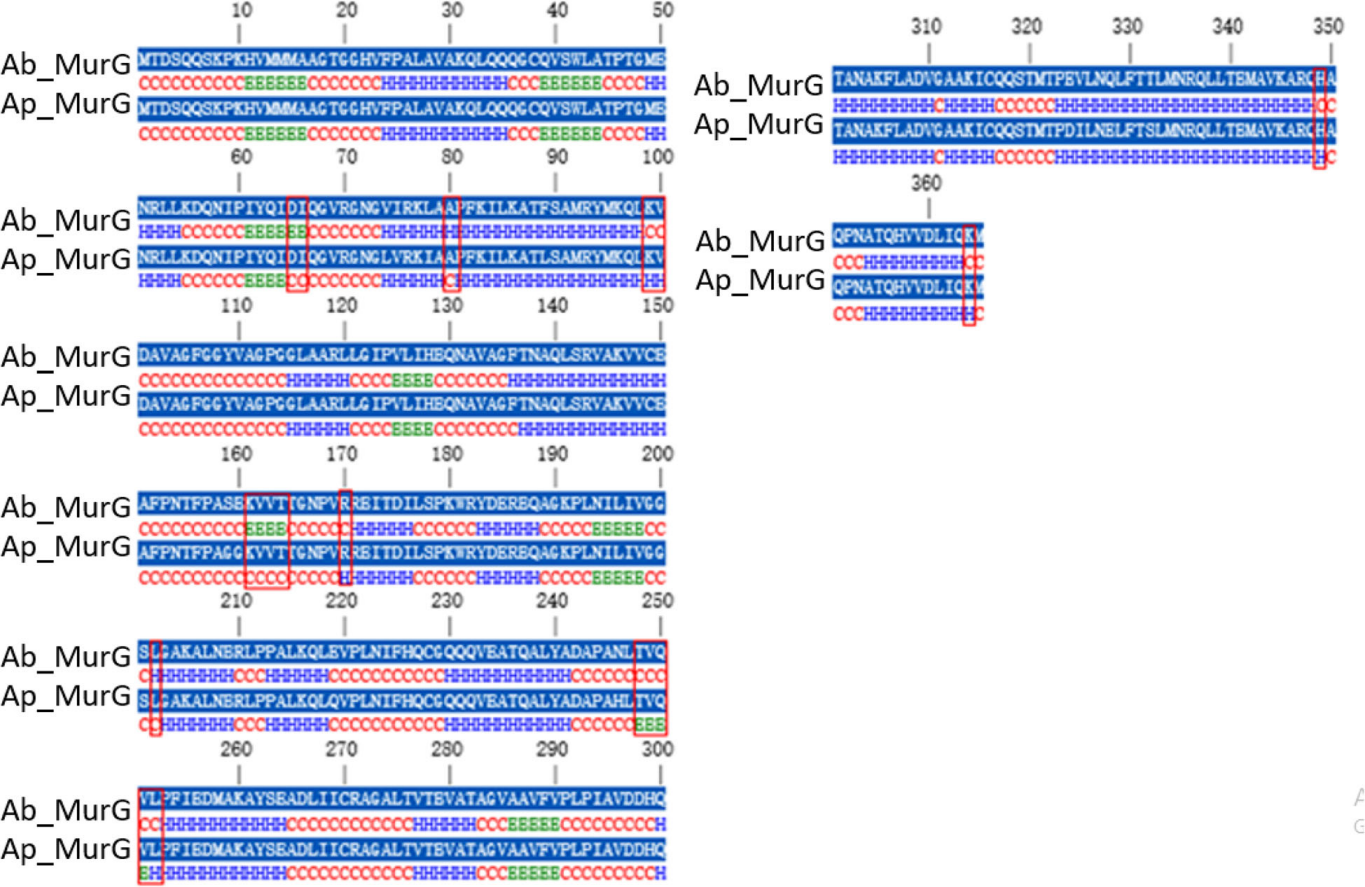
Conserved secondary structure prediction and comparison of MurG from *A*. *baumannii* and *A*. *pittii.* Structurally variable regions in between them were highlighted using red boarded rectangular box

The secondary structural study is significant as it provides direct imminent into the functional role of a protein and used for the structural classification of a specified protein. Comparative sequence analysis of MurB protein from *A*. *baumannii* and *A*. *calcoaceticus* can lead to identifying the similarity at the sequence level alone. To find out the structural similarity of these two enzymes, we need to have the secondary and tertiary structure of both the proteins, thus will be helped to understand the proteins in a particular way. For the prediction of the secondary structure of MurB from both the species, we used Consensus Secondary Structure Prediction (CSSP), which is available at http://bioserver1.physics.iisc.ernet.in/cgi-bin/cssp/run_cssp.pl [29]. CSSP web server observed that both the proteins belong to mixed *αβ* class protein. The composition of alpha and beta sheets for both the protein was found to be 29.74% and 11.05% for *A*. *baumannii*, whereas 27.62% and 13.66% for *A*. *calcoaceticus* (Fig. 14). These results conclude that the secondary structure composition of both the proteins is nearly similar, which corroborate with previous findings of primary sequence analysis followed by phylogenetic analysis. As MurB, the secondary structure of MurE from *A*. *baumannii* and *A*. *seifertii* was predicted through CSSP, which explained that both these proteins again belong to mixed *αβ* class protein. The composition of alpha and beta sheets for both these proteins was found to be 33.8% and 15% for *A*. *baumannii*, whereas 33% and 14.6% for *A*. *seifertii* (Fig. 15). The secondary structural classes of MurG from *A*. *baumannii* and *A*. *pittii* belong to mixed αβ class. The alpha and beta sheets compositions of MurG from two different species are 47.1% and 9.9% for *A*. *baumannii* and 47.9% and 9.3% for *A*. *pittii*, respectively (Fig. 16). The alpha-helical composition of MurG (from both species) is higher than MurB and MurE (from both the species). In summary, the consensus secondary structure prediction results revealed that the secondary structure of MurB, MurE, and MurG from *A*. *baumannii* is similar with the related MurB (*A*. *calcoacetic*), MurE (*A*. *seifertii*), and MurG (*A*. *pittii*) proteins.

### Three-dimensional structure predication of selected three Mur targets and their related proteins

For understanding the functional relationship of three Mur proteins from *A*. *baumannii* with their related members namely *A*. *calcoaceticus*, *A*. *seifertii*, and *A*. *pittii*, we attempted three-dimensional structure prediction of selected three related proteins using a comparative protein modeling approach. The three-dimensional structure of MurB, MurE, and MurG from *A*. *baumannii* was reported previously [7, 30]. Here, we used those models from *A*. *baumannii* for comparative structural studies with their related protein models. In this study, we predicted the three-dimensional structure of MurB from *A*. *calcoaceticus*, MurE from *A*. *seifertii*, and MurG from *A*. *pittii* based on following structural templates (PDB Accession No: 4JAY, 4C12 and 1F0K, respectively) using a comparative protein modeling approach using Swiss Model web server [31]. The structural templates were selected by using Protein BLAST against Protein Data Bank (www.rcsb.org). The template selection is again conformed with the results obtained from the first step of Swiss Model [28].

The sequence similarity search against PDB database of MurB amino acid sequence from *A*. *calcoaceticus* revealed that 4JAY was the best template with the query coverage of 96%, sequence identity of 41.25%, and a statistical significance of 4e-87 compared to the other homologous sequences obtained from the Protein BLAST search. Thirty percent identity exists in between the query and template is sufficient cut-off to perform the comparative or homology modeling. In the case of MurE from *A*. *seifertii*, based on the sequence similarity search against PDB database, two templates including 4C12 (33.05%, 91%, and 2e-65) and 1E8C (35.95%, 92%, and 2e-71) were selected on the basis of sequence identity, query coverage and E (statistical significance) value, respectively. But, according to further selection criteria such as crystal structure resolution (1.80 Å for 4C12 and 2.00 Å for 1E8C) and model quality (the protein was modeled in both the templates but the model built by 4C12 had better model evaluation results), 4C12 was selected for the model building processes. The template selection for MurG from *A*. *pittii* showed that on the basis of sequence similarity search, two templates namely 3S2U (49.16%, 95%, and 2e-109) and 1F0K (44.13%, 97%, and 3e-90) were selected on the basis of sequence identity, query coverage, and E (statistical significance) value, respectively. But, according to further selection criteria such as resolution (the resolution 1.9 Å for 1F0K and 2.23 Å for 3S2U) and model quality, 1F0K was selected for the model building processes. The three-dimensional structure of these three models belongs to mixed *αβ* class as Mur proteins from *A*. *baumannii*.

**Fig. 17:**
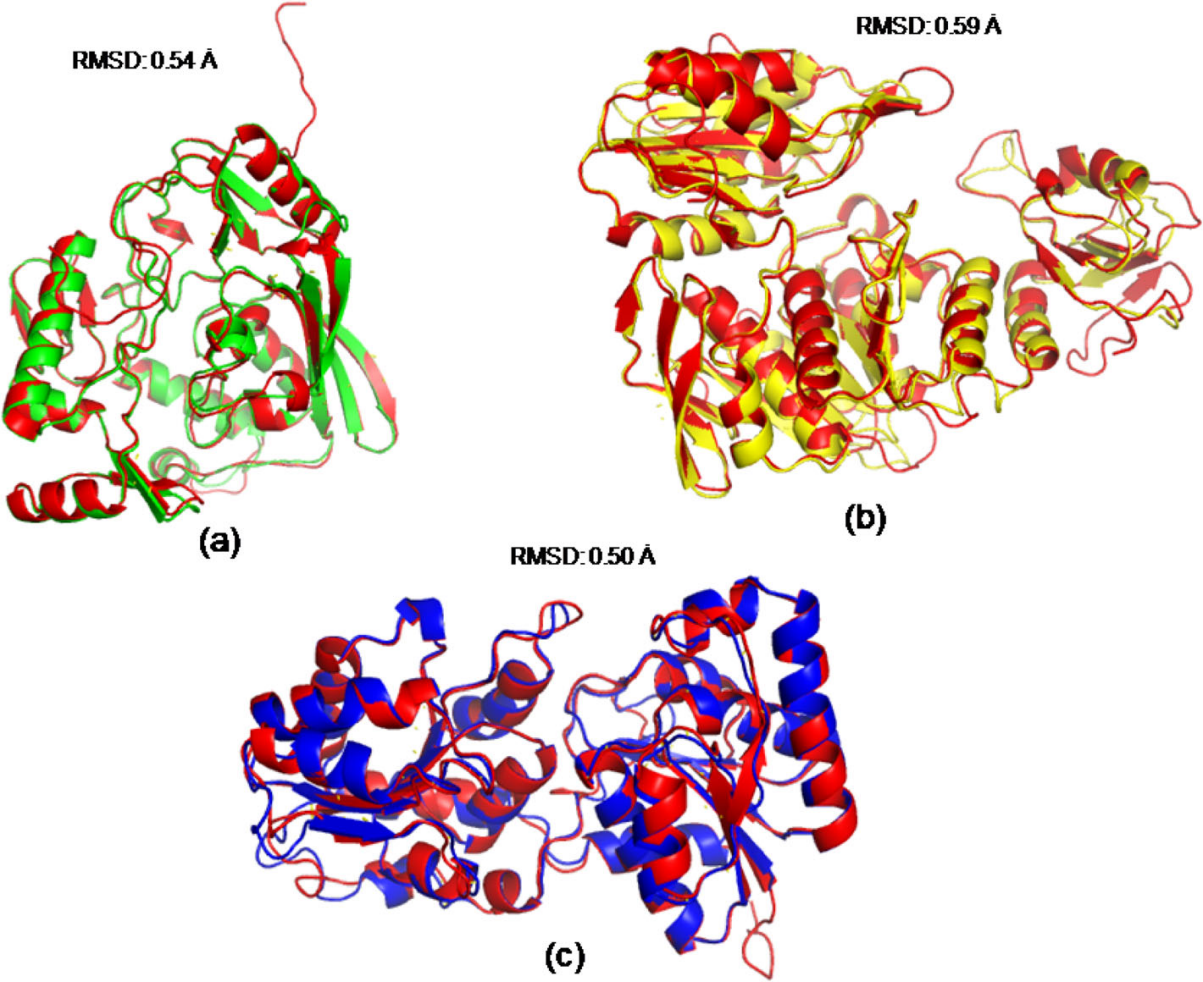
Structural superimposition of **a** MurB from *A*. *baumannii* and *A*. *calcoaceticus*, **b** MurE from *A*. *baumannii* and *A*. *seifertii*, and **c** MurG from *A*. *baumannii* and *A*. *pittii*

The predicted model of three proteins geometrically optimized using YASARA [32] web server and their validation reports were given Fig. S17. The structural quality of MurB from *A. calcoaceticus*, MurE from *A. seifertii*, and MurG from *A. pittii* models were evaluated through stereochemical parameters (Ramachandran plot, Φ–ψ plot and G factor using PROCHECK), Verify_3D and ERRAT evaluating servers. These three servers are available in single web platform named as Structure Analysis and Verification Server (SAVES) (https://servicesn.mbi.ucla.edu/SAVES/). Based on the Ramachandran plot analysis, MurB from *A*. *calcoaceticus* model had 88.6% of all its residues in the most favorable regions and 11.4% were in the additional allowed regions. In the case of ERRAT quality factor, a significant result was obtained with a value of 97.21%. A good and reliable structure has an ERRAT score of 95% and more [35]. This model structure had a Verify_3D validation score of 94.89% and Verify_3D tool cut-off values is greater or equal to 80%. In the same way, the evaluation results of predicted model of MurE from *A*. *seifertii* revealed that 98% of the residues in the most favorable and additionally allowed region in the Ramachandran plot analysis, 91.48% ERRAT quality factor score and 91.51% Verify_3D validation score. Further, the model evaluation results of MurG from *A*. *pittii* indicated that around 99% residues found in the most favorable and additional allowed region in the Ramachandran Map, ERRAT quality factor of 98.25% and Verify_3D score of 86.52%, respectively (Fig. S17). Aforementioned structure validation results explained that predicted models are more reliable and reasonable and they can be used for further studies. Structure superposition studies were performed for MurB, MurE, and MurG from *A*. *baumannii* with MurB from *A*. *calcoaceticus*, MurE from *A*. *seifertii*, and MurG from *A*. *pittii*, respectively. The results obtained from superimposition (Fig. 17) indicated that the predicted models from *A*. *baumannii* and related models from three species are significantly similar with the RMSD of 0.54 (AbMurB with AcMurB), 0.59 (AbMurE with AsMurE), and 0.50 (AbMurG with ApMurG). In addition to this, pairwise structure alignment was also performed for all the models using FATCAT [38], and these results explained that they are significantly similar with the *P* value of 0.00e+00.

## Discussion

The primary protein sequence analysis results (Tables 1 and 2) of Mur family proteins explained that all the proteins were hydrophobic due to the presence of polar and non-polar amino acid residues in their protein sequence (Tables 3 and 6). The hydrophobic nature of the protein indicated that the protein works well in non-polar solvents by crossing the plasma membrane for the formation of the cell wall through the peptidoglycan biosynthetic pathway. The total number of negatively and positively charged amino acid residues of these Mur proteins indicated that most of them are negatively charged, and only two proteins are positively charged, namely MurG and MraY. Both pI and the number of charged amino acids results indicated that most of the enzyme might optimally active in the acidic environment. But, MurG and MraY may be active in a basic environment. This result may be beneficial for developing buffer systems for purification of proteins in the near future by isoelectric focusing (IEF) and two-dimensional poly acrylamide gel electrophoresis (2D-PAGE) [39]. The instability index (II) of Mur family proteins indicated that all Mur proteins are thermodynamically stable. If the protein is unstable, the II is greater than 40. In this case, the high EC of MurB, MurE, and MraY indicates the presence of a high level of Cys, Trp, and Tyr. On the other hand, in the case of MurC and MurA, low number of these amino acid residues even no Trp in respect to MurC. The calculated EC for the Mur family protein revealed that all of them could be studied by UV spectral method. The computed protein concentration and extinction coefficients help in the quantitative study of protein-protein and protein-ligand interactions in solution [40].

**Table 6:** Sequence identity statistics of eight Mur proteins from *A*. *baumannii*

S/No.	MurA	MurB	MurC	MurD	MurE	MurF	MraY	MurG
1	MurA	12.7479	11.4833	11.7225	11.0048	12.4402	11.0215	11.7808
2	MurB		12.1813	11.0482	12.4646	11.898	12.1813	11.0482
3	MurC			14.7253	12.0332	15.4506	11.0215	12.6027
4	MurD				13.4066	14.5055	13.7097	13.1507
5	MurE					15.4506	11.2903	12.0548
6	MurF						11.5591	11.5068
7	MraY							11.2329
8	MurG							

The conserved sequence motifs found in the four Mur enzymes also map to other members of the Mur ligase family [11]. The Mur family of enzymes have several antigenic sites, which will additionally be supported that predicted binding sites are critical and consider as putative active site region for docking and virtual screening against control and library of natural molecules in the ZINC database [41]. Moreover, the predicted antigenic sites (or binding sites) are mostly conserved in a homologous sequence of MurB, MurE, MraY, MurG, MurD, MurF, MurC, and MurA. Together, the results obtained from the protein sequence analysis of eight Mur family protein were divided into three main categories, namely transferases (MurG, MurA, and MraY), ligases (MurC, MurD, MurE, and MurG), and oxidoreductase enzymes (MurB) (Table 2). The biological process of these enzymes is cell division, regulation of cell shape, cell cycle, and the cell wall organization. They are using the same pathway known as peptidoglycan biosynthesis, which is a critical role for the formation of cell wall in prokaryotic organisms. The four (MurC, MurD, MurE, and MurF) Mur ligases are responsible for the successive additions of l-alanine, d-glutamate, meso-diaminopimelate or l-lysine, and d-alanyl-d-alanine to UDP-*N*-acetylmuramic acid in the peptidoglycan pathways.

A recent study explained that post-translational modification (PTM) processes are very important for pathogenicity, virulence, and drug-resistance nature of ESKAPE microorganism [42]. Understanding these modifications and host proteins manipulated by these processes facilitate to examine host-pathogen interactions and help to design the more potent molecules against ESKAPE microorganism. Based on the previous reports, MDRAB might also undergo several PTMs, particularly phosphorylation [42], glycosylation [42], and acetylation [42]. From the post-translational modification results, the residues participated in PTM might be involved in interactions with various ligands and drug molecules, and this will additionally be supported that these residues are worth for further investigation. The results of intra comparative analysis revealed that MurC, MurD, MurE, and MurF belong to ligases; MurB belongs to oxidoreductase; and MurA, MraY, and MurG belong to transferases. Based on the overall mean average, Mur family protein are slightly divergent. The existing report indicated that the evolutionary relationship of antifreeze proteins showed that 1.589 was an overall mean value with significant conserved sites [39].

**Fig. 18:**
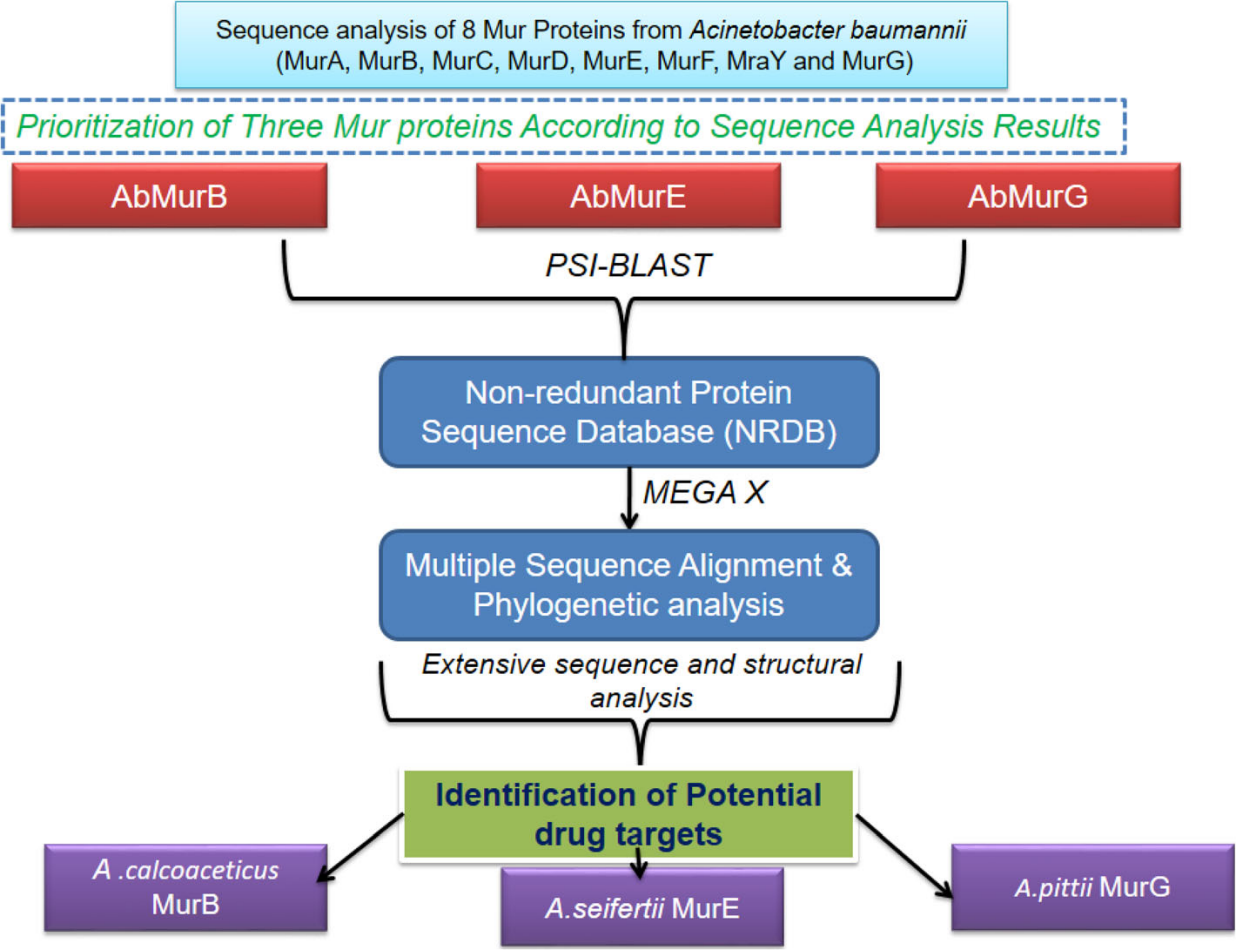
Overall workflow for the prioritization of Mur family proteins from *A*. *baumannii* and identification of their homologous proteins in other species

The results obtained from protein sequence analysis, inter-phylogenetic analysis, and secondary and 3D structure predictions, we identified potential drug targets from *A*. *calcoaceticus* (MurB), *A*. *seifertii* (MurE), and *A*. *pittii* (MurG) which are very similar to existing drug targets (MurB, MurE, and MurG found in *A*. *baumannii*) as indicated in the overall workflow (Fig. 18). Active site region of the reported as well as predicted models were also very similar with each other. Moreover, these results open a new therapeutic route for treating the two or more bacterial diseases using a single potent molecule.

## Conclusion

In silico-based screening of potential bacterial drug targets was identified in the present study with the aid of systematic computational workflow. Initially, the proteins that participated in the peptidoglycan pathways are MurA, MurB, MurC, MurD, MurE, MurF, MraY, and MurG which were retrieved from the UniProt database. After this, we performed primary sequence analysis, multiple sequence alignment, and phylogenetic tree construction and obtained results allowed us to classify the Mur proteins into three main enzymatic groups based on sequential properties. The sequence of each selected Mur protein was submitted into PSI-BLAST against NRDB to identify the homologous sequences. Based on the multiple sequence alignment, molecular phylogeny, and the pairwise sequence alignment results, we identified potential drug targets, namely MurB from *A*. *calcoaceticus*, MurE from *A*. *seifertii*, and MurG from *A*. *pittii*. The structural and functional similarity and identity of newly identified drug target proteins are validated using primary sequence analysis, consensus secondary structure prediction, and structural superimposition. This proposed methodology can also be used to identify and prioritize the drug targets in other bacteria which causes various diseases. This opens a new route for further computational and experimental studies to identify the common antibacterial molecules which may act on multi-targeted proteins from many bacteria.

## Supplementary information

**Additional file 1: **Phylogenetic analysis: **Figure S1.** Phylogenetic analysis of MurB Protein using UPGMA Method. **Figure S2.** Phylogenetic analysis of MurB using Neighbor Joining Method. **Figure S3.** Phylogenetic analysis of MurB using Maximum Parsimony Method. **Figure S4.** Phylogenetic analysis of MurB using Minimum Evolution Method. **Figure S5.** Phylogenetic analysis of MurB using Maximum Likelihood Method. **Figure S6.** Phylogenetic analysis of MurE using UPGMA Method. **Figure S7.** Phylogenetic analysis of MurE using Neighbor Joining Method. **Figure S8.** Phylogenetic analysis of MurE using Minimum Evolution. **Figure S9.** Phylogentic analysis of MurE using Maximum Parsimony method. **Figure S10.** Phylogenetic analysis of MurE using Maximum Likelihood Method. **Figure S11.** Phylogenetic analysis of MurG using UPGMA Method. **Figure S12.** Phylogenetic Analysis of MurG using Neighbor Joining Method. **Figure S13.** Phylogenetic analysis of MurG using Minimum Evolution Method. **Figure S14.** Phylogenetic analysis of MurG using Maximum Parsimony Method. **Figure S15.** Phylogenetic analysis of MurG using Maximum Likelihood Method. **Figure S16.** Multiple Sequence Alignment Eight Mur Family Proteins from *Acinetobacter baumannii.***Figure S17.** Structure validation reports for MurB, MurE and MurG from different species

## Data Availability

All data generated or analyzed during this study are included in this manuscript.

## Abbreviations

Ac: *A*. *calcoaceticus*
As: *A*. *seifertii*
Ap: *A*. *pittii*
Ab: *A*. *baumannii*
CSSP: Consensus Secondary Structure Prediction
MSA: Multiple sequence alignment
RMSD: Root mean square deviation
UPGMA: Unweighted pair group method with arithmetic mean
MP: Maximum parsimony
ME: Minimum evolution
NJ: Neighbor joining
ML: Maximum likelihood

## References

[1] Almasaudi SB (2018). Acinetobacter spp. as nosocomial pathogens: EPIDEMIOLOGY and resistance features. Saudi J Biol Sci.

[2] Peleg AY, Seifert H, Paterson DL (2008). Acinetobacter baumannii: emergence of a successful pathogen. Clin Microbiol Rev.

[3] Doughari HJ, Ndakidemi PA, Human IS, Benade S (2009) The ecology, biology and pathogenesis of Acinetobacter spp.: an overview . Microbes Environ 1103150282-1103150282 10.1264/jsme2.me10179 21502736

[4] Wong D, Nielsen TB, Bonomo RA, Pantapalangkoor P, Luna B, Spellberg B (2017). Clinical and Pathophysiological Overview of Acinetobacter Infections: a Century of Challenges. Clin Microbiol Rev.

[5] Durante-Mangoni E, Utili R, Zarrilli R (2014). Combination therapy in severe Acinetobacter baumannii infections: an update on the evidence to date. Future Microbiol.

[6] Shrivastava SR, Shrivastava PS, Ramasamy J (2018). World health organization releases global priority list of antibiotic-resistant bacteria to guide research, discovery, and development of new antibiotics. J Med Soc.

[7] Amera GM, Khan RJ, Pathak A, Kumar A, Singh AK (2019) Structure based in-silico study on UDP-N-acetylmuramoyl-L-alanyl-D-glutamate-2,6-diaminopimelate ligase (MurE) from Acinetobacter baumannii as a drug target against nosocomial infections. Informatics in Medicine Unlocked 16. doi:10.1016/j.imu.2019.100216

[8] Moraes GL, Gomes GC, Monteiro de Sousa PR, Alves CN, Govender T, Kruger HG, Maguire GE, Lamichhane G, Lameira J (2015). Structural and functional features of enzymes of Mycobacterium tuberculosis peptidoglycan biosynthesis as targets for drug development. Tuberculosis (Edinb).

[9] Benson TE, Walsh CT, Hogle JM (1996). The structure of the substrate-free form of MurB, an essential enzyme for the synthesis of bacterial cell walls. Structure.

[10] Gordon E, Flouret B, Chantalat L, van Heijenoort J, Mengin-Lecreulx D, Dideberg O (2001). Crystal structure of UDP-N-acetylmuramoyl-L-alanyl-D-glutamate: meso-diaminopimelate ligase from Escherichia coli. J Biol Chem.

[11] Ziegler K, Diener A, Herpin C, Richter R, Deutzmann R, Lockau W (1998). Molecular characterization of cyanophycin synthetase, the enzyme catalyzing the biosynthesis of the cyanobacterial reserve material multi-L-arginyl-poly-L-aspartate (cyanophycin). Eur J Biochem.

[12] Al-Dabbagh B, Henry X, Ghachi ME, Auger G, Blanot D, Parquet C, Mengin-Lecreulx D, Bouhss A (2008). Active site mapping of MraY, a member of the polyprenyl-phosphate N-acetylhexosamine 1-phosphate transferase superfamily, catalyzing the first membrane step of peptidoglycan biosynthesis. Biochemistry.

[13] Fakhar Z, Naiker S, Alves CN, Govender T, Maguire GE, Lameira J, Lamichhane G, Kruger HG, Honarparvar B (2016). A comparative modeling and molecular docking study on Mycobacterium tuberculosis targets involved in peptidoglycan biosynthesis. J Biomol Struct Dyn.

[14] Amera GM, Khan RJ, Pathak A, Jha RK, Muthukumaran J, Singh AK (2020). Computer aided ligand based screening for identification of promising molecules against enzymes involved in peptidoglycan biosynthetic pathway from Acinetobacter baumannii. Microbial Pathogenesis.

[15] Sosa EJ, Burguener G, Lanzarotti E, Defelipe L, Radusky L, Pardo AM, Marti M, Turjanski AG, Fernández Do Porto D (2018). Target-pathogen: a structural bioinformatic approach to prioritize drug targets in pathogens. Nucleic Acids Res.

[16] Ramos PIP, Fernández Do Porto D, Lanzarotti E, Sosa EJ, Burguener G, Pardo AM, Klein CC, Sagot MF, de Vasconcelos ATR, Gales AC, Marti M, Turjanski AG, Nicolás MF (2018). An integrative, multi-omics approach towards the prioritization of Klebsiella pneumoniae drug targets. Scientific reports.

[17] Hossain T, Kamruzzaman M, Choudhury TZ, Mahmood HN, Nabi A, Hosen MI (2017) Application of the subtractive genomics and molecular docking analysis for the identification of novel putative drug targets against Salmonella enterica subsp. enterica serovar Poona. BioMed Res Int 2017:3783714 doi:10.1155/2017/3783714 10.1155/2017/3783714 PMC5585685 28904956

[18] Uddin R, Jamil F (2018). Prioritization of potential drug targets against P. aeruginosa by core proteomic analysis using computational subtractive genomics and protein-protein interaction network. Comput Biol Chem.

[19] Gao Z, Li H, Zhang H, Liu X, Kang L, Luo X, Zhu W, Chen K, Wang X, Jiang H (2008). PDTD: a web-accessible protein database for drug target identification. BMC Bioinformatics.

[20] Gasteiger E, Hoogland C, Gattiker A, Wilkins MR, Appel RD, Bairoch A (2005) Protein identification and analysis tools on the ExPASy server. In: The proteomics protocols handbook. Springer, pp 571-607

[21] Letunic I, Doerks T, Bork P (2014). SMART: recent updates, new developments and status in 2015. Nucleic Acids Res.

[22] Yu NY, Wagner JR, Laird MR, Melli G, Rey S, Lo R, Dao P, Sahinalp SC, Ester M, Foster L (2010). PSORTb 3.0: improved protein subcellular localization prediction with refined localization subcategories and predictive capabilities for all prokaryotes. Bioinformatics.

[23] Rice P, Longden I, Bleasby A (2000) EMBOSS: the European molecular biology open software suite. Elsevier current trends, 10.1016/s0168-9525(00)02024-2 10827456

[24] Kurata H, Sugimoto Y (2018). Improved kinetic model of Escherichia coli central carbon metabolism in batch and continuous cultures. J Biosci Bioeng.

[25] Bhat AH, Mondal H, Chauhan JS, Raghava GP, Methi A, Rao A (2012). ProGlycProt: a repository of experimentally characterized prokaryotic glycoproteins. Nucleic Acids Res.

[26] Deng W, Wang C, Zhang Y, Xu Y, Zhang S, Liu Z, Xue Y (2016). GPS-PAIL: prediction of lysine acetyltransferase-specific modification sites from protein sequences. Scientific reports.

[27] Kumar S, Stecher G, Li M, Knyaz C, Tamura K (2018). MEGA X: molecular evolutionary genetics analysis across computing platforms. Mol Biol Evol.

[28] Altschul SF, Madden TL, Schaffer AA, Zhang J, Zhang Z, Miller W, Lipman DJ (1997). Gapped BLAST and PSI-BLAST: a new generation of protein database search programs. Nucleic acids research.

[29] Gupta A, Deshpande A, Amburi JK, Sabarinathan R, Senthilkumar R, Sekar K (2009). CSSP (Consensus Secondary Structure Prediction): a web-based server for structural biologists. Journal of Applied Crystallography.

[30] Amera GM, Khan RJ, Pathak A, Jha RK, Muthukumaran J, Singh AK (2019) Screening of Promising molecules against MurG as drug target in multi-drug-resistant-acinetobacter baumannii—insights from comparative protein modeling, molecular docking and molecular dynamics simulation. J Biomol Struct Dyn:1–37. 10.1080/07391102.2019.1700167 10.1080/07391102.2019.1700167 31787065

[31] Waterhouse A, Bertoni M, Bienert S, Studer G, Tauriello G, Gumienny R, Heer FT, de Beer TA P, Rempfer C, Bordoli L, Lepore R, Schwede T (2018) SWISS-MODEL: homology modelling of protein structures and complexes. Nucleic acids research 46 (W1):W296-W303. doi:10.1093/nar/gky427 %J Nucleic Acids Research 10.1093/nar/gky427 PMC6030848 29788355

[32] Krieger E, Joo K, Lee J, Lee J, Raman S, Thompson J, Tyka M, Baker D, Karplus K (2009). Improving physical realism, stereochemistry, and side-chain accuracy in homology modeling: four approaches that performed well in CASP8. Proteins.

[33] Luthy R, Bowie JU, Eisenberg D (1992). Assessment of protein models with three-dimensional profiles. Nature.

[34] Laskowski RA, Rullmann JAC, MacArthur MW, Kaptein R, Thornton JM (1996). AQUA and PROCHECK-NMR: programs for checking the quality of protein structures solved by NMR. J Biomol NMR.

[35] Colovos C, Yeates TO (1993). Verification of protein structures: patterns of nonbonded atomic interactions. Protein.

[36] Pontius J, Richelle J, Wodak SJ (1996). Deviations from standard atomic volumes as a quality measure for protein crystal structures. J Mol Biol.

[37] DeLano WL (2002) The PyMOL molecular graphics system. http://www pymol org

[38] Li Z, Ye Y, Godzik A (2006) Flexible Structural Neighborhood--a database of protein structural similarities and alignments. Nucleic Acids Res 34 (Database issue):D277-D280. doi:10.1093/nar/gkj124 10.1093/nar/gkj124 PMC1347486 16381864

[39] Muthukumaran J, Manivel P, Kannan M, Jeyakanthan J, Krishna R (2011). A framework for classification of antifreeze proteins in over wintering plants based on their sequence and structural features. J Bioinformat Sequence Anal.

[40] Sivakumar K, Balaji S (2007). In silico characterization of antifreeze proteins using computational tools and servers. Journal of Chemical Sciences.

[41] Sterling T, Irwin JJ (2015). ZINC 15—ligand discovery for everyone. J Chem Inform Modeling.

[42] Tiwari V (2019). Post-translational modification of ESKAPE pathogens as a potential target in drug discovery. Drug discovery today.

