# Supplementary material for: Prioritization of Mur family drug targets against A. baumannii and identification of their homologous proteins through molecular phylogeny, primary sequence, and structural analysis
Source: J Genet Eng Biotechnol. 2020 Jul 28;18:33. doi: 10.1186/s43141-020-00048-4 (PMC7387395; doi:10.1186/s43141-020-00048-4)

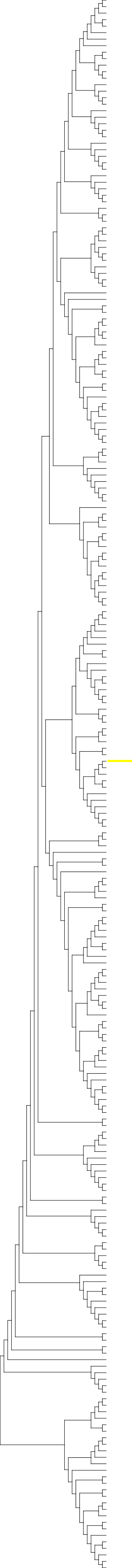


**Fig. S1.** Phylogenetic analysis of MurB Protein using UPGMA Method

99

94

35

14

5

1

18

27

99

80

6

99

17

15

99

99

79

82

99

15

32

39

42

60

65

55

12

56

14

15

29

9

19

62

99

99

99

64

37

24

24

18

67

42

95

99

99

39

99

35

46

99

50

69

79

99

99

99

99

91

99

84

81

61

70

87

75

99

53

48

99

99 29

41

49

99

64

99

50

99

98

81

99

43

99

56

50 99

87

43

95

45

91

56

55

54

99

24

10

99

99

32

58

93

99

99

7

99

40

52

60

99

27

99

96 67

40

99

54

26

23

39

95

43

70

31

68

69

62

92

40

34

56

96

58

99

98

35

42

42

99 50

65

19

27

27

40

40

63

78

99

99

77

50

99

99

47

43

67

99

97

60

91

58

62

60

99

77

46

55

99

99

99

61

99

71

78

94

93

35

89

83

30

33

99

54

99

88

82

99

87

99

66

81

98

24

99

43

54

99

22

87

99

9

64

99

99

61

69

46

40

99

20

99

99

99

99

99

99

97

21

99

77

93

99

72

95

23

96

42

8

32

16

27

85

31

82

82

66

RDC53282.1

WP 121979773.1

WP 005263277.1

WP 044108858.1

WP 005252175.1

WP 005106607.1

WP 071852098.1

WP 085064860.1

WP 100535294.1

WP 100357931.1

WP 100329600.1 ENW23442.1 WP 005170480.1

WP 034170542.1

WP 104425984.1

WP 004783906.1

WP 125278995.1

WP 067730133.1

WP 034589112.1

WP 099338924.1

WP 076753712.1

WP 048881277.1

WP 086177911.1

WP 067767348.1

WP 078188596.1

WP 078193980.1

WP 111884273.1

WP 068888366.1

WP 068912455.1

WP 086214022.1

WP 005009136.1

WP 067668051.1

WP 120402692.1

WP 106984903.1

WP 117007599.1 PZQ93846.1 OHC25505.1 WP 078388853.1

WP 078425051.1

WP 058869704.1

WP 062032708.1

WP 087527163.1

WP 086210090.1

WP 119063450.1

WP 110973970.1

WP 104494655.1

WP 039622605.1

WP 092719028.1

WP 053578031.1

WP 004648812.1

WP 086165846.1

WP 092688991.1

WP 086184455.1

WP 086187897.1

WP 092818312.1

WP 131274545.1

WP 086195433.1

WP 086175029.1

WP 086200014.1

WP 131363680.1

WP 067866885.1

WP 131216170.1

WP 131382805.1

WP 131269383.1

WP 131316571.1

WP 131271973.1

WP 131291988.1

WP 086192676.1

WP 067726144.1

WP 131260922.1

WP 104852096.1

WP 122897868.1

WP 142768629.1

WP 131326324.1

WP 131264387.1

WP 131321346.1

WP 044740426.1

WP 116760892.1

WP 004866062.1

WP 081406150.1

WP 111859169.1

WP 107009966.1

WP 086206045.1

WP 086203857.1

WP 086190958.1

WP 130114153.1

WP 130132460.1

WP 130116275.1

WP 139422253.1

WP 046761168.1

WP 096734361.1

WP 120384109.1

WP 089604337.1

WP 010115534.1 EEY78511.1

WP 092706965.1

WP 056480822.1

WP 133746817.1

WP 132069363.1

WP 009390302.1

WP 133972960.1

WP 042893972.1

WP 057105832.1

WP 032038131.1

WP 032005954.1

WP 060466592.1

WP 086266293.1

WP 033847372.1 EFF86130.1 RJE57869.1 HBU88774.1

WP 005306417.1 HCD61322.1

WP 106465314.1

WP 086496969.1 SSQ03959.1

WP 001166916.1

MurB A.baumannii EMT98785.1

WP 001166920.1

WP 001166921.1 B0VRK7.1 EEX04534.1 ETY69091.1 OTL38132.1 EGJ67433.1 ETQ95341.1

WP 114327793.1 HAK16169.1 EEY93828.1

WP 129262666.1 TXJ03386.1

WP 100833701.1

WP 150026059.1

WP 005061967.1

WP 004777230.1

WP 004757822.1

WP 004679755.1

WP 004761426.1

WP 023013599.1

WP 061525088.1

WP 062846995.1

WP 069577941.1

WP 004771880.1

WP 047426963.1

WP 123774487.1

WP 005257213.1

WP 005230051.1

WP 038344308.1

WP 087547790.1

WP 087536163.1

WP 087545799.1

WP 005160447.1

WP 046739560.1

WP 005215963.1

WP 004661949.1

WP 018678197.1

WP 130161378.1

WP 121929248.1

WP 004670863.1

WP 005196737.1 OJU81966.1

WP 004653193.1

WP 070075443.1

WP 065342727.1

WP 005293537.1

WP 005152500.1

WP 005203267.1

WP 004807589.1

WP 005189129.1

WP 016163488.1

WP 005212001.1

WP 087512378.1

WP 087550938.1 APV37518.1

WP 081408837.1 KOR11649.1

WP 004927209.1

WP 130804732.1

WP 120369242.1 HCK31656.1

WP 080776124.1 EXD37990.1 ENV79435.1

WP 054582605.1

WP 086181081.1

WP 121972832.1 PKH33068.1

WP 081374976.1 EEY87139.1

WP 068791239.1 AMW80367.1 WP 081406007.1

WP 139852256.1

WP 016656816.1

WP 042111707.1

WP 088822514.1

WP 111894322.1

WP 131378391.1

WP 131314674.1

WP 045793084.1

WP 131389917.1

WP 004752377.1

WP 131381559.1

WP 116726100.1

WP 092767856.1

WP 035266127.1

WP 092747017.1

WP 023271922.1

WP 067553711.1

WP 092621655.1

WP 070069158.1

WP 097078384.1 OUY07874.1

WP 087620029.1

WP 134243271.1

WP 076876706.1 RZA05516.1

WP 114898979.1

WP 022967536.1

WP 151183966.1

WP 137403266.1

WP 141615335.1

WP 015266767.1

WP 112785906.1

WP 143961510.1

WP 067547210.1

WP 087939839.1

WP 092789489.1

WP 026951572.1

WP 135071065.1

WP 074224298.1

WP 008200601.1

WP 111317580.1

WP 133553205.1 TNF39299.1

WP 111392935.1

WP 103923541.1

WP 111669820.1

WP 092731536.1

WP 077351401.1

WP 119478405.1

|  | |  | 28 | WP 005189129.1 |
| --- | --- | --- | --- | --- |
|  |  | 34 |  | WP 005212001.1 |
|  | 32 |  |  | WP 016163488.1 |
| 93 |  |  |  | WP 005203267.1 |
|  |  |  | 31 | WP 004807589.1 |
|  |  |  |  | WP 005152500.1 |
|  |  |  |  | WP 005293537.1 |
|  |  |  |  | WP 065342727.1 |
|  | 83 |  |  | OJU81966.1 |
|  |  | 99 |  | WP 070075443.1 |
|  |  |  | 55 | WP 004653193.1 |
|  |  |  |  | WP 018678197.1 |
|  |  |  | 72 | WP 046739560.1 |
|  |  | 64 |  | WP 004661949.1 |
|  |  |  |  | WP 005215963.1 |
| 99 |  |  |  | WP 005160447.1 |
|  | 71 |  |  | WP 087545799.1 |
|  |  | 43 |  | WP 087547790.1 |
|  |  |  | 54 | WP 087536163.1 |
|  |  |  | 99 | WP 130161378.1 |
|  |  |  |  | WP 121929248.1 |
|  |  | 99 |  | WP 004670863.1 |
|  |  |  | 99 | WP 005196737.1 |
|  |  |  |  | WP 038344308.1 |
|  |  |  |  | WP 005230051.1 |
|  |  |  |  | WP 047426963.1 |
|  |  |  | 79 | WP 123774487.1 |
|  |  |  |  |  |
| 99 |  |  |  | WP 005257213.1 |
|  | 63 |  |  | WP 062846995.1 |
|  |  | 74 |  | WP 004771880.1 |
|  |  |  | 55 | WP 069577941.1 |
|  |  |  |  | WP 023013599.1 |
|  |  |  | 99 | WP 061525088.1 |
|  |  |  |  | WP 005061967.1 |
| 18 |  |  |  | WP 004761426.1 |
|  | 99 |  |  | WP 004679755.1 |
|  |  | 31 |  | WP 004777230.1 |
|  |  |  | 42 | WP 004757822.1 |
|  |  |  |  | WP 100833701.1 |
|  |  |  | 99 | WP 150026059.1 |
|  |  |  |  | TXJ03386.1 |
|  | 32 |  |  | WP 129262666.1 |
|  |  | 99 |  | HAK16169.1 |
|  |  |  | 83 | EEY93828.1 |
|  |  |  | 71 | OTL38132.1 |
|  |  | 40 |  | ETQ95341.1 |
|  | 30 |  |  | ETY69091.1 |
| 26 |  |  |  | EGJ67433.1 |
|  |  |  |  | WP 114327793.1 |
|  |  |  |  | EEX04534.1 |
|  |  |  |  | WP 001166916.1 |
|  |  |  | 53 | WP 001166921.1 |
|  |  |  |  | B0VRK7.1 |
|  |  | 38 |  | WP 001166920.1 |
|  |  |  |  |  |
|  |  |  | 15 | MurB A.baumannii |
|  |  |  |  | EMT98785.1 |
|  |  |  |  | SSQ03959.1 |
|  |  |  | 99 | HCD61322.1 |
|  |  | 99 |  | WP 106465314.1 |
|  |  |  |  | WP 086496969.1 |
|  |  |  | 58 | HBU88774.1 |
|  |  | 89 |  | WP 005306417.1 |
|  |  |  |  | RJE57869.1 |
|  |  |  | 48 | WP 032005954.1 |
|  |  | 47 |  | WP 060466592.1 |
|  | 41 |  |  | WP 032038131.1 |
| 93 |  |  |  | WP 033847372.1 |
|  |  |  | 78 | EFF86130.1 |
|  |  |  |  | WP 086266293.1 |
|  |  |  |  | WP 057105832.1 |
|  |  |  | 47 | WP 133972960.1 |
|  |  |  |  | WP 042893972.1 |
|  |  |  |  | WP 009390302.1 |
|  |  |  | 37 | WP 132069363.1 |
|  |  |  |  |  |
| 59 |  |  |  | WP 056480822.1 |
|  | 92 |  |  | WP 092706965.1 |
|  |  | 32 |  | EEY78511.1 |
|  |  |  | 44 | WP 133746817.1 |
|  |  |  | 63 | APV37518.1 |
|  |  | 99 |  | WP 081408837.1 |
|  | 83 |  |  | KOR11649.1 |
|  |  |  |  | WP 004927209.1 |
|  |  |  |  | WP 130804732.1 |
|  |  |  |  | WP 120369242.1 |
| 67 |  |  |  | HCK31656.1 |
|  | 99 |  |  | WP 080776124.1 |
|  |  | 65 |  | EXD37990.1 |
|  |  |  | 88 | ENV79435.1 |
|  |  |  | 99 | WP 092767856.1 |
|  |  |  |  | WP 035266127.1 |
|  |  |  | 36 | WP 004752377.1 |
|  |  | 57 |  | WP 131389917.1 |
|  | 79 |  |  | WP 131381559.1 |
| 40 |  |  |  | WP 116726100.1 |
|  |  |  |  | WP 045793084.1 |
|  |  |  |  | WP 131378391.1 |
|  |  |  | 59 | WP 131314674.1 |
|  |  |  |  | WP 111894322.1 |
|  |  |  |  | WP 088822514.1 |
|  |  | 44 |  | WP 092747017.1 |
|  |  |  | 46 | WP 023271922.1 |
|  |  |  | 99 | OUY07874.1 |
|  |  | 99 |  | WP 087620029.1 |
|  | 89 |  |  | WP 097078384.1 |
| 99 |  |  |  | WP 070069158.1 |
|  |  |  |  | WP 092621655.1 |
|  |  |  |  | WP 067553711.1 |
|  |  |  | 99 | WP 134243271.1 |
|  |  | 99 |  | WP 076876706.1 |
|  | 62 |  |  | RZA05516.1 |
| 27 |  |  |  | WP 114898979.1 |
|  |  |  |  | WP 022967536.1 |
|  |  |  | 99 | WP 151183966.1 |
|  |  |  | 65 | WP 137403266.1 |
|  |  | 64 |  | WP 141615335.1 |
|  | 99 |  |  | WP 015266767.1 |
| 67 |  |  |  | WP 112785906.1 |
|  |  |  |  | WP 143961510.1 |
|  |  |  |  | WP 067547210.1 |
|  |  |  |  | WP 026951572.1 |
|  |  |  | 89 | WP 087939839.1 |
|  |  | 21 |  | WP 092789489.1 |
|  |  |  |  | WP 135071065.1 |
|  |  |  | 88 | WP 074224298.1 |
|  |  | 51 |  | WP 008200601.1 |
|  |  |  |  | WP 111317580.1 |
|  |  |  | 30 | WP 133553205.1 |
|  |  |  |  | TNF39299.1 |
|  |  |  |  | WP 111392935.1 |
|  |  |  | 81 | WP 103923541.1 |
|  |  |  |  |  |
| 11 |  |  |  | WP 111669820.1 |
|  | 73 |  |  | WP 092731536.1 |
|  |  | 74 |  | WP 077351401.1 |
|  |  |  | 68 | WP 119478405.1 |
|  |  |  | 74 | PZQ93846.1 |
|  |  | 47 |  | OHC25505.1 |
|  | 99 |  |  | WP 078388853.1 |
|  |  |  |  | WP 058869704.1 |
| 65 |  | 41 |  | WP 062032708.1 |
|  |  |  | 42 | WP 078425051.1 |
|  |  |  |  | WP 087527163.1 |
|  |  |  |  | WP 119063450.1 |
|  |  | 99 |  | WP 086210090.1 |
|  |  |  | 87 | WP 110973970.1 |
|  |  |  |  | AMW80367.1 |
|  |  |  | 99 | WP 081406007.1 |
|  |  |  |  | WP 139852256.1 |
|  |  | 67 |  | WP 016656816.1 |
|  |  |  | 99 | WP 042111707.1 |
|  |  |  | 99 | WP 081406150.1 |
|  |  | 99 |  | WP 111859169.1 |
|  | 67 |  |  | WP 107009966.1 |
|  |  |  |  | WP 086190958.1 |
|  |  | 48 |  | WP 086206045.1 |
|  |  |  | 99 | WP 086203857.1 |
|  |  |  | 63 | WP 130114153.1 |
|  |  | 99 |  | WP 130132460.1 |
|  |  |  |  | WP 130116275.1 |
|  |  |  | 99 | WP 139422253.1 |
| 98 |  | 75 |  | WP 046761168.1 |
|  |  |  |  | WP 096734361.1 |
|  | 55 |  |  | WP 120384109.1 |
|  |  | 99 |  | WP 089604337.1 |
|  |  |  | 66 | WP 010115534.1 |
|  |  |  | 60 | EEY87139.1 |
|  |  | 76 |  | WP 068791239.1 |
|  | 99 |  |  | WP 081374976.1 |
| 39 |  |  |  | PKH33068.1 |
|  |  |  |  | WP 121972832.1 |
|  |  |  |  | WP 004866062.1 |
|  |  | 20 |  | WP 054582605.1 |
|  |  |  | 99 | WP 086181081.1 |
|  |  |  | 73 | WP 086192676.1 |
|  |  | 65 |  | WP 067726144.1 |
|  | 64 |  |  | WP 131291988.1 |
| 83 |  |  |  | WP 131271973.1 |
|  |  |  |  | WP 131269383.1 |
|  |  | 98 |  | WP 131382805.1 |
|  |  |  | 46 | WP 131316571.1 |
|  |  |  |  | WP 131216170.1 |
|  |  | 55 |  | WP 131363680.1 |
|  |  |  | 99 | WP 067866885.1 |
|  |  |  |  | WP 086187897.1 |
|  |  |  | 54 | WP 004648812.1 |
|  |  |  |  |  |
|  | 99 |  |  | WP 086165846.1 |
|  |  | 99 |  | WP 092688991.1 |
|  |  |  | 70 | WP 086184455.1 |
|  |  |  | 98 | WP 092818312.1 |
|  |  |  |  | WP 131274545.1 |
|  | 99 |  |  | WP 086195433.1 |
|  |  | 52 |  | WP 086175029.1 |
|  |  |  | 60 | WP 086200014.1 |
|  |  |  |  | WP 092719028.1 |
|  |  |  | 99 | WP 053578031.1 |
|  |  |  |  | WP 039622605.1 |
|  |  |  |  | WP 104494655.1 |
|  |  |  | 99 | WP 087512378.1 |
|  |  |  |  | WP 087550938.1 |
|  |  |  | 96 | WP 044740426.1 |
|  |  | 64 |  | WP 116760892.1 |
|  | 41 |  |  | WP 131321346.1 |
| 99 |  |  |  | WP 131264387.1 |
|  |  |  |  | WP 131326324.1 |
|  |  |  |  | WP 142768629.1 |
|  |  |  |  | WP 122897868.1 |
|  |  | 64 |  | WP 131260922.1 |
|  |  |  | 99 | WP 104852096.1 |
|  |  |  | 78 | WP 005009136.1 |
|  |  | 93 |  | WP 067668051.1 |
|  | 96 |  |  | WP 086214022.1 |
|  |  |  |  | WP 068888366.1 |
| 45 |  |  | 71 | WP 068912455.1 |
|  |  |  |  | WP 120402692.1 |
|  |  | 87 |  | WP 106984903.1 |
|  |  |  | 99 | WP 117007599.1 |
|  |  |  | 90 | WP 078193980.1 |
|  |  | 99 |  | WP 111884273.1 |
|  | 87 |  |  | WP 078188596.1 |
| 99 |  |  |  | WP 067767348.1 |
|  |  |  |  | WP 086177911.1 |
|  |  |  | 99 | WP 076753712.1 |
|  |  | 99 |  | WP 048881277.1 |
|  | 99 |  |  | WP 099338924.1 |
| 81 |  |  |  | WP 034589112.1 |
|  |  |  |  | WP 067730133.1 |
|  |  |  | 89 | WP 004783906.1 |
|  |  | 65 |  | WP 125278995.1 |
|  | 99 |  |  | WP 104425984.1 |
|  |  |  |  | WP 034170542.1 |
|  |  |  | 51 | ENW23442.1 |
|  |  | 44 |  | WP 100329600.1 |
|  | 99 |  |  | WP 005170480.1 |
|  |  |  |  | WP 100535294.1 |
|  |  |  | 69 | WP 100357931.1 |
|  |  |  |  | WP 085064860.1 |
|  |  |  |  | WP 071852098.1 |
|  |  |  | 38 | WP 044108858.1 |
|  |  |  |  | WP 005252175.1 |
| 22 |  |  |  | WP 005106607.1 |
|  | 12 |  |  | WP 005263277.1 |
|  |  | 37 |  | RDC53282.1 |
|  |  |  | 23 | WP 121979773.1 |

**Fig. S2.** Phylogenetic analysis of MurB using Neighbor Joining Method

92

80

57

90

63

44

99

25

79

26

48

99

94 25

25

56

80

93

99

72

12

99

63

47

76

6

91

99

99

99

24

19

26

30

70

99

99

35

23

7

8

10

99

22

12

7

78

5

90

57

0

85

86

99

15

0

99

18

4

7

20

48

95

99

99

99

94

**Fig. S3.** Phylogenetic analysis of MurB using Maximum Parsimony Method 51

17

45

97

44

79

31

82

33

99

99

56

81

99

WP 005203267.1

WP 004807589.1

WP 016163488.1

WP 005189129.1

WP 005212001.1

WP 005152500.1

WP 005293537.1

WP 065342727.1

WP 070075443.1 OJU81966.1

WP 004653193.1

WP 038344308.1

WP 005230051.1

WP 123774487.1

WP 005257213.1


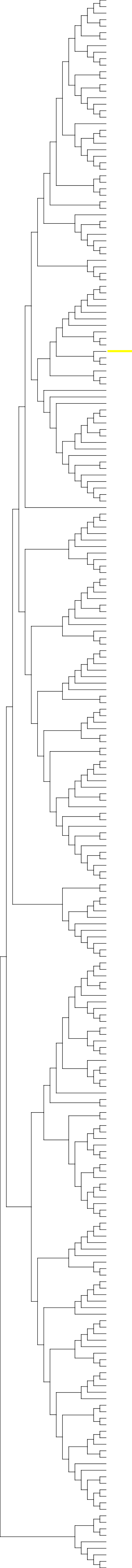
37

93

44

37

35

38

45

26 64

99

96

56

21 43

99

99

65 99

99

99

32

98

27

99

58

53

21

99

90

64

53

53

73 53

53

53

25

98 53

54

67

23

73

89

99 98

14 86

50

37 79

89

37 96

80

80

67

82

83

73

93

72

72

70

1

89

52

99

51

99

75

88

99

74

57

55

70

56

99

70

2 99

9

23

60

99

98

86

99

39

28

23

17

0 99

99

99

31

9 4

99

20

93

68

92

99

88

99 28

9

87

94

7

6

23

4

12

46

29

68

37

91

99

89

97

16 54

99

80

40

56

61

46

63

98

19

27

36

78

39

39

74

99

36

54

98

66

99

45

92

97

99

98

80

99

1

99

98

99

36

10

42

99

79

61

99

99

97 62

45

0

99

41

45

56

48

95

99

7

48

99

78

99

92

99

8 17

44

98

60

92

18

0

77

99

99

81

99

4

76

51

99

72

60

53

44

94

99

90

96

76

33

27

43

5

11

41

98

17

99

0

36

99

87

91

WP 062846995.1

WP 069577941.1

WP 004771880.1

WP 047426963.1

WP 018678197.1

WP 046739560.1

WP 004661949.1

WP 005215963.1

WP 087545799.1

WP 005160447.1

WP 087547790.1

WP 087536163.1

WP 130161378.1

WP 121929248.1

WP 004670863.1

WP 005196737.1

WP 023013599.1

WP 061525088.1

WP 005061967.1

WP 100833701.1

WP 150026059.1

WP 004761426.1

WP 004679755.1

WP 004777230.1

WP 004757822.1 TXJ03386.1

WP 129262666.1 HAK16169.1 EEY93828.1 ETY69091.1

WP 114327793.1

WP 001166916.1 ETQ95341.1 EEX04534.1 EGJ67433.1 OTL38132.1 SSQ03959.1

WP 001166920.1 EMT98785.1

MurB A.baumannii WP 001166921.1 B0VRK7.1

WP 086496969.1 HCD61322.1

WP 106465314.1 HBU88774.1

WP 005306417.1 RJE57869.1

WP 032005954.1

WP 060466592.1

WP 032038131.1

WP 033847372.1 EFF86130.1

WP 086266293.1

WP 057105832.1

WP 009390302.1

WP 133972960.1

WP 042893972.1

WP 132069363.1

WP 133746817.1

WP 056480822.1 EEY78511.1

WP 092706965.1

WP 104494655.1 EXD37990.1 ENV79435.1

WP 080776124.1 HCK31656.1

WP 120369242.1

WP 130804732.1

WP 004927209.1

WP 081408837.1 APV37518.1 KOR11649.1

WP 004752377.1

WP 131389917.1

WP 131381559.1

WP 116726100.1

WP 131378391.1

WP 131314674.1

WP 045793084.1

WP 111894322.1

WP 088822514.1

WP 092747017.1

WP 023271922.1 OUY07874.1

WP 087620029.1

WP 097078384.1

WP 070069158.1

WP 092621655.1

WP 067553711.1

WP 139852256.1

WP 016656816.1

WP 042111707.1

WP 134243271.1

WP 076876706.1 RZA05516.1

WP 114898979.1

WP 022967536.1

WP 151183966.1

WP 026951572.1

WP 135071065.1

WP 137403266.1

WP 141615335.1

WP 015266767.1

WP 112785906.1

WP 143961510.1

WP 074224298.1

WP 008200601.1

WP 111317580.1

WP 087939839.1

WP 092789489.1

WP 067547210.1

WP 133553205.1 TNF39299.1

WP 111392935.1

WP 077351401.1

WP 119478405.1

WP 092731536.1

WP 103923541.1

WP 111669820.1 AMW80367.1 WP 081406007.1

WP 086210090.1

WP 110973970.1

WP 119063450.1

WP 087527163.1 PZQ93846.1 OHC25505.1 WP 078388853.1

WP 078425051.1

WP 058869704.1

WP 062032708.1

WP 131316571.1

WP 131269383.1

WP 131382805.1

WP 131291988.1

WP 067726144.1

WP 086192676.1

WP 131271973.1

WP 131216170.1

WP 131363680.1

WP 067866885.1

WP 086175029.1

WP 086200014.1

WP 086195433.1

WP 092818312.1

WP 131274545.1

WP 086187897.1

WP 004648812.1

WP 086165846.1

WP 092688991.1

WP 086184455.1

WP 039622605.1

WP 092719028.1

WP 053578031.1

WP 092767856.1

WP 035266127.1

WP 081406150.1

WP 111859169.1

WP 107009966.1

WP 086190958.1

WP 086206045.1

WP 086203857.1

WP 130116275.1

WP 130132460.1

WP 130114153.1

WP 139422253.1

WP 046761168.1

WP 096734361.1

WP 010115534.1

WP 089604337.1

WP 120384109.1

WP 044740426.1

WP 116760892.1

WP 131321346.1

WP 131264387.1

WP 131326324.1

WP 142768629.1

WP 122897868.1

WP 131260922.1

WP 104852096.1

WP 078193980.1

WP 111884273.1

WP 078188596.1

WP 067767348.1

WP 086177911.1

WP 004866062.1

WP 005009136.1

WP 086214022.1

WP 067668051.1

WP 068912455.1

WP 068888366.1

WP 120402692.1

WP 106984903.1

WP 117007599.1

WP 076753712.1

WP 048881277.1

WP 099338924.1

WP 034589112.1

WP 067730133.1

WP 104425984.1

WP 034170542.1

WP 004783906.1

WP 125278995.1 ENW23442.1 WP 005170480.1

WP 100329600.1

WP 100535294.1

WP 100357931.1

WP 005106607.1

WP 071852098.1

WP 085064860.1

WP 005263277.1

WP 044108858.1

WP 005252175.1 RDC53282.1

WP 121979773.1

WP 054582605.1

WP 086181081.1

WP 087512378.1

WP 087550938.1

WP 121972832.1 PKH33068.1

WP 081374976.1 EEY87139.1

WP 068791239.1

**Fig. S4.** Phylogenetic analysis of MurB using Minimum Evolution Method

30

36

39

89

95

41

82

89

29 99

57

67

79

82

WP 005189129.1

WP 005212001.1

WP 016163488.1

WP 005152500.1

WP 005203267.1

WP 004807589.1

WP 005293537.1

WP 065342727.1 OJU81966.1

WP 070075443.1

WP 004653193.1

WP 018678197.1

WP 046739560.1

WP 004661949.1

WP 005215963.1

15

99

79

44

53

17

99

17

99

99

32 99

84

83

99

53

33 69

55

99

40

99

32

99 43

99

15

99

86

64

47

43

27

90 25

32

20

87

14

83

39

99

99

99

99

81

58

85

32

62

42

63

56

98 95

6

64

39

87

65

89

44

37

74

90

51

99

79

99

83

88

99

66

99

7 36

55

82

56

99

99

66

20

9

28

52

99

3

99

78

99

9

57

99

99

82

14

28

99

67

62

95

90

64

91

23

99

89

49

0 59 15

6

WP 005160447.1

WP 087545799.1

WP 087547790.1

WP 087536163.1

WP 005061967.1

WP 130161378.1

WP 121929248.1

WP 004670863.1

WP 005196737.1

WP 038344308.1

WP 005230051.1

WP 047426963.1

WP 123774487.1

WP 005257213.1

WP 062846995.1

WP 004771880.1

WP 069577941.1

WP 023013599.1

WP 061525088.1

WP 004761426.1

WP 004679755.1

WP 004777230.1

WP 004757822.1

WP 100833701.1

WP 150026059.1 TXJ03386.1

WP 129262666.1 HAK16169.1 EEY93828.1 OTL38132.1 ETQ95341.1 ETY69091.1 EGJ67433.1 EEX04534.1

WP 114327793.1

WP 001166916.1

WP 001166920.1

MurB A.baumannii WP 001166921.1 B0VRK7.1 EMT98785.1 SSQ03959.1 HCD61322.1

WP 106465314.1

WP 086496969.1

WP 033847372.1 EFF86130.1

WP 086266293.1

WP 032038131.1

WP 032005954.1

WP 060466592.1

WP 057105832.1 RJE57869.1 HBU88774.1

WP 005306417.1

WP 133972960.1

WP 042893972.1

WP 009390302.1

WP 092706965.1

WP 132069363.1

WP 056480822.1 EEY78511.1

WP 133746817.1 EXD37990.1 ENV79435.1

WP 080776124.1 HCK31656.1

WP 120369242.1

WP 130804732.1

WP 004927209.1 KOR11649.1 APV37518.1

WP 081408837.1

WP 092767856.1

WP 035266127.1

WP 004752377.1

WP 131389917.1

WP 131381559.1

WP 116726100.1

WP 045793084.1

WP 131378391.1

WP 131314674.1

WP 111894322.1

WP 088822514.1

WP 092747017.1

WP 023271922.1 OUY07874.1

WP 087620029.1

WP 097078384.1

WP 070069158.1

WP 092621655.1

WP 067553711.1

WP 134243271.1

WP 076876706.1 RZA05516.1

WP 114898979.1

WP 022967536.1

WP 151183966.1

WP 137403266.1

WP 141615335.1

WP 015266767.1

WP 112785906.1

WP 143961510.1

WP 087939839.1

WP 092789489.1

WP 135071065.1

WP 074224298.1

WP 008200601.1

WP 111317580.1

WP 026951572.1

WP 067547210.1

14

|  | | | 31 | WP 133553205.1 |
| --- | --- | --- | --- | --- |
|  |  |  |  | TNF39299.1 |
| 12 |  |  |  | WP 111392935.1 |
|  |  |  | 86 | WP 103923541.1 |
|  |  |  |  |  |
| 22 |  |  |  | WP 111669820.1 |
|  | 59 |  |  | WP 092731536.1 |
|  |  | 76 |  | WP 077351401.1 |
|  |  |  | 70 | WP 119478405.1 |
|  |  |  |  | PKH33068.1 |
|  | 99 |  |  | WP 081374976.1 |
|  |  | 73 |  | EEY87139.1 |
|  |  |  | 62 | WP 068791239.1 |
|  |  |  | 99 | WP 054582605.1 |
|  |  | 25 |  | WP 086181081.1 |
|  |  |  |  | WP 004866062.1 |
|  |  |  | 44 | WP 121972832.1 |
|  |  |  | 99 | WP 081406150.1 |
|  |  | 99 |  | WP 111859169.1 |
|  | 71 |  |  | WP 107009966.1 |
|  |  |  |  | WP 086190958.1 |
|  |  | 58 |  | WP 086206045.1 |
|  |  |  | 99 | WP 086203857.1 |
| 74 |  |  | 59 | WP 130114153.1 |
|  |  | 99 |  | WP 130132460.1 |
|  |  |  |  | WP 130116275.1 |
|  |  |  | 99 | WP 139422253.1 |
| 95 |  | 82 |  | WP 046761168.1 |
|  |  |  |  | WP 096734361.1 |
|  | 62 |  |  | WP 120384109.1 |
|  |  | 99 |  | WP 089604337.1 |
|  |  |  | 68 | WP 010115534.1 |
|  |  |  | 72 | PZQ93846.1 |
|  |  | 51 |  | OHC25505.1 |
|  | 97 |  |  | WP 078388853.1 |
| 62 |  |  |  | WP 058869704.1  WP 062032708.1 |
|  |  |  |  |  |
|  |  |  | 42 | WP 078425051.1 |
| 99 |  |  |  | WP 087527163.1 |
|  |  |  |  | WP 119063450.1 |
|  |  | 99 |  | WP 086210090.1 |
|  |  |  | 63 | WP 110973970.1 |
|  |  |  |  | AMW80367.1 |
|  |  |  | 99 | WP 081406007.1 |
|  |  |  |  | WP 139852256.1 |
|  |  | 68 |  | WP 016656816.1 |
|  |  |  | 99 | WP 042111707.1 |
|  | | |  | WP 104494655.1 |
|  | | |  | WP 039622605.1 |
|  | | | 99 | WP 092719028.1 |
|  | | |  | WP 053578031.1 |
|  | | | 63 | WP 086175029.1 |
| 51 | | |  | WP 086200014.1 |
| 99 | | |  | WP 086195433.1 |
|  | | |  | WP 092818312.1 |
|  | | | 99 | WP 131274545.1 |
|  | | | 65 | WP 004648812.1 |
| 98 | | |  | WP 086165846.1 |
| 99 | | |  | WP 092688991.1 |
|  | | | 76 | WP 086184455.1 |
|  | | |  | WP 086187897.1 |
|  | | | 99 | WP 131363680.1 |
| 58 | | |  | WP 067866885.1 |
|  | | |  | WP 131216170.1 |
|  | | | 50 | WP 131316571.1 |


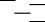


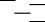


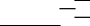


10


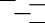


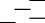


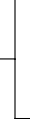

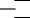
49

25


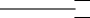
14

1

32

98

61

61

57


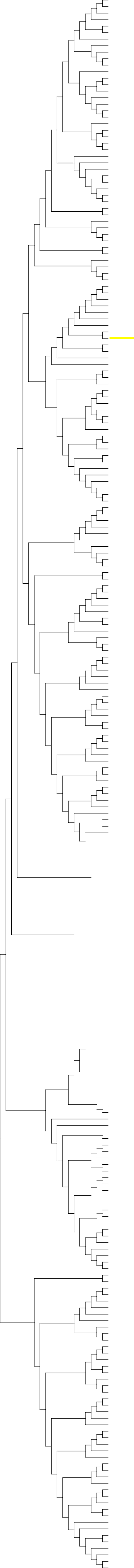


96 98

84

81

71

70

99

96

61

28

97

99

23

5

70

99

84

98

97

6 40

85

90

99

89

99

84

17

99

99

99

99

31 90

87

56

99

90

53

40

97

99

71

99

99

41

87

25

14

34

25

WP 131269383.1

WP 131382805.1

WP 131271973.1

WP 131291988.1

WP 086192676.1

WP 067726144.1

WP 087512378.1

WP 087550938.1

WP 044740426.1

WP 116760892.1

WP 131321346.1

WP 131326324.1

WP 131264387.1

WP 142768629.1

WP 122897868.1

WP 131260922.1

WP 104852096.1

WP 005009136.1

WP 067668051.1

WP 086214022.1

WP 068888366.1

WP 068912455.1

WP 120402692.1

WP 106984903.1

WP 117007599.1

WP 078193980.1

WP 111884273.1

WP 078188596.1

WP 067767348.1

WP 086177911.1

WP 076753712.1

WP 048881277.1

WP 099338924.1

WP 034589112.1

WP 067730133.1

WP 004783906.1

WP 125278995.1

WP 104425984.1

WP 034170542.1 ENW23442.1 WP 100329600.1

WP 005170480.1

WP 100535294.1

WP 100357931.1

WP 085064860.1

WP 071852098.1

WP 044108858.1

WP 005252175.1

WP 005106607.1

WP 005263277.1 RDC53282.1

WP 121979773.1

**Fig. S5.** Phylogenetic analysis of MurB using Maximum Likelihood Method

30

36

14

32

43

80

98

46

14

97

99

29

72

87

99

48

60

25

87

99

95

98

30 97

92

99

73

99

89

48

RDC53282.1

WP 121979773.1

WP 005263277.1

WP 044108858.1

WP 005252175.1

WP 005106607.1

WP 071852098.1

WP 085064860.1 ENW23442.1 WP 100329600.1

WP 005170480.1

WP 100535294.1

WP 100357931.1

WP 034170542.1

WP 104425984.1

WP 004783906.1

WP 125278995.1

WP 067730133.1

WP 034589112.1

WP 099338924.1

WP 076753712.1

WP 048881277.1

WP 086177911.1

WP 067767348.1

WP 078188596.1

WP 078193980.1

WP 111884273.1

WP 106984903.1

WP 117007599.1

WP 120402692.1

WP 068888366.1

7

2

21

99

2

93

67

99

8

97

53

2

45

45

87

13

74

3

85

45

77

49

52

82

7

32

81

80

28

21

59

22

1

93

46

57

30

23

20 72

92

47

97

43

99

45

96

32

60

50

14

99

47

60

50

44

92

43

99

45

99

99

82

99

58

98

39

99

61

57

98

51

52

35

62

95

21

48

99

99

32

49

99

51

99

99

97 74

52

99

69

95

99

56

49

96

85

99

53

49

33

26

88

59

56

92

65

58

45

58

39

83

95

57

45

32

34

20

38

46

61

75

99

50

47

99

18

99

99

99

99

99

47

46

76

99

32

WP 068912455.1

WP 067668051.1

WP 005009136.1

WP 086214022.1

WP 131260922.1

WP 104852096.1

WP 122897868.1

WP 142768629.1

WP 131326324.1

WP 131264387.1

WP 131321346.1

WP 044740426.1

WP 116760892.1

WP 104494655.1

WP 087512378.1

WP 087550938.1

WP 058869704.1

WP 062032708.1

WP 078425051.1

WP 078388853.1 OHC25505.1 PZQ93846.1

WP 087527163.1

WP 119063450.1

WP 086210090.1

WP 110973970.1

WP 092719028.1

WP 053578031.1

WP 039622605.1

WP 004648812.1

WP 086165846.1

WP 092688991.1

WP 086184455.1

WP 086187897.1

WP 092818312.1

WP 131274545.1

WP 086195433.1

WP 086175029.1

WP 086200014.1

WP 131363680.1

WP 067866885.1

WP 131216170.1

WP 131316571.1

WP 131269383.1

WP 131382805.1

WP 131271973.1

WP 131291988.1

WP 086192676.1

WP 067726144.1

WP 054582605.1

WP 086181081.1

WP 004866062.1

WP 121972832.1

WP 081406150.1

WP 111859169.1

WP 107009966.1

WP 086190958.1

WP 086206045.1

WP 086203857.1

WP 130114153.1

WP 130132460.1

WP 130116275.1

WP 139422253.1

WP 046761168.1

WP 096734361.1

WP 010115534.1

WP 089604337.1

WP 120384109.1 EXD37990.1 ENV79435.1 HCK31656.1

WP 080776124.1

WP 120369242.1

WP 130804732.1

WP 004927209.1

WP 081408837.1 APV37518.1 KOR11649.1 EEY78511.1

WP 133746817.1

WP 092706965.1

WP 056480822.1

WP 132069363.1

WP 133972960.1

WP 042893972.1

WP 009390302.1

WP 057105832.1

WP 086266293.1

WP 033847372.1 EFF86130.1

WP 032038131.1

WP 032005954.1

WP 060466592.1 RJE57869.1 HBU88774.1

WP 005306417.1

WP 086496969.1 HCD61322.1

WP 106465314.1 SSQ03959.1

WP 001166921.1 B0VRK7.1

WP 001166920.1

MurB A.baumannii EMT98785.1

WP 001166916.1 EEX04534.1

WP 114327793.1 EGJ67433.1 ETY69091.1 OTL38132.1 ETQ95341.1 TXJ03386.1 HAK16169.1 EEY93828.1

WP 129262666.1

WP 004777230.1

WP 004757822.1

WP 004679755.1

WP 004761426.1

WP 100833701.1

WP 150026059.1

WP 005061967.1

WP 023013599.1

WP 061525088.1

WP 130161378.1

WP 121929248.1

WP 004670863.1

WP 005196737.1

WP 087547790.1

WP 087536163.1

WP 087545799.1

WP 005160447.1

WP 005215963.1

WP 046739560.1


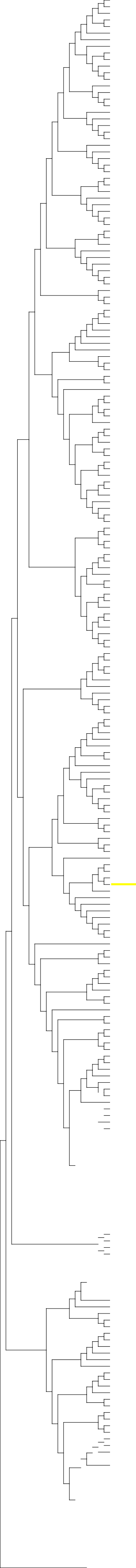

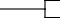


62

|  | | |  |  | 50  45 | WP 004661949.1  WP 018678197.1  WP 004771880.1 |
| --- | --- | --- | --- | --- | --- | --- |
|  |  |  |  | 61 |  | WP 069577941.1 |
|  |  |  | 49 |  |  | WP 062846995.1 |
|  |  | 69 |  |  |  | WP 123774487.1 |
|  | 99 |  |  |  | 61 | WP 005257213.1  WP 047426963.1  WP 038344308.1 |
|  |  |  |  |  | 54  73 | WP 005230051.1  OJU81966.1 |
| 38 |  |  |  | 99 |  | WP 004653193.1 |
|  |  |  | 81 |  | 18 | WP 070075443.1  WP 065342727.1  WP 005152500.1 |
|  | 84 |  |  |  | 36 | WP 005293537.1  WP 005203267.1 |
|  |  | 91 |  |  |  | WP 004807589.1 |
|  |  |  | 39 |  |  | WP 016163488.1 |
|  |  |  |  | 18 |  | WP 005189129.1 |
|  |  |  |  |  | 28  99 | WP 005212001.1  AMW80367.1 |
|  |  |  |  |  |  | WP 081406007.1 |
|  |  |  |  | 14 |  | WP 092767856.1 |
|  |  |  |  |  | 99  33 | WP 035266127.1  WP 004752377.1 |
|  |  |  |  | 49 |  | WP 131389917.1 |
|  |  | 42 | 68 |  |  | WP 131381559.1  WP 116726100.1 |
|  | 99 |  |  |  |  | WP 131378391.1 |


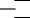


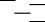


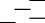


63 WP 131314674.1

99

WP 045793084.1

26 WP 111894322.1

WP 088822514.1

WP 092747017.1

40

61 WP 023271922.1

99 OUY07874.1

99 WP 087620029.1

87

WP 097078384.1

99

8 WP 070069158.1

25

WP 092621655.1

WP 067553711.1

99 WP 134243271.1

98 WP 076876706.1

87

RZA05516.1

3 WP 114898979.1

49

WP 022967536.1

99 WP 151183966.1

93 WP 087939839.1

4 WP 092789489.1

99 WP 026951572.1

99

|  | |  |  | 32  72 | WP 135071065.1  WP 137403266.1 |
| --- | --- | --- | --- | --- | --- |
|  |  |  | 95 |  | WP 141615335.1 |
|  |  | 99 |  |  | WP 015266767.1 |
|  | 74 |  |  |  | WP 112785906.1 |
| 12 |  |  |  |  | WP 143961510.1 |
|  |  |  |  |  | WP 111317580.1 |
|  |  |  | 28 |  | WP 074224298.1 |
|  |  |  |  | 97 | WP 008200601.1  WP 067547210.1 |
|  |  |  |  |  | WP 133553205.1 |
|  |  |  |  | 31 | WP 111392935.1 |
| 21 |  |  |  |  | TNF39299.1 |
|  |  |  |  | 33 | WP 077351401.1 |
|  | 16 |  |  |  | WP 119478405.1 |
|  |  | 88 |  |  | WP 092731536.1 |
|  |  |  | 60 |  | WP 103923541.1 |
|  |  |  |  | 94 | WP 111669820.1 |
|  |  |  |  | 99 | WP 016656816.1 |
|  |  |  | 61 |  | WP 042111707.1 |
|  |  |  |  |  | WP 139852256.1 |
|  | 2 |  |  |  | PKH33068.1 |
|  |  | 99 |  |  | WP 081374976.1 |
|  |  |  | 76 |  | EEY87139.1 |
|  |  |  |  | 67 | WP 068791239.1 |


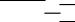


1

8


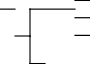


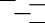


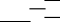


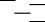


## **Fig. S6.** Phylogenetic analysis of MurE using UPGMA Method

98 WP 086194200.1

74 WP 067726842.1

72

WP 131270817.1

90 WP 131318161.1

36 WP 131383108.1

41

100

63 WP 131268425.1

WP 131289315.1

WP 131217394.1

WP 131366654.1

85

99 WP 067868911.1

WP 086187252.1

100

90 99

69

99

WP 004650609.1

WP 086164725.1

WP 092689528.1

WP 086184323.1

100 WP 086174564.1

29 WP 086198627.1

100 WP 086197214.1

90

100

WP 092820212.1

WP 131273125.1

12

100

66

WP 122900110.1

WP 120365257.1

WP 117006905.1

WP 054580882.1

53

70

19 72

56

11

WP 005013219.1

WP 067670237.1

WP 086213162.1

WP 068889205.1

WP 068912911.1

21 WP 067763012.1

100

6

67

100

43

WP 086178249.1

WP 111884134.1

WP 078189388.1

WP 078192537.1

WP 053579412.1

91

100

WP 116759022.1

WP 131264644.1

100 WP 131325498.1

WP 044739701.1

100 WP 151504401.1

94

74

100

WP 086209051.1

WP 119063246.1

WP 110974181.1

100 WP 087527288.1

22

100

66

75

33

71

37

WP 062032174.1

WP 078389189.1 OHC24591.1 WP 130235299.1

WP 078425520.1 OYW72299.1

100 WP 104505688.1

WP 005180534.1

99 WP 076754508.1

100 WP 048881755.1

93

66

24

93

76

100

92

WP 034588444.1

WP 099338116.1

WP 125278889.1

WP 004787142.1

WP 034169735.1

WP 067731297.1

62

99 100

WP 100357759.1

WP 005168898.1

WP 100355178.1

100

99

100

100

73

WP 064102372.1

WP 064094608.1

WP 004281132.1 RDC51980.1

87 WP 005249723.1

69 WP 005248376.1

WP 039620015.1

100

100

WP 121972437.1 SPL68826.1

WP 004869857.1

100 WP 086205109.1

97 WP 086202419.1

WP 086189721.1

97 94

WP 065993809.1

WP 111858924.1

53 71

100

WP 130112079.1

WP 130168347.1

24 WP 130116047.1

95

WP 087512119.1

100 WP 087549832.1

52

100

50

45

WP 089605292.1

WP 120382691.1

WP 010113046.1

WP 107007166.1

78

18 100

WP 046760384.1 CEI53614.1

WP 139419352.1

99 WP 035370565.1

100

27

54

WP 034615061.1

WP 105713827.1

WP 056517500.1

100

100

98

WP 125269342.1

WP 049173979.1

WP 120370150.1

16 WP 130803677.1

WP 076033130.1

98

100

WP 004923700.1 CAG70031.1

39 WP 004754564.1

68 WP 131388641.1

92

45

100

WP 131379642.1

WP 116726658.1

WP 004898992.1

WP 131377335.1

94 WP 131312914.1

82 WP 005300947.1

76 HBU88269.1

97

WP 107880550.1

75

WP 049837451.1

62 WP 009391973.1

WP 092709869.1

100

WP 133747408.1

99

WP 132070469.1

37

29 WP 056482427.1

WP 016143668.1

41

100

98 53

17

61

14

17

46

WP 033848919.1

WP 133481337.1

WP 046813100.1

WP 032036604.1

WP 064784614.1

WP 032004897.1

WP 032057240.1

59 WP 032027505.1

99 WP 031956431.1

WP 135924482.1

72 WP 086496378.1

50

100 WP 019457881.1

WP 077163642.1

86 MurE from Acinetobacter baumannii

37

98 WP 000218541.1

99 WP 000218542.1

WP 002060058.1

57

60 WP 125564060.1

100

100

WP 004964616.1 OFW43372.1

PRD37030.1

100

88

56

100

WP 130160844.1

WP 121928940.1

WP 005193875.1

WP 004673224.1

WP 150024845.1

90 100

WP 023013852.1

WP 061393187.1

42

68 44

90

28

WP 005156610.1

WP 087535961.1

WP 087547386.1

WP 087543705.1

35 42

100

95

WP 005219446.1

36 WP 004660124.1 OJU98723.1

WP 050041017.1

WP 018678830.1

100

27

WP 004760517.1

WP 004797689.1

WP 005052228.1

WP 109441014.1

26

40 WP 005260000.1

46

35 90

39

53

31

100

WP 047430304.1

WP 062846560.1

WP 069576624.1

WP 123775346.1

WP 004776069.1

38 WP 005227678.1

99 WP 009506724.1

WP 038346217.1

22 66

100

WP 070076099.1

WP 004657406.1


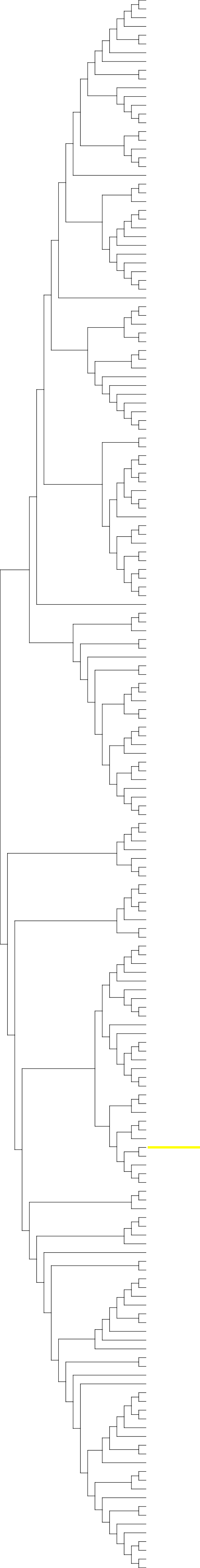


WP 016542716.1

WP 005275806.1

22

42 WP 005147361.1

29 WP 005297385.1

WP 016162313.1

74

WP 005209251.1

82

98 WP 005317638.1

24 WP 033132641.1

37 WP 005185552.1

34 WP 004803894.1

## **Fig. S7.** Phylogenetic analysis of MurE using Neighbor Joining Method

45 WP 131383108.1

28 WP 131268425.1

WP 131366654.1

43 100

WP 067868911.1

70 WP 131270817.1

WP 131318161.1

28

84 WP 086194200.1

100 84 WP 067726842.1

WP 131217394.1

88

WP 131289315.1

WP 086187252.1

47 100

99

81

97

49

WP 004650609.1

WP 086164725.1

WP 092689528.1

WP 086184323.1

WP 053579412.1

99 WP 086174564.1

WP 086198627.1

100

29

87

100

WP 086197214.1

WP 092820212.1

WP 131273125.1

WP 122900110.1

100

68

WP 120365257.1

WP 117006905.1

WP 054580882.1

19

63 WP 005013219.1

67 WP 067670237.1

55

36 WP 086213162.1

61

WP 068889205.1

WP 068912911.1

17

50 WP 067763012.1

40

WP 086178249.1

100 WP 078189388.1

100

55

WP 078192537.1

WP 111884134.1

100 WP 104505688.1

WP 005180534.1

99 WP 076754508.1

99 WP 048881755.1

99

72

38

50

100

93

WP 034588444.1

WP 099338116.1

WP 125278889.1

WP 004787142.1

WP 034169735.1

71 WP 067731297.1

100

81

100

WP 100357759.1

WP 005168898.1

WP 100355178.1

100

100

WP 064102372.1

WP 064094608.1

49 WP 004281132.1

99

RDC51980.1

91 WP 005249723.1

92 WP 005248376.1

89 WP 116759022.1

99 WP 131264644.1

100

82

WP 131325498.1

WP 044739701.1

100 WP 151504401.1

92

72

100

WP 086209051.1

WP 119063246.1

WP 110974181.1

100

WP 087527288.1

100

99

61

WP 062032174.1

39 WP 078389189.1 OHC24591.1

86 WP 130235299.1

WP 078425520.1

57

45 OYW72299.1

WP 039620015.1

100

100

WP 121972437.1 SPL68826.1

WP 004869857.1

100

100

WP 086189721.1

WP 086205109.1

97

100

WP 086202419.1

WP 087512119.1

WP 087549832.1

79

99 WP 065993809.1

69

48

100

WP 111858924.1

WP 130112079.1

WP 130168347.1

88 66 WP 130116047.1

51

99

51 63

WP 089605292.1

WP 010113046.1

WP 120382691.1

WP 107007166.1

85

47 100

WP 046760384.1 CEI53614.1

WP 139419352.1

96 WP 105713827.1

100

41

71

WP 035370565.1

WP 034615061.1

WP 056517500.1

100

100

93

WP 125269342.1

WP 049173979.1

WP 120370150.1

75 WP 130803677.1

100 WP 076033130.1

100

24

100

86

WP 004923700.1 CAG70031.1

WP 131377335.1

WP 131312914.1

100

84

69

27

51

WP 004898992.1

WP 131388641.1

WP 004754564.1

WP 131379642.1

WP 116726658.1

91

100

WP 004964616.1 OFW43372.1

PRD37030.1

85

74

100

69

WP 130160844.1

WP 121928940.1

WP 005193875.1

WP 004673224.1

100

100

WP 004760517.1

WP 004797689.1

50 OJU98723.1

27 WP 005219446.1

38

WP 050041017.1

100

94

WP 004660124.1

WP 087547386.1

99 90

39

49

WP 087535961.1

WP 005156610.1

WP 087543705.1

WP 018678830.1

100 WP 023013852.1

27 WP 061393187.1

99

77 WP 109441014.1

WP 150024845.1

58

17 39

WP 123775346.1

WP 047430304.1

45 WP 005260000.1

WP 062846560.1

99

33 WP 069576624.1

24

66

WP 004776069.1

86 WP 005227678.1

100 WP 009506724.1

WP 038346217.1

18 83

100

WP 070076099.1

WP 004657406.1

WP 016542716.1

WP 005052228.1

39

WP 005275806.1

54 61 WP 005147361.1

40 WP 005297385.1

91 90

99 WP 005317638.1

WP 033132641.1

33 WP 005185552.1

87

WP 004803894.1

37 WP 016162313.1

47 WP 005209251.1

86 MurE from Acinetobacter baumannii

65 WP 000218541.1

87

WP 000218542.1

88

WP 002060058.1

WP 125564060.1

100

86 WP 086496378.1

81 WP 019457881.1

68 WP 077163642.1

74 WP 135924482.1

100

69

WP 032027505.1

WP 031956431.1


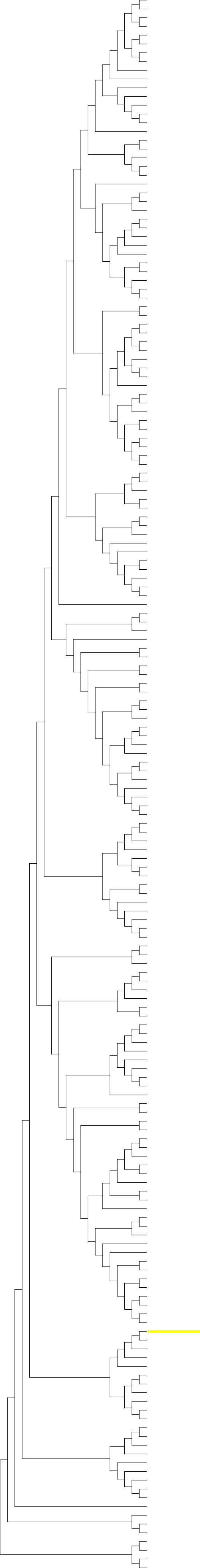


96 WP 132070469.1

70 WP 056482427.1

99

WP 133747408.1

WP 092709869.1

54 WP 009391973.1

WP 049837451.1

90

WP 107880550.1

98

WP 005300947.1

59

69 HBU88269.1

WP 016143668.1

WP 032036604.1

WP 133481337.1

25

57 WP 046813100.1

22 WP 064784614.1

WP 032057240.1

9 WP 032004897.1

53 WP 033848919.1

## **Fig. S8.** Phylogenetic analysis of MurE using Minimum Evolution

68 WP 131270817.1

69 WP 131318161.1

WP 086194200.1

28

89 WP 067726842.1

37

WP 131217394.1

100

WP 131289315.1

56 WP 131383108.1

43 34

WP 131268425.1

WP 131366654.1

100 WP 067868911.1

WP 053579412.1

33 35

36

100

98

67

98

WP 086187252.1

WP 004650609.1

WP 086164725.1

WP 092689528.1

WP 086184323.1

WP 039620015.1

100 WP 086174564.1

WP 086198627.1

100

31

97

100

WP 086197214.1

WP 092820212.1

WP 131273125.1

WP 122900110.1

100

77

WP 120365257.1

WP 117006905.1

WP 054580882.1

38

65 WP 005013219.1

54 WP 067670237.1

50

44 WP 086213162.1

93

WP 068889205.1

WP 068912911.1

19

70 WP 067763012.1

52

WP 086178249.1

100 WP 078189388.1

100

52

WP 078192537.1

WP 111884134.1

100 WP 104505688.1

WP 005180534.1

99 WP 076754508.1

100 WP 048881755.1

93

88

56

83

100

93

WP 034588444.1

WP 099338116.1

WP 125278889.1

WP 004787142.1

WP 034169735.1

84 WP 067731297.1

82

98 100

WP 100357759.1

WP 005168898.1

WP 100355178.1

100 WP 064102372.1

99

WP 064094608.1

56 WP 004281132.1

100

RDC51980.1

94 WP 005249723.1

93 WP 005248376.1

89

100

WP 116759022.1

WP 131264644.1

100 WP 131325498.1

WP 044739701.1

100

82

100

100

94

WP 151504401.1

WP 086209051.1

WP 119063246.1

WP 110974181.1

100

WP 087527288.1

100

57

WP 062032174.1

45 WP 078389189.1 OHC24591.1

72 WP 130235299.1

WP 078425520.1

59

41 OYW72299.1

100

100

WP 121972437.1 SPL68826.1

WP 004869857.1

100

48

WP 086205109.1

WP 086202419.1

99

WP 086189721.1

100 WP 087512119.1

WP 087549832.1

98

49

100

67

26

57

WP 130112079.1

WP 130168347.1

WP 130116047.1

WP 065993809.1

99 WP 111858924.1

55

100

32

67

WP 089605292.1

WP 010113046.1

WP 120382691.1

WP 107007166.1

84

31 100

WP 046760384.1 CEI53614.1

WP 139419352.1

93 WP 105713827.1

100

33

66

WP 035370565.1

WP 034615061.1

WP 056517500.1

100

100

93

WP 125269342.1

WP 049173979.1

WP 120370150.1

100

64 WP 130803677.1

WP 076033130.1

100

22

100

91

WP 004923700.1 CAG70031.1

WP 131377335.1

WP 131312914.1

100

28

84

WP 004898992.1

WP 004754564.1

WP 131388641.1

60 WP 131379642.1

52 WP 116726658.1

98

100

WP 004964616.1 OFW43372.1

PRD37030.1

66 OJU98723.1

27 WP 005219446.1

56

WP 050041017.1

WP 004660124.1

100

41 WP 005156610.1

100 WP 087543705.1

98

91 WP 087547386.1

33 WP 087535961.1

WP 018678830.1

86 WP 130160844.1

100 WP 121928940.1

WP 004673224.1

91

64

53 WP 005193875.1

WP 004760517.1

100 WP 004797689.1

100 WP 023013852.1

99 47

WP 061393187.1

78 WP 109441014.1

WP 150024845.1

51

50 35

48

WP 123775346.1

WP 047430304.1

WP 005260000.1

WP 062846560.1

99

41 WP 069576624.1

43

74

WP 004776069.1

88 WP 005227678.1

100 WP 009506724.1

WP 038346217.1

24 79

100

WP 070076099.1

WP 004657406.1

WP 016542716.1

WP 005052228.1

49

WP 005275806.1

56 71 WP 005147361.1

53 WP 005297385.1

99 WP 005317638.1

96

46 WP 033132641.1

53 WP 005185552.1

78

WP 004803894.1

43 WP 016162313.1

41 WP 005209251.1

88 MurE from Acinetobacter baumannii

66 WP 000218541.1

92

WP 000218542.1

96

WP 002060058.1

WP 125564060.1

100

86 WP 086496378.1

84 WP 019457881.1

52 WP 077163642.1

69 WP 135924482.1

100

77

WP 032027505.1

WP 031956431.1

90

78

100

WP 132070469.1

WP 056482427.1

WP 133747408.1


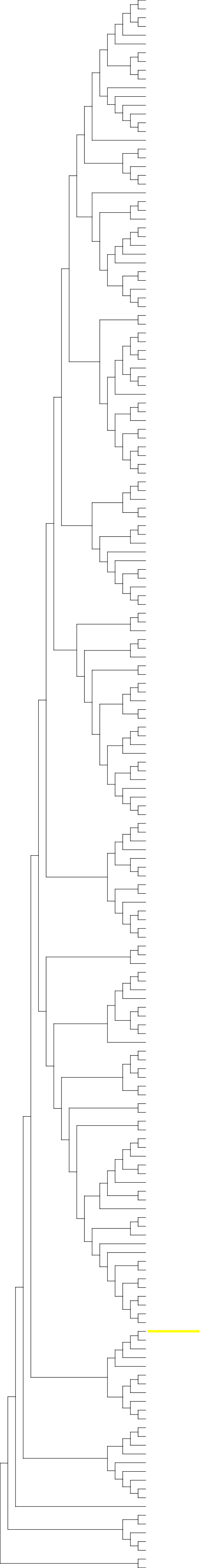


WP 092709869.1

77 WP 009391973.1

WP 049837451.1

96

WP 107880550.1

97

WP 005300947.1

53

70 HBU88269.1

WP 016143668.1

24 WP 064784614.1

WP 032057240.1

9 WP 032036604.1

WP 133481337.1

45

64 WP 046813100.1

WP 032004897.1

54 WP 033848919.1

## **Fig. S9.** Phylogentic analysis of MurE using Maximum Parsimony method

41

28

65

98

WP 005013219.1

WP 067670237.1

WP 086213162.1

WP 068912911.1

34 WP 068889205.1

WP 067763012.1

100

62

41

100

76

WP 086178249.1

WP 078189388.1

WP 078192537.1

WP 111884134.1

23

66

100

WP 054580882.1

WP 120365257.1

WP 117006905.1

100 WP 104505688.1

WP 005180534.1

98 WP 086174564.1

27

26 WP 086198627.1

99 WP 086197214.1

99

100

WP 092820212.1

WP 131273125.1

WP 122900110.1

WP 053579412.1

11 WP 039620015.1

26

24 99

100

84

85

WP 092689528.1

WP 086184323.1

WP 086164725.1

WP 004650609.1

WP 086187252.1

WP 131289315.1

66

29 32

99

100

40

WP 131366654.1

WP 067868911.1

WP 131268425.1

WP 131383108.1

70 WP 086194200.1

32

WP 067726842.1

25 WP 131270817.1

WP 131217394.1

39

52 WP 131318161.1

100 WP 076754508.1

93 WP 048881755.1

WP 034588444.1

96

51 WP 099338116.1

54

100

98

WP 125278889.1

WP 004787142.1

WP 034169735.1

77 WP 067731297.1

77

99

100

WP 100357759.1

WP 005168898.1

WP 100355178.1

99

100

99

RDC51980.1

WP 064102372.1

WP 064094608.1

48 WP 004281132.1

WP 005249723.1

65

97 WP 005248376.1

84 WP 116759022.1

94 WP 131264644.1

100 WP 131325498.1

WP 044739701.1

99

79 100

100

99

WP 151504401.1

WP 086209051.1

WP 119063246.1

WP 110974181.1

100

94

WP 062032174.1

31 WP 078389189.1 OHC24591.1

23 WP 087527288.1

WP 078425520.1

29

WP 130235299.1

67

65 OYW72299.1

100

99

WP 121972437.1 SPL68826.1

WP 004869857.1

100

29

97

WP 086205109.1

WP 086202419.1

WP 086189721.1

100 WP 087512119.1

86

96

100

WP 087549832.1

WP 130116047.1

WP 130168347.1

18 WP 130112079.1

51

WP 065993809.1

99 WP 111858924.1

100

58

16 99

WP 089605292.1

WP 010113046.1

WP 120382691.1

42

97

100

56

WP 107007166.1

WP 046760384.1 CEI53614.1

WP 139419352.1

98 WP 105713827.1

100

69

48

WP 035370565.1

WP 034615061.1

WP 056517500.1

99

76

100

WP 125269342.1

WP 049173979.1

WP 120370150.1

53 WP 130803677.1

WP 076033130.1

99

63

100

WP 004923700.1 CAG70031.1

38 WP 004754564.1

WP 131388641.1

100

42

48

77

93

WP 131379642.1

WP 116726658.1

WP 004898992.1

WP 131377335.1

WP 131312914.1

78 WP 086496378.1

44 WP 019457881.1

57 WP 077163642.1

20

WP 135924482.1

14

WP 032027505.1

99

97

96 WP 031956431.1

88 WP 002060058.1

WP 125564060.1

91 WP 000218542.1

MurE from Acinetobacter baumannii

98

97 WP 000218541.1

67 WP 133481337.1

40 WP 046813100.1

100

32 WP 032036604.1

WP 032004897.1

63

77 WP 033848919.1

71 WP 064784614.1

33 WP 032057240.1

WP 016143668.1

95

11 59

63

65 86

WP 009391973.1

WP 005300947.1 HBU88269.1

WP 107880550.1

WP 049837451.1

39 WP 133747408.1

WP 056482427.1

99

WP 132070469.1

96

95 WP 092709869.1

90

100

WP 004964616.1 OFW43372.1

8

100

13

PRD37030.1

WP 004760517.1

WP 004797689.1

35 WP 004673224.1

100

69

99

WP 005193875.1

WP 130160844.1

WP 121928940.1

WP 023013852.1

100 WP 061393187.1

WP 018678830.1

49 OJU98723.1

99 WP 005219446.1

23 WP 050041017.1

99

WP 004660124.1

26 WP 087543705.1

WP 087547386.1

95

WP 005156610.1

70

64 WP 087535961.1

62 WP 123775346.1

48 WP 047430304.1

21

WP 005260000.1

31

WP 069576624.1

86

WP 062846560.1

80 WP 004776069.1

WP 038346217.1

49

100

6

WP 005227678.1

WP 009506724.1

WP 005052228.1

WP 005275806.1

26 56 WP 005147361.1

29 WP 005297385.1

100

79

WP 005317638.1

WP 033132641.1

41 WP 005185552.1

59

WP 004803894.1

49 WP 016162313.1

38 WP 005209251.1

57 WP 109441014.1

WP 150024845.1

13 WP 016542716.1


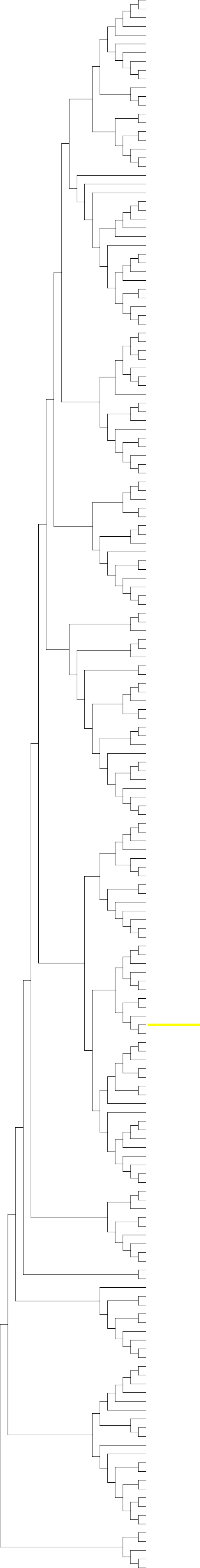


73

100

WP 070076099.1

WP 004657406.1

## **Fig. S10.** Phylogenetic analysis of MurE using Maximum Likelihood Method

25 WP 005185552.1

55 WP 004803894.1

WP 016162313.1

66

30 WP 005209251.1

WP 005317638.1

81

98 WP 033132641.1

WP 005147361.1

26

62 WP 005297385.1

WP 016542716.1

25

|  | | 72 |  | WP 070076099.1 |
| --- | --- | --- | --- | --- |
| 17 |  |  | 100 | WP 004657406.1  WP 005052228.1 |
|  |  |  |  |  |
|  |  |  | 24 | WP 005275806.1 |
|  |  |  |  | WP 109441014.1 |

22 77

WP 150024845.1

100

49

WP 005227678.1

WP 009506724.1

WP 038346217.1

WP 004776069.1

87

27 WP 062846560.1

31

82 WP 069576624.1

31 WP 005260000.1

WP 123775346.1

46

57 WP 047430304.1

WP 018678830.1

99

30

100

WP 004660124.1

WP 050041017.1

50 OJU98723.1

26 WP 005219446.1

48 WP 005156610.1

26

WP 087543705.1

65 91

WP 087547386.1

27 WP 087535961.1

WP 023013852.1

100 WP 061393187.1

100 WP 004760517.1

100

WP 004797689.1

60 WP 004673224.1

100

64

98

WP 005193875.1

WP 130160844.1

WP 121928940.1

PRD37030.1

100

71

WP 004964616.1 OFW43372.1

76 WP 086496378.1

46 WP 019457881.1

69

63 WP 077163642.1

WP 135924482.1

97 99

88

WP 032027505.1

WP 031956431.1

WP 125564060.1

WP 002060058.1

92

WP 000218542.1

58

MurE from Acinetobacter baumannii

69

90 WP 000218541.1

100

60 WP 133481337.1

32 WP 046813100.1

13 WP 032036604.1

WP 032004897.1

78

57 WP 033848919.1

WP 064784614.1

23 WP 032057240.1

99

92 WP 016143668.1

79

44

17 98

WP 132070469.1

WP 056482427.1

WP 133747408.1

WP 092709869.1

56 WP 009391973.1

WP 049837451.1

63

WP 107880550.1

93

WP 005300947.1

59

71 HBU88269.1

99

58

100

WP 125269342.1

WP 049173979.1

WP 130803677.1

57 WP 120370150.1

89 WP 076033130.1

100

100

WP 004923700.1 CAG70031.1

59 90

37

WP 131377335.1

WP 131312914.1

WP 004898992.1

100 WP 116726658.1

WP 131379642.1

83

WP 004754564.1

52

59 WP 131388641.1

100

100

WP 121972437.1 SPL68826.1

WP 004869857.1

100

32

WP 086205109.1

WP 086202419.1

99 WP 086189721.1

43

41 100

WP 130116047.1

WP 130112079.1

93 32

WP 130168347.1

WP 065993809.1

99 WP 111858924.1

100 WP 087512119.1

79

WP 087549832.1

77

100

32

WP 089605292.1

WP 010113046.1

WP 120382691.1

53

90

100

44

WP 107007166.1

WP 046760384.1 CEI53614.1

WP 139419352.1

99 WP 105713827.1

100

66

46

WP 035370565.1

WP 034615061.1

WP 056517500.1

WP 039620015.1

WP 053579412.1

100

99

WP 092820212.1

WP 131273125.1

100 WP 086197214.1

WP 086174564.1

30

94 WP 086198627.1

99 WP 092689528.1

73 WP 086184323.1

82

27 WP 086164725.1

99

WP 004650609.1

WP 086187252.1

35 WP 131217394.1

74 WP 131289315.1

34 WP 131383108.1

25 WP 131268425.1

99

WP 131366654.1

100 WP 067868911.1

19 70

WP 131270817.1

WP 131318161.1

69 WP 086194200.1

59 WP 067726842.1

38 WP 005013219.1

49 WP 086213162.1

67

WP 067670237.1

90

WP 068912911.1

44 WP 068889205.1

WP 086178249.1

100

56

10

37

100

74

75

100

WP 067763012.1

WP 078189388.1

WP 078192537.1

WP 111884134.1

WP 054580882.1

WP 120365257.1

WP 117006905.1

WP 122900110.1

53 WP 130235299.1

64 OYW72299.1

23 WP 078425520.1

56

95

9 100

WP 078389189.1

26 OHC24591.1

WP 062032174.1

WP 087527288.1

WP 110974181.1

100

78

99

WP 086209051.1

WP 119063246.1

100 WP 044739701.1

WP 151504401.1

100 WP 131325498.1

WP 116759022.1

93

80 WP 131264644.1

13 100

WP 104505688.1

WP 005180534.1

100 WP 076754508.1

96 WP 048881755.1

99

73

45

63

100

99

WP 034588444.1

WP 099338116.1

WP 125278889.1

WP 004787142.1

WP 034169735.1

WP 067731297.1

100

75

100

WP 100357759.1

WP 005168898.1

WP 100355178.1

100

99

100

RDC51980.1

WP 064102372.1

WP 064094608.1


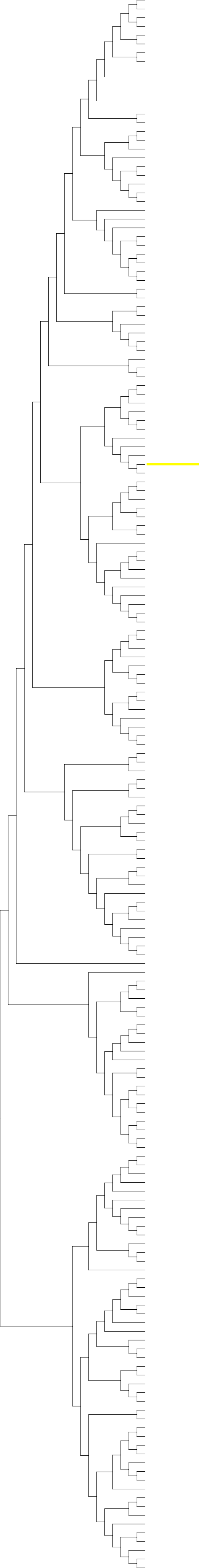

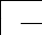


54 WP 004281132.1

WP 005249723.1

60

95 WP 005248376.1

**Fig. S11.** Phylogenetic analysis of MurG using UPGMA Method

26

36

80

64

99 82

47

59

32

99

99 95

81

23 95

55

88

17 88

99

95

56

71

9

90

99

93

74

99

86

12

99

4 99

72

99

61

73

35

31

79

61

99

27

99

59

8

91

73

31

35

76

31

64

96

90

99

87

54

84

9 96

95

WP 086193803.1

WP 067725439.1

WP 131288345.1

WP 131271374.1

WP 131317157.1

WP 131384047.1

WP 131267760.1

WP 131216843.1

WP 131366157.1

WP 067869251.1

WP 086186928.1

WP 086165337.1 SFT09247.1

WP 074947103.1 ENU21062.1

WP 086184053.1 SDY40554.1

WP 092689979.1

WP 053579255.1 SCC72224.1

WP 092720068.1

WP 039620241.1

WP 044738880.1 EOR02599.1

WP 131325623.1

WP 131324192.1

WP 116759136.1

WP 131264210.1

WP 142769955.1

WP 092820539.1

WP 131274197.1

WP 086196542.1

WP 086174703.1

WP 086199014.1

WP 110974620.1 ENV74306.1

WP 087526678.1 ENU37982.1

WP 114392648.1

WP 062031947.1 SNU14554.1 OHC23638.1 WP 104500254.1

WP 005180817.1

WP 104852726.1

WP 019837075.1

WP 070155856.1

WP 067668390.1

WP 086211587.1

WP 005006719.1

WP 068885870.1

WP 068910292.1

WP 120365498.1

WP 120402735.1

WP 086177223.1

WP 067761632.1

WP 078193220.1

WP 078190577.1

WP 111884460.1

WP 004813763.1

WP 008305759.1

WP 005220556.1

WP 099337732.1

11

69

48

57

99

84

77

96

79

18 90

48

49

51

22

23

43

18

85

99

81

99

99

99

99

80

99

96

82 90

66

42

99

82

83

35

42

69

82 99

88

39 42 99

39

99

84

99

18

20

21

21

19

23

95 16

21

66

23

86

99 58

80

54

65

99 25

99

32

21

84 23

99

97

85

99

82

99

60

40

75 48

WP 034588633.1

WP 125279159.1

WP 067727838.1

WP 100534533.1

WP 100356984.1

WP 100354939.1 ENW25488.1 EAM8864034.1 WP 148335071.1

WP 005245707.1 RDC53559.1

WP 121979730.1

WP 005250316.1

WP 044111339.1

WP 071850281.1

WP 122900661.1

WP 054580793.1

WP 086180164.1

WP 092766516.1

WP 035265837.1

WP 087512016.1

WP 087549744.1 SPL68923.1

WP 121972497.1 ENV31818.1

WP 020846716.1

WP 067663080.1

WP 086205195.1

WP 086204224.1

WP 086189828.1

WP 065993089.1

WP 111858988.1

WP 130072389.1

WP 107009456.1

WP 010112319.1

WP 120383482.1 ENU60003.1

WP 016539102.1

WP 056517158.1

WP 105713898.1 ENV17748.1 ENV89452.1

WP 010590909.1 ENV19691.1

WP 130116870.1

WP 130112832.1

WP 130131070.1 HCM30849.1 WP 034683297.1

MurG from Acinetobacter baumannii WP 000132435.1

WP 140953803.1

WP 000132433.1

WP 069036603.1 ETR82976.1

WP 071210169.1 SSM90614.1

WP 100290049.1

WP 019458531.1

WP 077168837.1

WP 057106088.1

WP 033849065.1 EOQ60907.1

WP 111855215.1

WP 009391329.1

WP 133971229.1 ENW01981.1 WP 132070613.1

WP 133747326.1

WP 056482157.1

WP 092708471.1 EXA67995.1

WP 048765277.1

WP 088458630.1

WP 004923335.1

WP 130803940.1

WP 120369847.1 BBF76985.1 ENV78217.1

WP 049174423.1 ENV64477.1 OFW44053.1

28

| 99 |  |  | HAK15860.1 |
| --- | --- | --- | --- |
|  |  |  | EPR86420.1 |
|  |  | 63  63 | ENV51313.1  ENW22118.1 |
|  | 88 |  | WP 017395615.1 |
| 99 |  |  | EEH68243.1 |
|  |  |  | ENW20351.1 |
|  |  | 82  90 | WP 150024750.1  WP 130160754.1 |


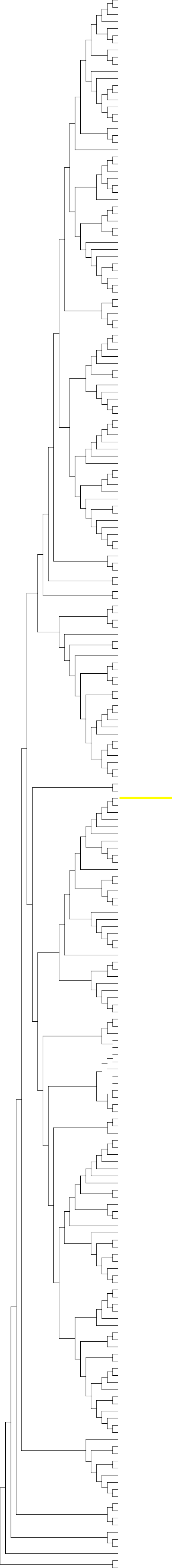


99

49

99

96

83

99

40

23

83

26

36

62

34

12

58

46

29 88

98

99

40

99

54

63

80

99

52

64

51

51

64

56

99

41

27

99

97

68

99

29

78

62

25 69

61

50

77

54

46

38

94

85

99

49

99

90

32

20

21

84

91

99

99

99

99

WP 121929032.1

WP 081398975.1

WP 081400731.1 ENU35076.1

WP 081399900.1

WP 081399063.1 ENV10142.1 ENU21674.1

WP 032864351.1

WP 101235492.1

WP 081406930.1

WP 081401814.1

WP 081401904.1

WP 081401950.1

WP 081402010.1 EPF93277.1 ESK38007.1

WP 081403844.1

WP 081401650.1

WP 081407343.1 ENX09243.1

WP 009510197.1 KYQ85260.1

WP 081410353.1 ENU93267.1 RPE30045.1

WP 123775416.1 KXO80407.1 HBO71187.1 PRD19162.1

WP 019385625.1 ENV38228.1 ENU33449.1 ENW07184.1 WP 034710633.1 ENW06013.1 ESK55389.1

WP 018679367.1

WP 081399858.1

WP 087547617.1

WP 087537056.1

WP 087543583.1

WP 081408195.1

WP 081399778.1 OJU89657.1

WP 081401574.1 KKW76424.1 WP 081407495.1

WP 111895072.1

WP 131375671.1

WP 131312428.1

WP 045793218.1

WP 131388464.1

WP 116727941.1 ENV47184.1

WP 034425615.1

WP 131382055.1 TCM68745.1

WP 139853478.1 EPF81552.1

WP 040330960.1

WP 087620370.1 SNX45987.1

WP 097079742.1

WP 067558430.1 ESK38773.1

WP 040331973.1

**Fig. S12.** Phylogenetic Analysis of MurG using Neighbor Joining Method

87

44

44

53

6 86

99

24

97

15

99

83

86

64

24 35

42

99

58

60

31

99

45 70

64

39

99

86

64

56

99

63

52

98

88 52

KKW76424.1 WP 081407495.1

WP 081401574.1 OJU89657.1

WP 081408195.1

WP 081399778.1 ESK55389.1

WP 018679367.1 ENW06013.1 ENW07184.1 WP 034710633.1

WP 081399858.1

WP 087547617.1

WP 087537056.1

WP 087543583.1 ENV38228.1 KXO80407.1 HBO71187.1 PRD19162.1

WP 019385625.1 ENU33449.1

WP 081399063.1 ENU35076.1

WP 081399900.1 KYQ85260.1

WP 081410353.1 ENU93267.1 RPE30045.1

WP 123775416.1

WP 081407343.1 ENX09243.1

WP 009510197.1

WP 081401650.1 EPF93277.1 ESK38007.1

WP 081403844.1

91

53

44

64

56

56

34

35

27

99 80

99

84

30 83

99

42

77

52

99

91

34

63

99

54

26

36

39

80

56

99

99

80

85

37

99

99

98

90

98

47

99

9

90

96

99

34

31

38

61 99

48

38

65

87

99 78

83

19

58

39

23

72

30

54

32

45

35

35

41

99

99

99

99

31

25

9 96

62

61

79 99

72 97

53

42

99

89

99

85

57

68

47

65

99

88

64

32

99

46

99

55

44

79

99

99

56

71

99

14

54

83 68

56

89

31

99

67

80

62 99

99

46

84

46

64

78

55

48

81

64

44

53

62

42

57

98

91

15 99

96

64

62

33

60

99

14

29

99

3

88

98

45

98

9

99

99

73

5

91

48

16

99

26

79

83

94

91 76

58

70

98

69 86

99

WP 081401950.1

WP 081402010.1

WP 081401904.1

WP 081406930.1

WP 081401814.1

WP 101235492.1 ENU21674.1

WP 032864351.1 ENV10142.1

WP 130160754.1

WP 121929032.1

WP 081398975.1

WP 081400731.1 ENW20351.1 WP 150024750.1 EEH68243.1 ENW22118.1 WP 017395615.1 HAK15860.1 OFW44053.1 ENV64477.1 EPR86420.1 ENV51313.1 HCM30849.1 WP 034683297.1

WP 045793218.1

WP 131388464.1

WP 034425615.1

WP 131382055.1 ENV47184.1

WP 116727941.1

WP 131375671.1

WP 131312428.1

WP 111895072.1 ESK38773.1

WP 040331973.1

WP 087620370.1 SNX45987.1

WP 097079742.1 BBF76985.1

WP 049174423.1 ENV78217.1

WP 130803940.1

WP 120369847.1

WP 004923335.1

WP 048765277.1

WP 088458630.1

WP 132070613.1

WP 133747326.1

WP 056482157.1

WP 092708471.1 ENW01981.1 WP 133971229.1 EOQ60907.1

WP 111855215.1

WP 009391329.1

WP 057106088.1

WP 033849065.1 EXA67995.1

WP 100290049.1

WP 019458531.1

WP 077168837.1

WP 071210169.1 SSM90614.1 ETR82976.1

WP 069036603.1

MurG from Acinetobacter baumannii WP 000132435.1

WP 000132433.1

WP 140953803.1 SPL68923.1

WP 121972497.1 ENV31818.1

WP 020846716.1 EPF81552.1

WP 040330960.1

WP 067558430.1 TCM68745.1

WP 139853478.1

WP 067663080.1

WP 086189828.1

WP 086205195.1

WP 086204224.1

WP 065993089.1

WP 111858988.1

WP 130072389.1

WP 107009456.1 ENV89452.1

WP 010590909.1 ENV19691.1

WP 016539102.1

WP 056517158.1

WP 105713898.1 ENU60003.1 ENV17748.1

WP 010112319.1

WP 120383482.1

WP 130112832.1

WP 130116870.1

WP 130131070.1

WP 087512016.1

WP 087549744.1

WP 044738880.1 EOR02599.1

WP 131325623.1

WP 131324192.1

WP 116759136.1

WP 131264210.1

WP 142769955.1 SCC72224.1

WP 092720068.1

WP 053579255.1

WP 039620241.1

WP 092689979.1

WP 086184053.1 SDY40554.1

WP 086165337.1 ENU21062.1 SFT09247.1

WP 074947103.1

WP 086186928.1

WP 131366157.1

WP 067869251.1

WP 131216843.1

WP 131384047.1

WP 131267760.1

WP 131317157.1

WP 131288345.1

WP 131271374.1

WP 086193803.1

WP 067725439.1

WP 067668390.1

WP 086211587.1

WP 068910292.1

WP 005006719.1

WP 068885870.1

WP 120365498.1

WP 120402735.1

WP 086177223.1

WP 067761632.1

WP 078193220.1

WP 078190577.1

WP 111884460.1

WP 062031947.1 OHC23638.1 SNU14554.1 ENV74306.1 ENU37982.1

WP 114392648.1

WP 087526678.1

WP 110974620.1

WP 092820539.1

WP 131274197.1

WP 086196542.1

WP 086174703.1

WP 086199014.1

WP 104500254.1

WP 005180817.1

WP 104852726.1

WP 019837075.1

WP 070155856.1

WP 122900661.1

WP 054580793.1

WP 086180164.1

WP 092766516.1

WP 035265837.1

WP 067727838.1

WP 004813763.1

WP 008305759.1

WP 005220556.1

WP 099337732.1

WP 034588633.1

WP 125279159.1

WP 100534533.1

WP 100354939.1

WP 100356984.1 ENW25488.1 EAM8864034.1


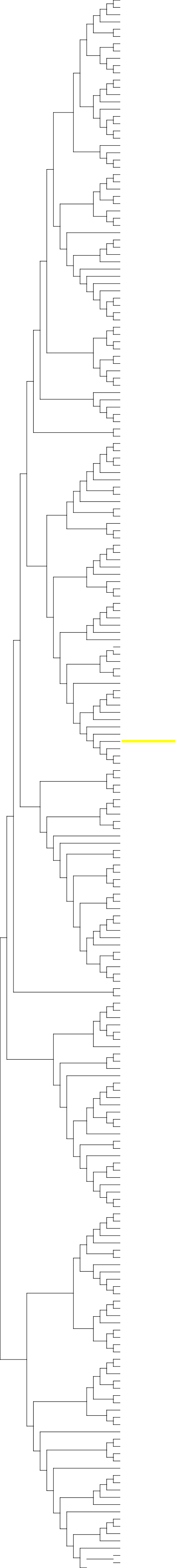


77

|  | | | | 84 | WP 148335071.1 |
| --- | --- | --- | --- | --- | --- |
|  |  |  |  |  | WP 005245707.1 |
| 72 | 61 |  |  |  | RDC53559.1  WP 005250316.1 |
|  |  | 59 |  |  | WP 071850281.1 |
|  |  |  | 37 |  | WP 044111339.1 |
|  |  |  |  | 40 | WP 121979730.1 |


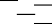


**Fig. S13.** Phylogenetic analysis of MurG using Minimum Evolution Method

75

61

50

65

37

77

28

99

97

99

20

97

69

78

39 27

49

99

47

41

32

99

52

27

71

47

99

59

56

86

99

61

77

97

88 47

90

31

49

46

50

48

20

19

26

99 87

99

96

27 82

99

44 79

58

99

35

28

62

99

40

25

33

43

83

96

63

99

82

79

99

91

98

48

99

29 97

99

99

68

93

84

54

KKW76424.1 WP 081407495.1

WP 081401574.1 OJU89657.1

WP 081408195.1

WP 081399778.1 ESK55389.1

WP 018679367.1 ENW06013.1 ENW07184.1 WP 034710633.1

WP 081399858.1

WP 087547617.1

WP 087537056.1

WP 087543583.1 ENV38228.1 KXO80407.1 HBO71187.1 PRD19162.1

WP 019385625.1 ENU33449.1 ENU35076.1

WP 081399900.1

WP 081399063.1 RPE30045.1

WP 123775416.1 ENU93267.1 KYQ85260.1

WP 081410353.1 ENX09243.1

WP 009510197.1

WP 081407343.1

WP 081401650.1 ESK38007.1

WP 081403844.1 EPF93277.1

WP 081401950.1

WP 081402010.1

WP 081401904.1

WP 101235492.1 ENU21674.1 ENV10142.1

WP 081401814.1

WP 032864351.1

WP 081406930.1

WP 130160754.1

WP 121929032.1

WP 081398975.1

WP 081400731.1 ENW20351.1 WP 150024750.1 EEH68243.1 ENW22118.1 WP 017395615.1 HAK15860.1 ENV64477.1 OFW44053.1 EPR86420.1 ENV51313.1 HCM30849.1 WP 034683297.1

WP 034425615.1

WP 131382055.1 ENV47184.1

WP 116727941.1

WP 045793218.1

WP 131388464.1

WP 131375671.1

WP 131312428.1

WP 111895072.1 ESK38773.1

WP 040331973.1 BBF76985.1

WP 049174423.1 ENV78217.1

WP 120369847.1

WP 130803940.1

WP 004923335.1

WP 048765277.1

WP 088458630.1 EOQ60907.1

WP 111855215.1

WP 009391329.1

WP 057106088.1

65

89

35

36

39

99

99

63

58

43

28

35

53

37

82

52

44

49

40

32

44

99

98

99

99

99

99

35

53 95

69

48

99

13

|  |  |  | 39 |  | WP 086204224.1 |
| --- | --- | --- | --- | --- | --- |
|  |  |  |  |  | WP 086189828.1 |
|  |  |  |  | 97 | WP 065993089.1 |
|  |  |  | 64 |  | WP 111858988.1 |
|  |  |  |  |  | WP 130072389.1 |
|  |  |  |  | 99 | WP 107009456.1 |
|  |  |  |  | 91 | ENV89452.1 |
|  |  |  | 99 |  | WP 010590909.1 |
|  |  |  |  |  | ENV19691.1 |
|  |  |  |  | 53 | WP 016539102.1 |
|  |  |  | 69 |  | WP 056517158.1 |
|  |  | 51 |  |  | WP 105713898.1 |
|  | 99 |  |  |  | ENU60003.1 |
|  |  |  |  |  | ENV17748.1 |
| 57 |  |  |  | 90 | WP 010112319.1 |
|  |  |  |  |  | WP 120383482.1 |
|  |  | 35 |  |  | WP 130112832.1 |
|  |  |  | 99 |  | WP 130116870.1 |
|  |  |  |  | 46 | WP 130131070.1 |
|  |  |  |  |  | WP 087512016.1 |
|  |  |  |  | 99 | WP 087549744.1 |
|  |  |  |  | 67 | WP 062031947.1 |
|  |  |  | 70 |  | OHC23638.1 |
|  |  | 46 |  |  | SNU14554.1 |
|  | 99 |  |  |  | ENV74306.1 |
|  |  |  |  | 67 | ENU37982.1 |
|  |  |  |  |  | WP 114392648.1 |
| 16 |  |  | 22 |  | WP 087526678.1 |
|  |  |  |  | 36 | WP 110974620.1 |
|  |  |  |  | 99 | WP 104500254.1 |
|  |  |  |  |  | WP 005180817.1 |
|  |  |  |  | 99 | WP 086174703.1 |
|  | 61 |  |  |  | WP 086199014.1 |
|  |  | 98 |  |  | WP 086196542.1 |
|  |  |  | 97 |  | WP 092820539.1 |
|  |  |  |  | 99 | WP 131274197.1 |
|  |  |  |  | 58 | WP 044738880.1 |
|  |  |  | 58 |  | EOR02599.1 |
|  |  | 52 |  |  | WP 131325623.1 |
|  | 99 |  | 99 |  | WP 131324192.1  WP 116759136.1 |
|  |  |  |  | 52 | WP 131264210.1 |
|  |  |  |  |  | WP 142769955.1 |
|  |  |  |  | 57 | WP 086193803.1 |
|  |  |  | 63 |  | WP 067725439.1 |
|  |  | 83 |  |  | WP 131271374.1 |
|  | 52 |  |  |  | WP 131288345.1 |
|  |  |  |  |  | WP 131317157.1 |

WP 033849065.1

WP 132070613.1

WP 133747326.1

WP 056482157.1

WP 092708471.1 ENW01981.1 WP 133971229.1

WP 100290049.1

WP 019458531.1

WP 077168837.1

WP 071210169.1 SSM90614.1 EXA67995.1 ETR82976.1

WP 069036603.1

MurG from Acinetobacter baumannii WP 000132435.1

WP 000132433.1

WP 140953803.1

WP 087620370.1 SNX45987.1

WP 097079742.1 SPL68923.1

WP 121972497.1 ENV31818.1

WP 020846716.1 EPF81552.1

WP 040330960.1

WP 067558430.1 TCM68745.1

WP 139853478.1

WP 067663080.1

WP 086205195.1

95

47

54

50


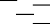


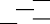


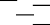


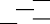


7


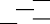


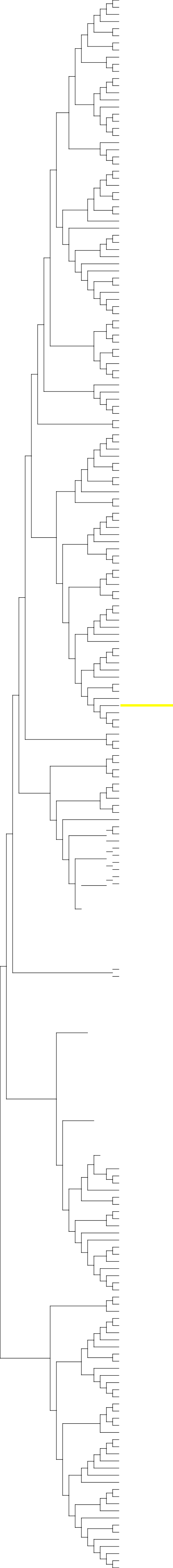
25

52

90

46

99

99

68

86

99

46

66 84

68

99

76

73

67

52

69

99

84

59

48

64

89

34

4 70

97

81

99

96

97

46

7

38

99

76

80

88

73

18

34

23

74

98

89

90

79

88

85

55

59

52

27

39

WP 131384047.1

WP 131267760.1

WP 131216843.1

WP 131366157.1

WP 067869251.1 SCC72224.1

WP 092720068.1

WP 053579255.1

WP 039620241.1

WP 086186928.1 SFT09247.1

WP 074947103.1 ENU21062.1

WP 086165337.1 SDY40554.1

WP 092689979.1

WP 086184053.1

WP 019837075.1

WP 070155856.1

WP 104852726.1

WP 067668390.1

WP 086211587.1

WP 068910292.1

WP 005006719.1

WP 068885870.1

WP 120365498.1

WP 120402735.1

WP 086177223.1

WP 067761632.1

WP 078193220.1

WP 078190577.1

WP 111884460.1

WP 054580793.1

WP 086180164.1

WP 092766516.1

WP 035265837.1

WP 122900661.1

WP 004813763.1

WP 008305759.1

WP 005220556.1

WP 099337732.1

WP 034588633.1

WP 125279159.1

WP 067727838.1

WP 100534533.1

WP 100354939.1

WP 100356984.1 ENW25488.1 EAM8864034.1 WP 148335071.1

WP 005245707.1 RDC53559.1

WP 005250316.1

WP 071850281.1

WP 044111339.1

WP 121979730.1

**Fig. S14.** Phylogenetic analysis of MurG using Maximum Parsimony Method

96

68

63

65

80

WP 101235492.1 ENU21674.1

WP 081401814.1

WP 081406930.1 ENV10142.1


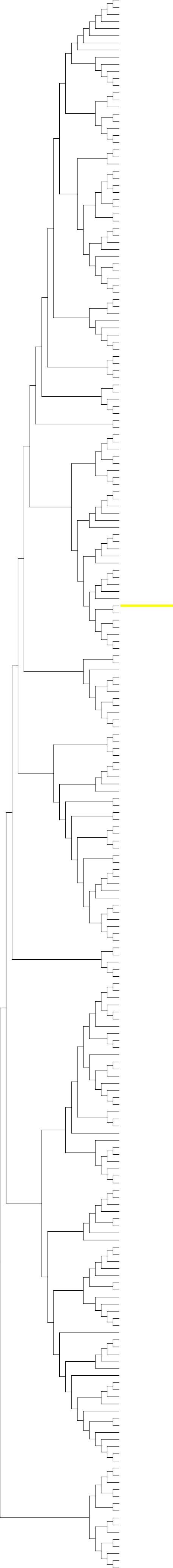


52

91

50

38

39

46

60

99

25

8

33

60 99

30

58

58

7

55

26

61

35

88

16

1

3

19

70

61

71

99

78

35

56

99

83

99

61

86

79

87

89

97

87

91

95

28

31

22

21

69

99

83

25 87

78

33

86

40

99

62

55

63

99

29

99

72

80

94

54

97

87

87

99

63

61

99

85

99

99

77

39

73

99

81

80

82

99

51

65

96

55

88

52

22

28

47

55

76

87

95

64

36

61

71

99

90

43

16

99

67

86

7

45

84

99

99

99

99

42

30

95

98

56

93

56

99

77

82

81

83

47 99

98

46 99

59

99

85

99

32

99

99

99

51

32

39

36

47

97

48 79

49

98

95

99

99

81

81

85

99

86

97

74

99

76

93

72

61

57

47

37

48

92

97

49

41

53

50

74

50

78

95

86

99

99

79

95

82

92

87

98

85

98

53

66

65

55

28

34

99

82

64

34

99

99

98

6

99

99

12

31

36

78

WP 032864351.1

WP 081401904.1

WP 081402010.1

WP 081401650.1

WP 081401950.1 ESK38007.1 EPF93277.1

WP 081403844.1 ENX09243.1

WP 009510197.1

WP 081407343.1 RPE30045.1

WP 123775416.1 ENU93267.1 KYQ85260.1

WP 081410353.1 ENW07184.1 WP 034710633.1 ENW06013.1 KKW76424.1 WP 081407495.1 OJU89657.1

WP 081401574.1

WP 081408195.1

WP 081399778.1 ESK55389.1

WP 018679367.1

WP 081399858.1

WP 087547617.1

WP 087537056.1

WP 087543583.1 ENU33449.1 PRD19162.1

WP 019385625.1 HBO71187.1 KXO80407.1 ENV38228.1 ENU35076.1

WP 081399900.1

WP 081399063.1 ENW22118.1 WP 017395615.1 EEH68243.1 ENW20351.1 WP 150024750.1

WP 130160754.1

WP 121929032.1

WP 081398975.1

WP 081400731.1 EPR86420.1 ENV51313.1 HAK15860.1 ENV64477.1 OFW44053.1 HCM30849.1 WP 034683297.1 BBF76985.1

WP 049174423.1 ENV78217.1

WP 130803940.1

WP 120369847.1

WP 004923335.1

WP 048765277.1

WP 088458630.1 ENW01981.1 WP 092708471.1

WP 056482157.1

WP 133747326.1

WP 132070613.1

WP 133971229.1 EOQ60907.1

WP 009391329.1

WP 111855215.1

WP 033849065.1

WP 057106088.1

WP 100290049.1

WP 019458531.1 SSM90614.1

WP 071210169.1

WP 077168837.1

MurG from Acinetobacter baumannii WP 000132435.1

EXA67995.1 ETR82976.1

WP 140953803.1

WP 000132433.1

WP 069036603.1 ESK38773.1

WP 040331973.1

WP 111895072.1 ENV47184.1

WP 131382055.1

WP 116727941.1

WP 045793218.1

WP 131388464.1

WP 034425615.1

WP 131375671.1

WP 131312428.1 SPL68923.1

WP 121972497.1 ENV31818.1

WP 020846716.1 EPF81552.1

WP 040330960.1

WP 067558430.1 TCM68745.1

WP 139853478.1

WP 086205195.1

WP 086204224.1

WP 086189828.1

WP 067663080.1

WP 065993089.1

WP 111858988.1

WP 130072389.1

WP 107009456.1

WP 010112319.1

WP 120383482.1 ENU60003.1

WP 056517158.1

WP 105713898.1

WP 016539102.1 ENV17748.1 ENV89452.1

WP 010590909.1 ENV19691.1

WP 130112832.1

WP 130116870.1

WP 130131070.1

WP 087512016.1

WP 087549744.1

WP 087620370.1 SNX45987.1

WP 097079742.1

WP 131366157.1

WP 067869251.1

WP 131216843.1

WP 131271374.1

WP 086193803.1

WP 067725439.1

WP 131288345.1

WP 131317157.1

WP 131384047.1

WP 131267760.1

WP 086186928.1 SFT09247.1

WP 074947103.1 ENU21062.1

WP 086165337.1

WP 086184053.1 SDY40554.1

WP 092689979.1

WP 053579255.1 SCC72224.1

WP 092720068.1

WP 039620241.1

WP 142769955.1

WP 044738880.1 EOR02599.1

WP 131325623.1

WP 131324192.1

WP 116759136.1

WP 131264210.1 SNU14554.1

WP 062031947.1 OHC23638.1 ENV74306.1 ENU37982.1

WP 114392648.1

WP 087526678.1

WP 110974620.1

WP 067668390.1

WP 086211587.1

WP 068910292.1

WP 005006719.1

WP 068885870.1

WP 120365498.1

WP 120402735.1

WP 086177223.1

WP 067761632.1

WP 078193220.1

WP 078190577.1

WP 111884460.1

WP 067727838.1

WP 005220556.1

WP 008305759.1

WP 004813763.1

WP 099337732.1

WP 034588633.1

WP 125279159.1

WP 100534533.1

WP 100354939.1

WP 100356984.1 ENW25488.1 EAM8864034.1 WP 148335071.1

WP 005245707.1 RDC53559.1

WP 005250316.1

WP 071850281.1

WP 044111339.1

WP 121979730.1

WP 092820539.1

WP 131274197.1

WP 086196542.1

WP 086174703.1

WP 086199014.1

WP 104500254.1

WP 005180817.1

WP 019837075.1

WP 070155856.1

WP 104852726.1

WP 092766516.1

WP 035265837.1

WP 122900661.1

WP 054580793.1

WP 086180164.1

**Fig. S15.** Phylogenetic analysis of MurG using Maximum Likelihood Method

47

21

26

19

44

WP 101235492.1 ENU21674.1 ENV10142.1

WP 032864351.1

WP 081401814.1


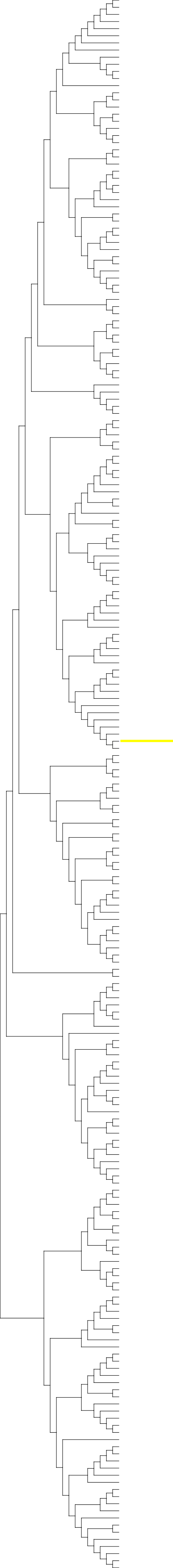
48

28

23

60 41

93

49

41

94

45

62

57

79

21 30

58

82

86

36

40

81

27 63

29

96

18

20

77

72

20 46

64

19

35

49 34

36

85

94

49

62

89

89

69

25 81

93

38

51

97

34

52

73

99

57

99

19

99

61

19

38

35

63

89

99

76

86

17

99

31

99

89

86

98

65

97

68

28

26

26

28

40

94

35

71

94

49

80 81

58

21

35 16

20

47

22

34

37

59

35

42

99

98

99

99

10

38

40 84

31

53

65

95

64

94

59

27

99

86

56

56

52

47

63 91

84

41 99

32

99

45

99

81

42

65

97

95

54

7 79

99

71 64

67

54

85

28

92

76

87

99

44 38

43

93 81

35

43

57

54

96

85

71

42

98

9

99

2 99

75

80

18

29

99

46

58

54

33

0

37

68

94

53

40

46

54

73

4

48

62

93

87

99

95

13

69

91

82

85

74 27

78

98

53

77

99

87

58

64

59

55

26

36

WP 081406930.1

WP 081401904.1

WP 081402010.1

WP 081401950.1

WP 081403844.1 EPF93277.1 ESK38007.1

WP 081401650.1 ENX09243.1

WP 009510197.1

WP 081407343.1 RPE30045.1

WP 123775416.1 ENU93267.1 KYQ85260.1

WP 081410353.1 ENW07184.1 WP 034710633.1 ENW06013.1 KXO80407.1 HBO71187.1 PRD19162.1

WP 019385625.1 ENV38228.1 ENU33449.1 ESK55389.1

WP 018679367.1

WP 081399858.1

WP 087547617.1

WP 087537056.1

WP 087543583.1

WP 081408195.1

WP 081399778.1 OJU89657.1

WP 081401574.1 KKW76424.1 WP 081407495.1

WP 081399063.1 ENU35076.1

WP 081399900.1

WP 130160754.1

WP 121929032.1

WP 081398975.1

WP 081400731.1 ENW20351.1 WP 150024750.1 EEH68243.1 ENW22118.1 WP 017395615.1 HAK15860.1 OFW44053.1 ENV64477.1 EPR86420.1 ENV51313.1 SNX45987.1

WP 097079742.1

WP 087620370.1 HCM30849.1 WP 034683297.1

WP 045793218.1

WP 131388464.1

WP 034425615.1

WP 131382055.1 ENV47184.1

WP 116727941.1

WP 131375671.1

WP 131312428.1

WP 111895072.1 ESK38773.1

WP 040331973.1

WP 048765277.1

WP 088458630.1

WP 004923335.1

WP 130803940.1

WP 120369847.1 ENV78217.1 BBF76985.1

WP 049174423.1

WP 132070613.1

WP 133747326.1

WP 056482157.1 ENW01981.1 WP 092708471.1

WP 133971229.1 EOQ60907.1

WP 111855215.1

WP 009391329.1

WP 033849065.1

WP 057106088.1

WP 100290049.1

WP 019458531.1

WP 077168837.1 SSM90614.1

WP 071210169.1 EXA67995.1 ETR82976.1

WP 069036603.1

WP 000132433.1

WP 140953803.1

MurG from Acinetobacter baumannii WP 000132435.1

SPL68923.1

WP 121972497.1 ENV31818.1

WP 020846716.1 EPF81552.1

WP 040330960.1

WP 067558430.1 TCM68745.1

WP 139853478.1

WP 086189828.1

WP 067663080.1

WP 086205195.1

WP 086204224.1

WP 065993089.1

WP 111858988.1

WP 130072389.1

WP 107009456.1

WP 010112319.1

WP 120383482.1

WP 016539102.1

WP 056517158.1

WP 105713898.1 ENU60003.1 ENV17748.1 ENV89452.1

WP 010590909.1 ENV19691.1

WP 130116870.1

WP 130112832.1

WP 130131070.1

WP 087512016.1

WP 087549744.1

WP 044738880.1 EOR02599.1

WP 131325623.1

WP 131324192.1

WP 116759136.1

WP 131264210.1

WP 142769955.1

WP 039620241.1 SCC72224.1

WP 092720068.1

WP 053579255.1 SDY40554.1

WP 092689979.1

WP 086184053.1

WP 086165337.1 ENU21062.1 SFT09247.1

WP 074947103.1

WP 086186928.1

WP 131366157.1

WP 067869251.1

WP 131216843.1

WP 131384047.1

WP 131267760.1

WP 131317157.1

WP 131288345.1

WP 131271374.1

WP 086193803.1

WP 067725439.1

WP 092820539.1

WP 131274197.1

WP 086196542.1

WP 086174703.1

WP 086199014.1

WP 104500254.1

WP 005180817.1

WP 104852726.1

WP 019837075.1

WP 070155856.1

WP 122900661.1

WP 054580793.1

WP 086180164.1

WP 092766516.1

WP 035265837.1 SNU14554.1

WP 062031947.1 OHC23638.1 ENV74306.1 ENU37982.1

WP 114392648.1

WP 087526678.1

WP 110974620.1

WP 067668390.1

WP 086211587.1

WP 068910292.1

WP 005006719.1

WP 068885870.1

WP 120365498.1

WP 120402735.1

WP 086177223.1

WP 067761632.1

WP 078193220.1

WP 078190577.1

WP 111884460.1

WP 067727838.1

WP 005220556.1

WP 008305759.1

WP 004813763.1

WP 099337732.1

WP 034588633.1

WP 125279159.1

WP 100534533.1

WP 100354939.1

WP 100356984.1 ENW25488.1 EAM8864034.1 WP 148335071.1

WP 005245707.1 RDC53559.1

WP 005250316.1

WP 071850281.1

WP 044111339.1

WP 121979730.1

**Fig. S16.** Multiple Sequence Alignment Eight Mur Family Proteins from *Acinetobacter baumannii*

Multiple Sequence Alignments of Mur family proteins

# There are 7 groups

Start of Multiple Alignment Aligning...

Group 1: Delayed

Group 2: Delayed

Group 3: Delayed

Group 4: Delayed

Group 5: Delayed

Group 6: Delayed

Group 7: Delayed

Alignment Score -6084

CLUSTAL-Alignment file created

CLUSTAL 2.1 multiple sequence alignment

MurE ---MTVSFQEIHPIEIDAQWPQQPFHGFSLDSRKVETGQIFIALTSYSQPEKTRTFAEAA MurF --MHTSTTSTVPLEPWTAQQLQQATQGYWHKDQIPQTEIKRILTDSRHAESGDAFLALKG MurC MSPTTAANQAKKLIKVPEMRRIKHIHFVGIGGAGMCGIAEVLANQGYKISGSDIKASKTT MurD --MINCGAKMLIQRGGLKVVAGLGISGVSAVNFLHEQGYQVAVTDSRPTPPG H

MurG ----MTDSQQSKPKHVMMMAAGTGGHVFPALAVAKQLQQQGCQVSWLATPTG--------

MurA --MDKFLITGGVKLEGEVRISGAKNAALPLLAAMILADSPITLTNVPNLKDVN-------

MurB ----MFKNRFFNTMQIQNQVQLKPFNTLSLDVTASHYTKVKSIED---------------

MraY MLLWLFEQLAGYHSSFQVVRYLTLRSLLSVLTSLTIGLVLGPIMIRKLQALKYG------

MurE LANGALAVISETELGVANEWVCSDVRQRMGDWQKRYLQQADVVKPLRIIAVTGTNGKTTI MurF ERFDAHNFVAQ VVANGCQVAIVERPIDAEIAQLVVADTRLALGQLGAYRREQNAQL

MurC QQLEENGIKVYIGHEAENIKNANVLVVSTAIDPENPEVKAAIEQRIPIVRRAEMLGELMR MurD DQIPAGVKTSFGQLDQELLLQAEEIILSPGLAPQLPEIQAAIAKGISVVGDIQLLRRATD MurG ------------------------MENRLLKDQNIPIYQIDIQG----------------

MurA -----------------------TLVKLIGGLGVTISYENDTVK----------------

MurB -------------------------IEEALAFAKEHELNVLVLSG---------------

MraY -------------------------QAVSSFAPENHAKKMGTPT----------------

MurE SRLIAELISSQQQRCAVMGTTGNGILPNLTPSTHTTLDALQLQNALHDYAKQGATFASLE MurF KVIALTGSSGKTTTKEMLGSILSRLAPTLITRGNLNNDLGVPMMLLELRKEHQYAVMELG MurC YRHGIAVAG THGKTTTTSLLTTMLAEENLDPTYVIGGLLNSTGVNAALGESRFI

MurD VPIVAITGS NAKSTVTTLIGLMAKDAGKKVAVGGNLGRPALDLLKDQPELLV

MurG VRGNGVIRKLAAPFKILKATFSAMRYMKQLKVDAVAGFGGYV

MurA ADTSTLDNQFAPYELVKTMRASILVLGPLLARYGNAKVSLP

MurB GSNMLLPQQINALVIHLDIQGIDVLSEDQDFIRVKVGAGQVW

MraY MGGILILLSIGISTLLWADLSNPYVWIVLGVMVVFGAVGWA

MurE ASSHGLEQGRLNGCDIEIAVYSNLSRDHLDYHGTLEAYAEAKARLFQFNSLKVAVINLDD MurF ANHQGEIDYTSKIVQPHVAGILNIGTAHLGEFGGRDGICRAKSEIYRHILPQGVAIVP-- MurC VAEADESDASFLYLQPMAAIVTNIDADHMDTYEGSFDKLKDTFVQFLHNLPFYGLAVVCG MurD LELSSFQLETTSHLNAEVAVVLNMSEDHLDRHGNMLGYHQAKHRIFQGAKKVVFNRDD-- MurG AGPGGLAARLLGIPVLIHEQNAVAGFTNAQLSRVAKVVCEAFPNTFPASEKVVTTGNP-- MurA GGCAIGSRPVDQHLKALEALGAHIEVENGYVHATVDGRLKGGEVVFDMVTVGGTENILMA MurB HDFVLYTTKQNWFGLQNLALIPGLVGASPVQNIGAYGVEVGEFIESVQVYDRLLKQTG-- MraY DDWIKIRYKDNAGLPARKKFFWTSVASLGAGIALYLIATQQSNAEYTANMLDLLIPFFKN

MurE AHADLMIKSAQNNPAQPKILTYSLTQNTADYYITDLSYSLAGATFNLVSQ-QGSFAVESP MurF -QQDDFTAEIREAAKSHQIMSFGAGG DVFATEIELLPQSANFQLHTP-QGSSFVRLP

MurC ---DDANIREILPRVGRPVITYGFNEDN-DIRAIDVEQDGMRSHFTVLRKGREPLRLTIN MurD ALSRPLVPDTTPMQSFGLNAP--DLNQYGVLRDADGTLWLARGLQRLIKSSDLY

MurG VRREITDILSPKWRYDEREQAGKPLNILIVGGSLGAKALNERLPPALKQLEVP

MurA AALADGVTTIRNAAREPEITDLAQMLIKMGAKIEGLDTDTLVVTGVESLHGCEYAVVADR MurB SISAADCHFSYRHSIFKDDPARYIITHVTFKLLKQANLKLNYGDLKQAVGD

MraY LSIPLSIVPLGLAFIVFTYLVINGASNAVNLTDGLDGLAIMPVVMV

MurE LLGHFNVENLIAALIAAEQAGFDLQALVNFVPKLIGAPGRMQVIRDDER LFVVD-Y

MurF FAGEHNVQNATAAVAFALALGVSLEDIVKGLEQAQGAKGRLNFIQKTPH LFIDDTY

MurC QPGLHNVLNALAAIGVATDEGVSDEAISRALKGFSGVGRRFQVQGEFELGEGNVKLVDDY MurD IQGMHNVANALACLALGEAIGLPMESMLETLKQFKGLEHRCEYVKTVHD VRYY

MurG LNIFHQCGQQQVEATQALYADAPANLTVQVLPFIEDMAKAYSEADLIICR----------

MurA IETGSYLAAAAITGGRVKTTHTDPSLLEAVLDKFEEMGAEVTRGDDWIEL----------

MurB NLTAENLQNQVIHIRQSKLPDPKEYPNVGSFFKNPIVNTQEFER----------------

MraY ATGLGVFAYLSGDIRFANYLHIPYVKYTSELVVICSAMIGAGLAFLWYN-----------

.

MurE AHTPDALIQVLKTLKRHVSNQLWAVFGCGGDRDRGKRPLMTQAALDGANPVILTSDNPRT MurF NANPTSMRAAAQVLLQQNGIKVMVMGDIGELGDSSWQEHHDLGRDLAELPLDHIVAVG-- MurC GHHPKEVEATIKAARQSHPDRRLVMLFQPHRYSRTRDCFDDFIEVLSQVDQLLLLEVYP- MurD NDSKGTNVGATLAAIDGLGAAIEVKKGKVALILGGQGKGQDFGPLRSSIEKYAKVVVLIG MurG AGALTVTEVATAGVAAVFVPLPIAVDDHQTANAKFLADVGAAKICQQSTMTP-

MurA -DMLGKRPKAVSFRTLPHPEFPTDMQAQIMAVNAIGRGFATISETIFENRFMHVPELSR- MurB LIAQFSTIPHYPQANGNVKIAAGWLIDQAGWKGKQLGVVGMFHKQALVLVNYAN

MraY AHPAQVFMGDVGALALGAMLGTIAVMVRQEIVFAIMGGVFVMEAVSVFLQIGS-

MurE EDPEQIFADMKQGINFSGHRMHEIHDRREAIKFVAEQAQAGDIVVIAGKGHENYQEINGV MurF ----QFASAALEGAGLHSTKLKAFQTQAEALPFLINLIQT HQPQSMSFLFKGSRF

MurC ------AGEKPIVGADSRTLARSIRLRGQVEPILIDPVEGN LQNIMQNVLQPNDLL

MurD EDAPVIEQAIQGATKILHAATLKEAVELCQRETQAEDVVLLSPACASFDMFKSYN

MurG -----------EVLNQLFTTLMNRQLLTEMAVKARQHAQPN-------------------

MurA MGANIQVEGHDAVVTGVEKLQAAPVMATDLRASFSLVLAALVAEGDTLIDRI

MurB -----------ASLADVKKTYQAVQHDVEQRFQIMLEPEP V

MraY ----------LRMRNKRVFLMAPLHHHYEKQGWKETQVVIR FWI

:

MurE RHWFDDVVEVRSAIDAQHHTVDAAYPAQ

MurF THMETLMADLMEKL--------------

MurC LTQGAGNVGAISVELAQHHLYVK-----

MurD DRGQQFVACVNSLV--------------

MurG --ATQHVVDLIQKM--------------

MurA YHIDRGYEHVEEKLQGLGAKIKRVS---

MurB LYNNLGLIENHTE---------------

MraY ITIMLVVLGLMTLKLR------------

**Fig. S17:** Structure validation reports for MurB, MurE and MurG from different species

**MurB structure from *A. calcoaceticus* evaluation results**


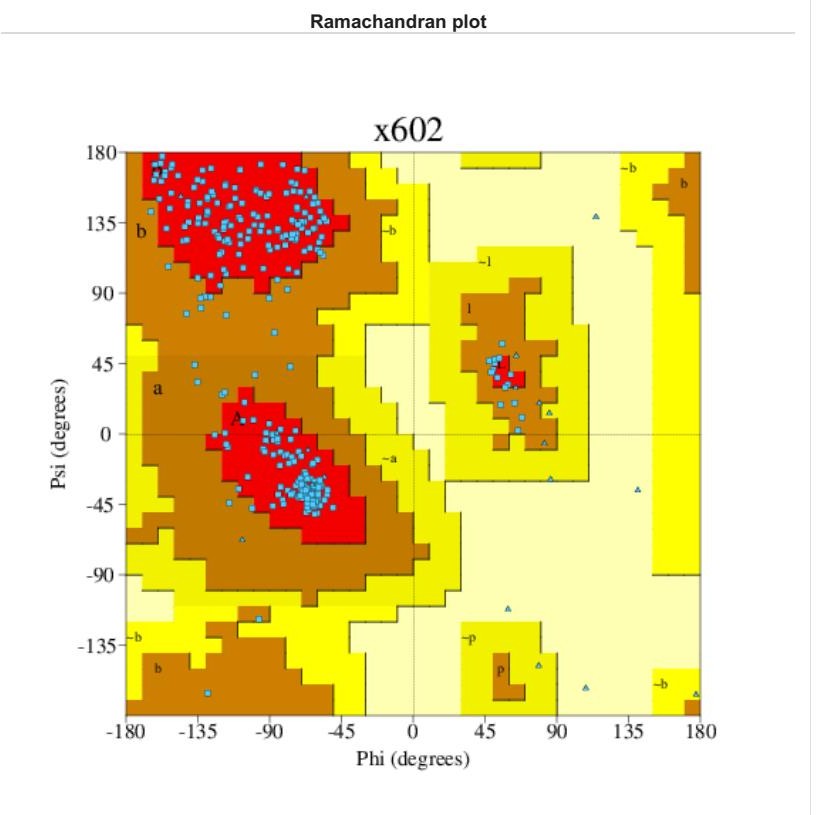


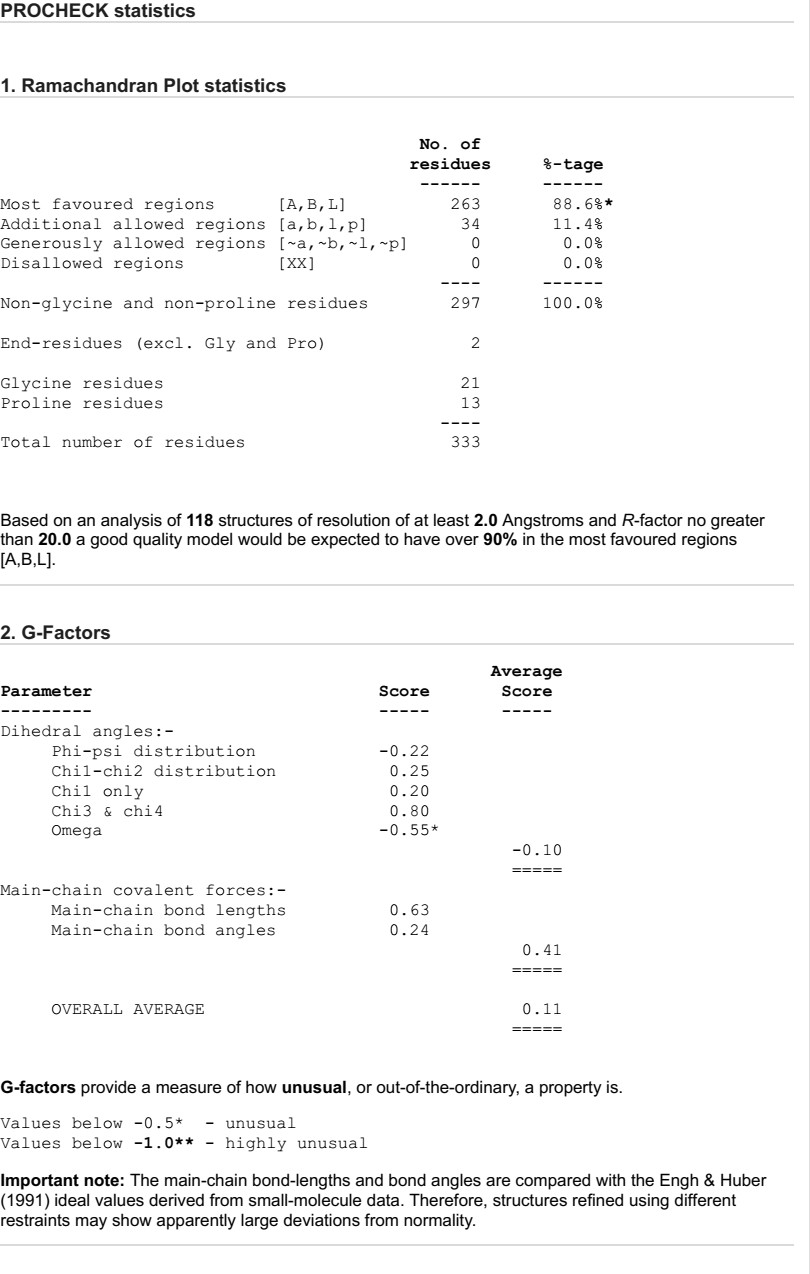


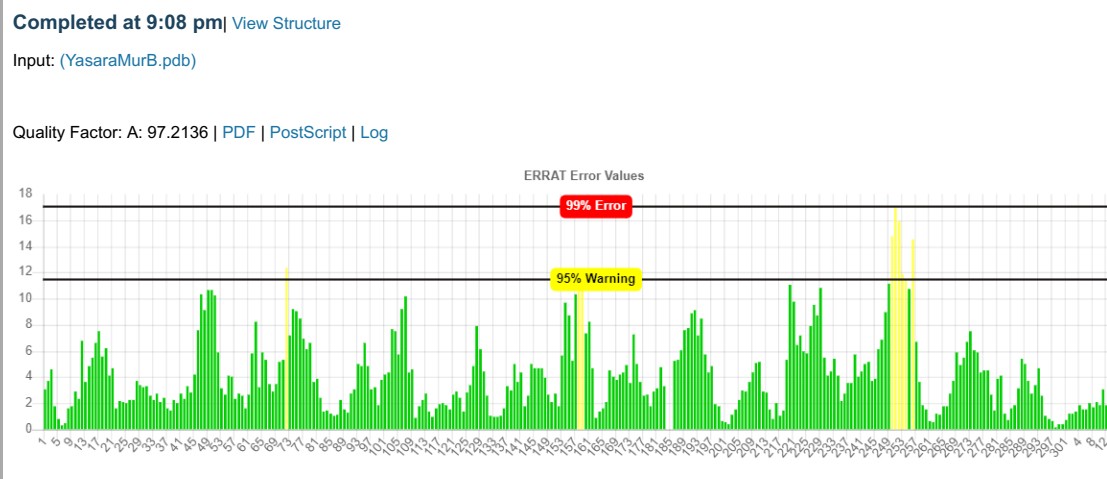


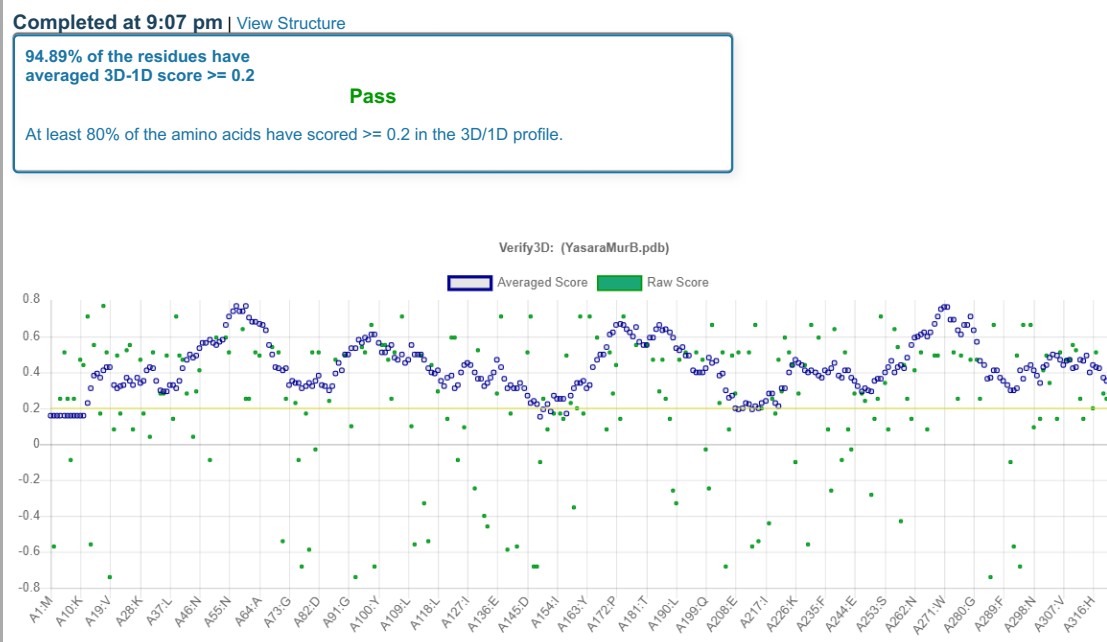


**MurE structure from *A. seifertii* evaluation results**


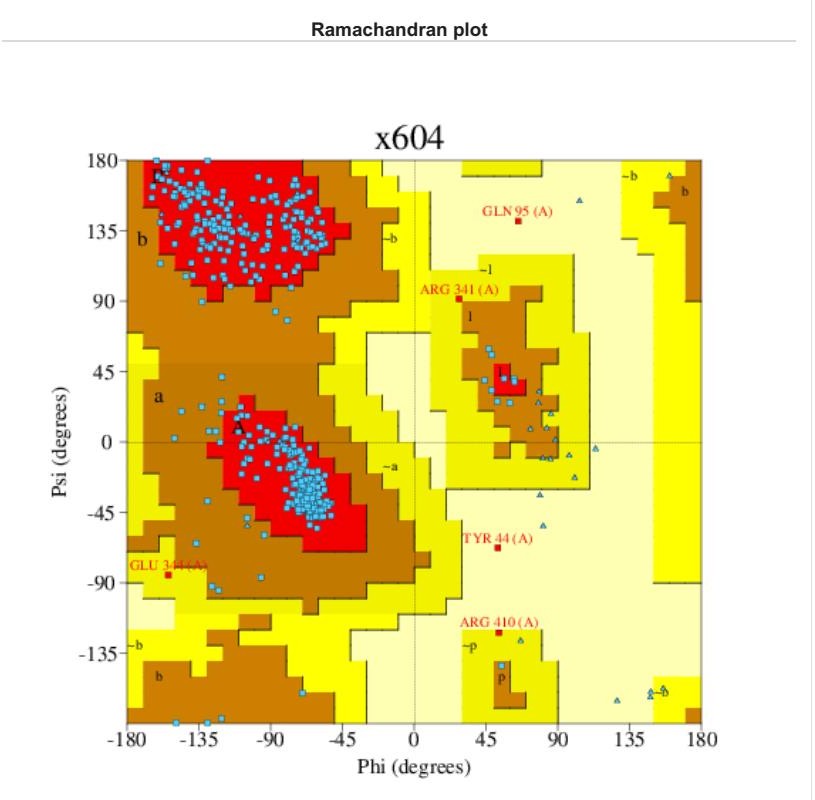


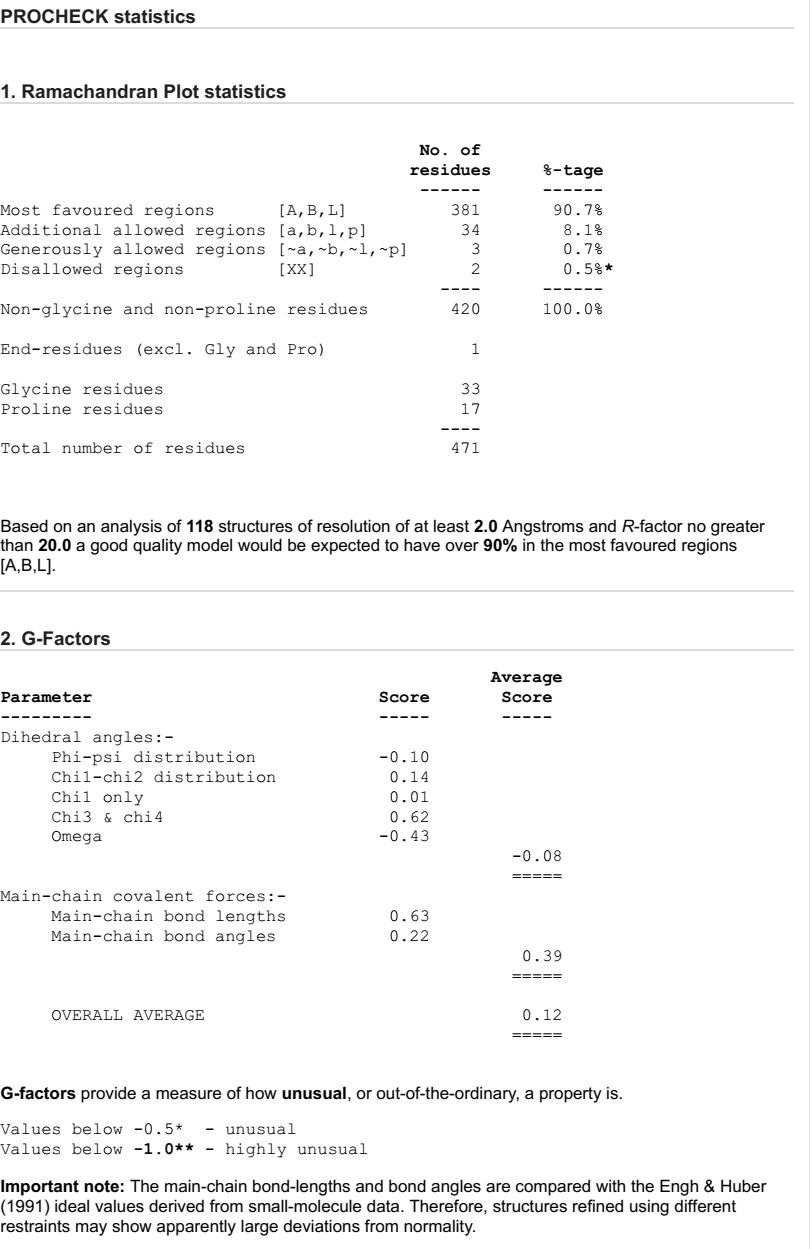


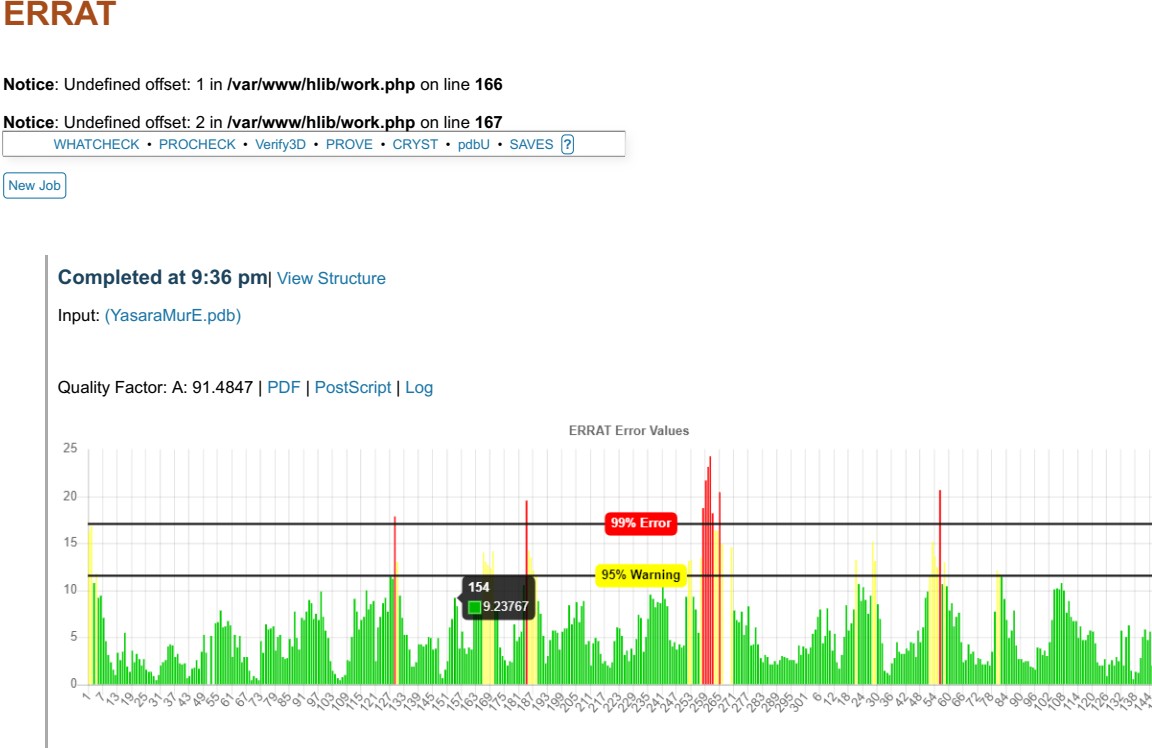


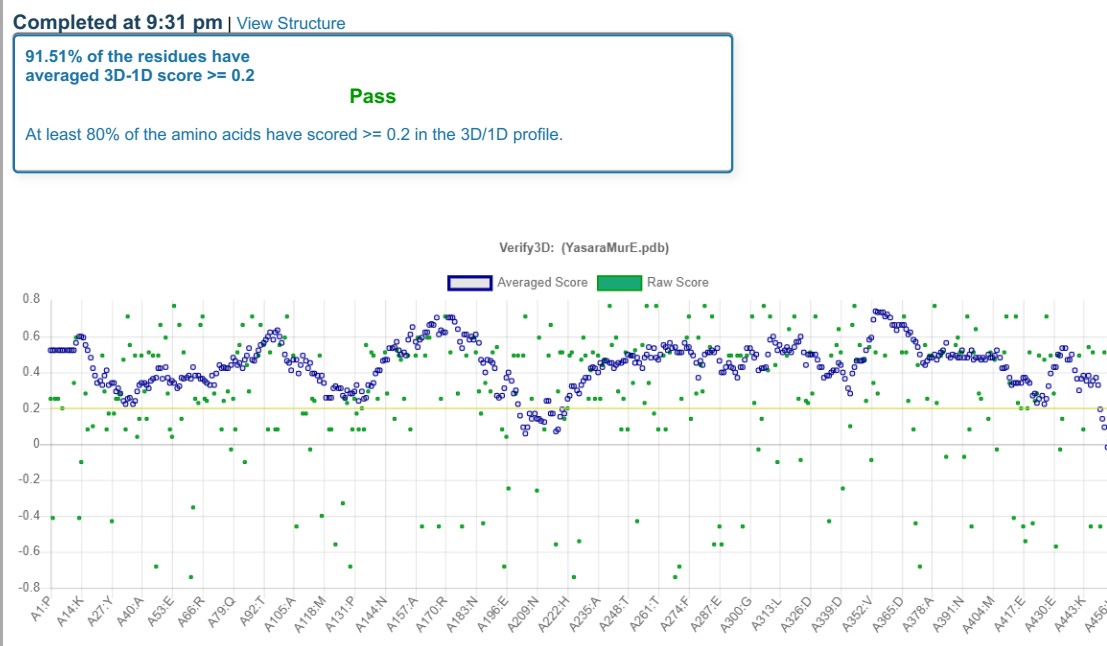


**MurG structure from *A. pittii* evaluation results**


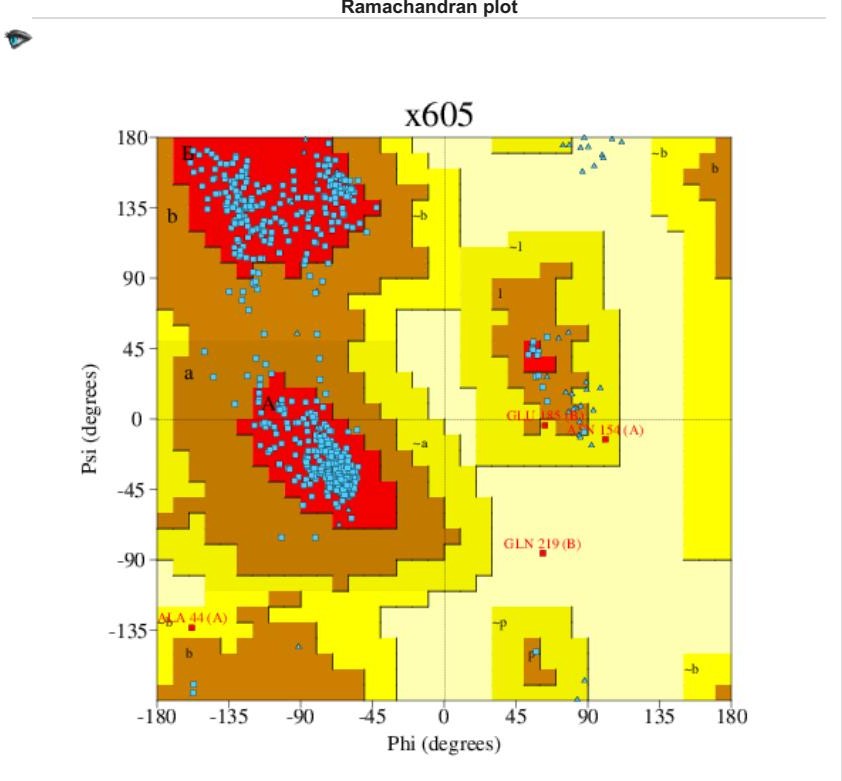


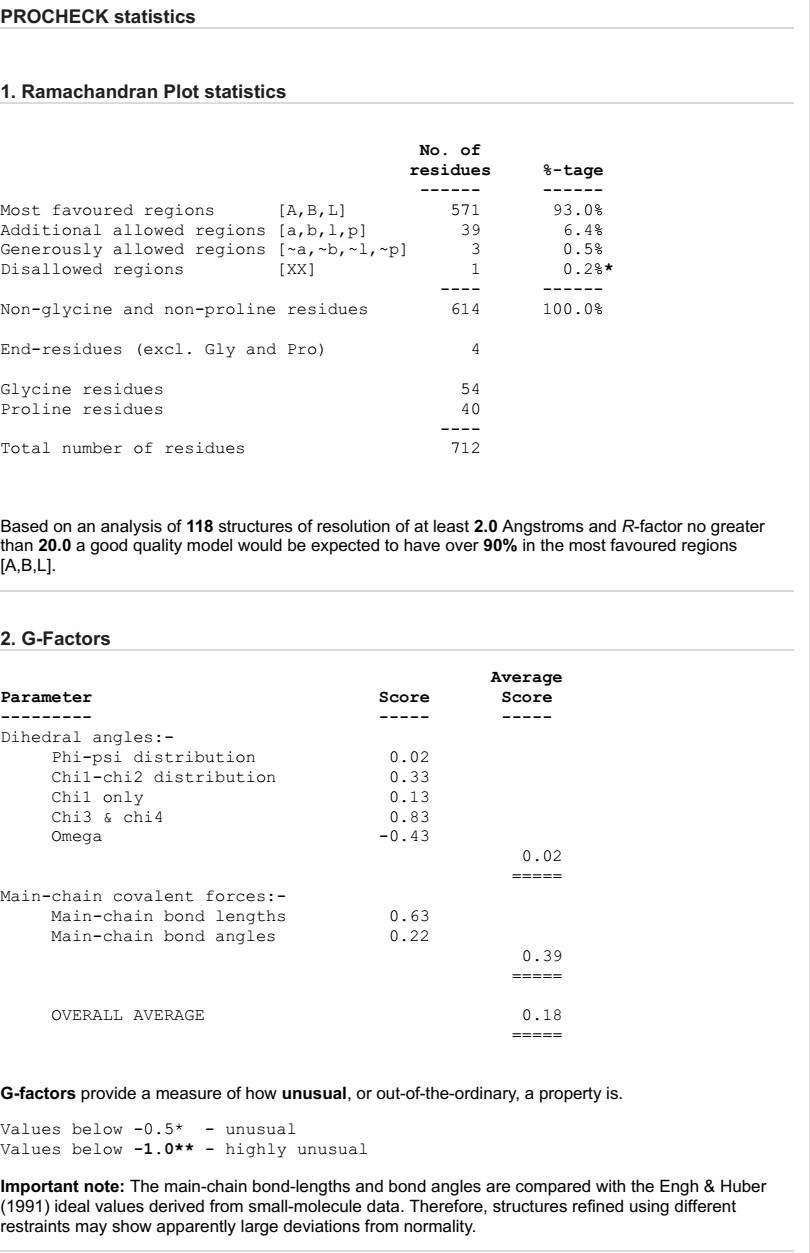


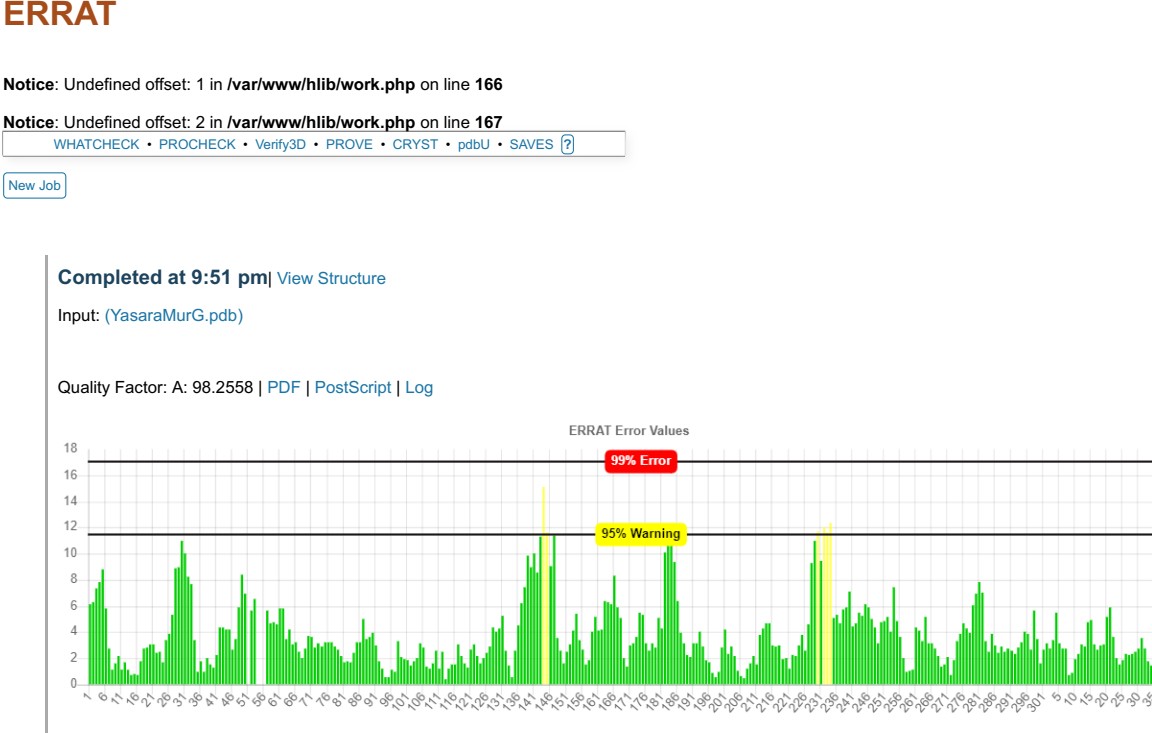


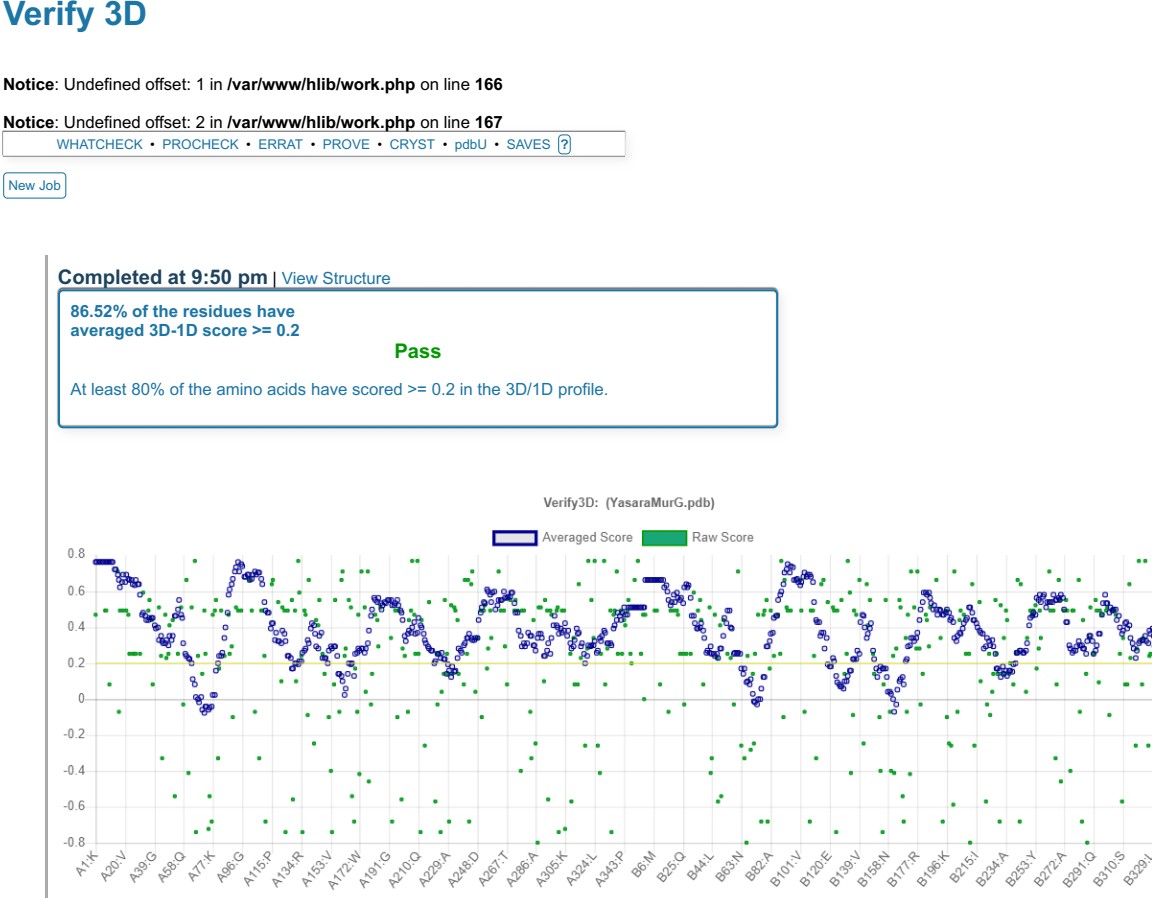

Supplement: Supplementary file 1 — Additional file 1: Phylogenetic analysis: Figure S1. Phylogenetic analysis of MurB Protein using UPGMA Method. Figure S2. Phylogenetic analysis of MurB using Neighbor Joining Method. Figure S3. Phylogenetic analysis of MurB using Maximum Parsimony Method. Figure S4. Phylogenetic analysis of MurB using Minimum Evolution Method. Figure S5. Phylogenetic analysis of MurB using Maximum Likelihood Method. Figure S6. Phylogenetic analysis of MurE using UPGMA Method. Figure S7. Phylogenetic analysis of MurE using Neighbor Joining Method. Figure S8. Phylogenetic analysis of MurE using Minimum Evolution. Figure S9. Phylogentic analysis of MurE using Maximum Parsimony method. Figure S10. Phylogenetic analysis of MurE using Maximum Likelihood Method. Figure S11. Phylogenetic analysis of MurG using UPGMA Method. Figure S12. Phylogenetic Analysis of MurG using Neighbor Joining Method. Figure S13. Phylogenetic analysis of MurG using Minimum Evolution Method. Figure S14. Phylogenetic analysis of MurG using Maximum Parsimony Method. Figure S15. Phylogenetic analysis of MurG using Maximum Likelihood Method. Figure S16. Multiple Sequence Alignment Eight Mur Family Proteins from Acinetobacter baumannii.Figure S17. Structure validation reports for MurB, MurE and MurG from different species [file 43141_2020_48_MOESM1_ESM.docx]
